# Meta-Analysis of *Drosophila* Circadian Microarray Studies Identifies a Novel Set of Rhythmically Expressed Genes

**DOI:** 10.1371/journal.pcbi.0030208

**Published:** 2007-11-02

**Authors:** Kevin P Keegan, Suraj Pradhan, Ji-Ping Wang, Ravi Allada

**Affiliations:** 1 Department of Neurobiology and Physiology, Northwestern University, Evanston, Illinois, United States of America; 2 Department of Statistics, Northwestern University, Evanston, Illinois, United States of America; Washington University, United States of America

## Abstract

Five independent groups have reported microarray studies that identify dozens of rhythmically expressed genes in the fruit fly *Drosophila melanogaster.* Limited overlap among the lists of discovered genes makes it difficult to determine which, if any, exhibit truly rhythmic patterns of expression. We reanalyzed data from all five reports and found two sources for the observed discrepancies, the use of different expression pattern detection algorithms and underlying variation among the datasets. To improve upon the methods originally employed, we developed a new analysis that involves compilation of all existing data, application of identical transformation and standardization procedures followed by ANOVA-based statistical prescreening, and three separate classes of post hoc analysis: cross-correlation to various cycling waveforms, autocorrelation, and a previously described fast Fourier transform–based technique [1–3]. Permutation-based statistical tests were used to derive significance measures for all post hoc tests. We find application of our method, most significantly the ANOVA prescreening procedure, significantly reduces the false discovery rate relative to that observed among the results of the original five reports while maintaining desirable statistical power. We identify a set of 81 cycling transcripts previously found in one or more of the original reports as well as a novel set of 133 transcripts not found in any of the original studies. We introduce a novel analysis method that compensates for variability observed among the original five *Drosophila* circadian array reports. Based on the statistical fidelity of our meta-analysis results, and the results of our initial validation experiments (quantitative RT-PCR), we predict many of our newly found genes to be bona fide cyclers, and suggest that they may lead to new insights into the pathways through which clock mechanisms regulate behavioral rhythms.

## Author Summary

Circadian genes regulate many of life's most essential processes, from sleeping and eating to cellular metabolism, learning, and much more. Many of these genes exhibit cyclic transcript expression, a characteristic utilized by an ever-expanding corpus of microarray-based studies to discover additional circadian genes. While these attempts have identified hundreds of transcripts in a variety of organisms, they exhibit a striking lack of agreement, making it difficult to determine which, if any, are *truly* cycling. Here, we examine one group of these reports (those performed on the fruit fly—Drosophila melanogaster) to identify the sources of observed differences and present a means of analyzing the data that drastically reduces their impact. We demonstrate the fidelity of our method through its application to the original fruit fly microarray data, detecting more than 200 (133 novel) transcripts with a level of statistical fidelity better than that found in any of the original reports. Initial validation experiments (quantitative RT-PCR) suggest these to be truly cycling genes, one of which is now known to be a bona fide circadian gene (*cwo*)*.* We report the discovery of 133 novel candidate circadian genes as well as the highly adaptable method used to identify them.

## Introduction

Most organisms exhibit rhythms of behavior and physiology that occur with “circadian” or daily periods. Such rhythms are driven by endogenous biological clocks regulated via the rhythmic expression of a core set of pacemaker genes [4–7]. A salient feature of many circadian genes, conserved across a wide span of evolutionary divergence, is the cyclic expression of their mRNAs [5,6]. Several studies have exploited this characteristic to identify novel clock-related genes.

Five such reports, based in the model organism Drosophila melanogaster (the fruit fly), utilize microarray technology to discover clock-related genes that exhibit cyclic mRNA expression in fly heads [1,8–11]. These cumulatively identify hundreds of rhythmic transcripts; yet, there is a striking lack of overlap among the lists of identified genes. This raises doubts as to the fidelity of reported expression patterns [12–14]. Given the importance of rhythmic transcription to circadian clock function, we revisited these studies, attempting to identify the root causes of existing incongruities and to find transcripts that exhibit *truly* cyclic expression. Our analyses suggest that compilation and statistical prescreening of all available data lead to substantial reductions in the false discovery rate (FDR). In addition, we find data quality and algorithm choice to play essential roles in determining the transcripts ultimately detected by any particular analysis. We introduce a novel procedure, as well as improvements to previously published techniques, that identify a core set of rhythmically expressed transcripts with high statistical fidelity. Intriguingly, our list includes 133 transcripts not found in the original reports.

## Results

### Comparison of Different Protocols and Results in Microarray Studies

Experimental approaches used to generate data were similar among the five fruit fly circadian microarray studies (Table S1). Wild-type *Canton S* (*CS*)*, yellow white* (*y w*)*,* or *cinnabar brown* (*cn bw*) strains (the latter two are mutants marked with pigmentation phenotypes, but are generally considered to be wild-type with respect to circadian behavior) were entrained in a 12-h light/12-h dark (LD) environment before release into constant darkness (DD). Samples of flies were subsequently collected over one to several days at regular intervals. Each sample was used to generate probes for a single Affymetrix *Drosophila* 1 oligonucleotide array. Sample hybridization, expression detection, and low-level chip analyses were performed according to Affymetrix guidelines and with Affymetrix software (MAS 4 or 5; McDonald et al. [8] being the single exception in which dCHIP software was used to produce average expression values). However, higher-level analyses, particularly the algorithms used to identify cycling expression profiles, differed greatly among the five reports.

To assess the degree of overlap among the studies, we compared the reported lists of cycling transcripts between any two, then among any three, four, and finally all five studies. Overlap between any two lists peaked at 27.8%. For any combination of three, four, or all five studies, overlap plummeted to 17.4%, 10.4%, and 9.7%, respectively (Table S2). The seven genes found in common among all five reports include most, but not all, of the known cycling circadian genes (*period* being the most obvious exception). Overlap values are substantially higher than those predicted by chance (Table S2; italicized values), but are low enough to reveal a surprising degree of disparity. This suggests one, or both, of two possibilities: that the different cycling gene detection algorithms employed introduce bias, or that the five datasets are genuinely inconsistent with each other. The former possibility has been suggested in the reports themselves and in several review articles [1,8,9,12–14]. To our knowledge, two other studies have suggested, but none have directly examined, the latter [3,15].

### Discrepancies among Genes Identified in the Original Studies Are Due to Inconsistent Algorithms and/or Inconsistent Datasets

To determine the contribution of algorithm dependent bias to differences in genes identified among the original reports, we analyzed each dataset with its native technique (the algorithm published with it) and the waveform correlation method described in McDonald et al. [8] (see Methods). Comparison of the two lists of cycling transcripts generated from each dataset reveals significant disparity, with an average overlap of just 21.8 ± 6.8% (mean ± standard error of the mean), a range of 12.5% to 41.7% (Table S3). These observations are consistent with the notion that algorithms introduce bias, recognizing different transcripts even when applied to the very same dataset.

A second potential explanation for the low-level overlap is that the five original datasets are genuinely inconsistent. Such inconsistencies can be attributed to several possible factors. While similar in a broad sense, the five studies do differ in seemingly minor details ranging from selection and treatment of fly populations to mRNA isolation, cDNA probe generation, chip hybridization conditions, and chip lot; even the use of different Affymetrix chip scanners has been shown to produce significant bias in expression profiling [16]. Differences of this nature (generally referred to as *lab-dependent* differences) are extremely difficult to quantify; yet, their contribution to end-of-analysis inconsistencies can be profound, the greater and/or more numerous the difference(s), the larger the inconsistencies. To determine if such differences could be observed among the five reports, we applied a single algorithm (our reproduction of the McDonald algorithm) to all five datasets and compared the lists of identified cycling genes (Table S4). This treatment removes algorithm-dependent bias, leaving lab-dependent differences as the only major source for inconsistencies in identified cycling transcripts. Surprisingly, we observed overlap among the identically derived lists to be even less than that observed among the originally published ones (Tables S2 and S4). The highest degree of identity observed between any two lists was 24.4%, with an average of just 11.4 ± 1.8% (range, 4.4%–24.4%), opposed to an average of 22.9 ± 1.2% (range, 16.5%–27.8%) observed among our comparisons of the original reports. Only three transcripts were found in common among all five reports, opposed to the seven found among the originally reported cycling transcripts. These results strongly suggest that the primary data are inconsistent, presumably due to significant lab-dependent differences, yielding unique lists of genes even when data are identically processed.

The successful identification of similar lists of genes appears to depend on consistency both with respect to raw data and the methods used to process it. Irregularities in either or both, as are clearly observed among the five extant studies, lead to significant disagreement among the lists of identified cycling genes.

### A Novel Algorithm Based on the Compilation and Statistical Screening of Multiple Datasets Substantially Reduces the FDR

A potential explanation for the observed inconsistencies is the use of different fly strains [15]. At least three behaviorally wild-type strains were used among the five studies (*CS, y w,* and *cn bw*)*.* As we were primarily interested in identifying cycling transcripts that may play a role in pathways that mediate central clock output to animal behavior, we chose to examine array data only if it corresponded to strains that exhibit well-conserved behavioral rhythms. Comparing the behavioral profiles in LD and DD of the three experimental strains, we observed a relatively high degree of correlation between *CS* and *y w* strains (*r^2^* = 0.75), but *cn bw* flies were less well-correlated with *CS* (*r^2^* = 0.30) and *y w* (*r^2^* = 0.46; Figure S1). Based on these observations, our analysis included data from *CS* and *y w,* but not *cn bw.*

**Figure 1:**
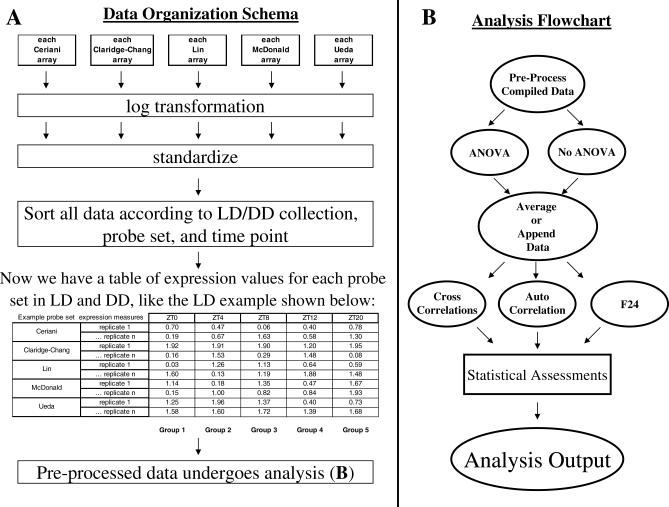
Analysis Outline Graphic depiction of the analysis framework we applied to the compiled and individual datasets. (A) Data organization and preprocessing. Expression data from each individual array from each of the five studies were transformed into log_2_ coordinates and standardized such that the mean expression for each array was 0 and the standard deviation was 1. Data were then sorted according to probe set and LD/DD time of collection. This resulted in a table containing all expression measures for each of the 14,010 probe sets. (B) Flow diagram of all analyses that followed preprocessing. After preprocessing, time series data were optionally screened with ANOVA; all time series were appended and averaged to create appended and averaged datasets. These underwent three separate classes of analysis, (cross-correlation to a 24-h sin wave, an expression profile of *per* mRNA derived from So et al. [21], a profile derived from wild-type LD locomotor behavior, or a profile derived from wild-type DD locomotor activity, autocorrelation, and fast Fourier transform (F24) analysis as first described in Claridge-Chang et al. [1]).

Subsequently, our analysis consisted of five major steps: (1) ANOVA-based statistical prescreening for significant time-dependent changes in expression; (2) correlation analyses assessed via the Pearson correlation coefficient; and (3) a Fourier transform–based technique (F24) followed by (4) permutation-derived *p*-value assessment of all analyses (cross-correlation, auto-correlation, and F24 (Figure 1). (5) Steps 1–4 were performed on randomized datasets to approximate the FDR. Our analyses were also assessed via a simple power statistic.

To limit the impact of lab-dependent differences, we compiled data from all of the reports, creating a single *meta*-dataset. We expected truly cycling patterns to reinforce each other, whereas those found to be cycling in individual reports (most likely due to errant noisy signals) would cancel and thereby suppress each other. This allowed us some measure of control over experimental noise without having to identify its precise causes.

Data were compiled, sorted according to the LD or DD time of collection, and preprocessed with a log transformation [1–3] and subsequent standardization procedure [17]. The resulting dataset exhibited a normal distribution with an average expression of 0 and variance of 1 for each array (see Methods; Figure S2, LD data in part A, DD data in part B). These procedures removed several hundred transcripts; more than 12,000 were left to consider. Even with a relatively restrictive *p*-value of 0.05, a dataset of this size would be expected to produce some 600 false positives (the *p*-value times the total number probes to be tested) [18]. Such a number of false positives (it is interesting to note that fewer than 600 unique transcripts were identified as cycling among the five original reports) could obscure results, making determination of *truly* cycling transcripts difficult via the reduction of statistical confidence [18]. Application of a priori filters is a practical solution to this problem. Based on sound assumptions, these can reduce the number of comparisons, while at the same time enriching the remaining population for the characteristic(s) of interest [19,20]. We reasoned that *truly* cycling genes would necessarily exhibit statistically significant changes in expression versus time. To identify transcripts that exhibited this trait, we applied an ANOVA filter after data were preprocessed and sorted (Figure 1; Methods). Our ANOVA screen identified transcripts that exhibited statistically significant changes in expression, and removed those that did not from further consideration (see Methods). This produced a set of 372 transcripts that exhibited significant time-dependent changes in expression in LD, and a set of 679 transcripts in DD (Table S7).

All data were then processed via two separate pathways. In the first, expression values were grouped by timepoint and averaged together (eight to 13 arrays per timepoint) such that each probe set was represented by two six-point time courses, one derived from all available LD data, the other from DD data. We reasoned this procedure would suppress noise (the distribution of noise would be random, averaging would tend to suppress it) while concomitantly reinforcing *true* signals. However, this technique exhibited a significant shortcoming; it essentially ignored variation within each timepoint. To account for this limitation, we processed all data via a second route. After the ANOVA screening, data were appended such that two profiles, 8–13 d long, one derived from LD data, the other from DD data, were produced for each probe set. In this dataset, variance was not suppressed by averaging. As the majority of our post hoc tests (autocorrelation being the only exception) were insensitive to the order of the days of data in the appended profile—a 3 d–long profile would give the same result regardless of the ordering of the days for all cross-correlations and F24—we chose to use a single ordering of the days for all post hoc tests. Before our post hoc tests were applied to the appended datasets, the data for each day in the multiday profiles (data for each single day came from a single report/lab) underwent one additional standardization procedure such that the data for each day would exhibit a mean of 0 and variance of 1. This was intended to minimize any artifactual expression level differences that may have been present in data from each report even after our initial transformation and standardization procedures. It also allowed for roughly equal weighting of each day in the expression profile; that is, expression values for each day were constrained such that their location and scale would be the same, while profile contours remained unchanged.

To characterize the expression patterns of transcripts that passed the ANOVA screen, we performed a series of post hoc analyses: cross-correlation to four model waveforms, a sin wave, a previously reported characterization of *per* expression [21], wild-type LD *CS* behavior, and wild-type DD *CS* behavior; we also employed an autocorrelation technique. Whereas the standard application of autocorrelation would have required two or more days of data [2], we used a method that allowed autocorrelation to be applied to a single day of data. Autocorrelation requires two representations of the same profile. The first representation (A) is held constant as the second (B) is shifted out of register with it one timepoint at a time. With each shift, data points at the extremities are “lost” as they are moved beyond the point where they can be paired with a point from the other representation. To avoid this loss of data, we considered each representation circularly. The point lost from the end of B after a single shift would be moved to B's beginning such that all points would be compared to all points at each registration (e.g., six timepoints at five nonidentical registrations for the six-point-long averaged time courses). In addition, we considered the absolute, rather than raw, value of the Pearson correlation coefficient. This allowed us to detect 24-h rhythmicity with a single day of data—two 24-h sin waves exactly 12 h out of phase with each other will produce an absolute Pearson correlation coefficient of 1.

Two tests were used to approximate the statistical significance of our post hoc tests. First, to determine power (essentially, a measure of how well each method detected known cycling genes), an empirical procedure was used. We tested each method's ability to detect six well-known cycling circadian genes (*Clk, cry, per, tim, vri,* and *Pdp1;* see Methods). The power statistic was calculated as *n*/6, *n* being the number of the known cycling genes detected. Next, to determine the FDR, we utilized a method similar to that employed by significance analysis of microarrays (SAM [22]). After each post hoc analysis was applied to a particular dataset, the data were randomized (within each transcript), and the post hoc test was repeated. Genes detected as cycling in the randomized dataset were scored as false positives. The FDR was calculated as the ratio of false positives to positives detected in the original (nonrandomized) dataset. Figure 1 presents a schematic diagram of our data organization and preprocessing steps (Figure 1A) as well as a flow diagram of all subsequent analysis procedures (Figure 1B).

To determine if our compiled dataset would yield results that were more statistically reliable than those derived from any single dataset, we applied our technique to the LD data from each report, provided reports contained replicate arrays for each timepoint (a requirement for ANOVA prescreening), and compared results with those obtained from the meta-dataset. First, we examined how each dataset performed with respect to its FDR. We found the compiled dataset to exhibit an FDR lower than those generated from the original, individual datasets (Table 1; list IDs refer to lists of overlapping genes—identified by Affymetrix probe set ID—presented in Dataset S1, an addendum to Table 1), suggesting that compilation leads to results in which false positives are suppressed. Intriguingly, we also discovered that ANOVA prescreening drastically reduced the FDR in the individual and compiled datasets. We also assessed datasets with respect to power. Power observed in the appended LD meta-dataset was as good or better than that observed in any individual set. Maximum power was observed under F24 analysis in the meta-dataset, but in only two of the three individually tested datasets. Other methods exhibited much lower power values. It is possible that differences in power between individual reports may be due to differences in data quality, but such determinations should be treated with skepticism as they rely on a very small sample size (Table 1). To determine if any one of the original datasets correlated better than the others when compared with the meta-dataset, we directly compared each originally published list with the list of transcripts we identified in our LD meta-dataset. With respect to number of cycling transcripts found in common, we observed each of the original reports to perform similarly; between 27 and 38 transcripts were found in common with the 177 identified in our analysis of the LD meta-dataset, but very few transcripts were found in common between two or more of the original reports and/or the meta-dataset. However, when we considered the number of genes found in common as a percentage of all those found in any one of the original reports, there appeared to be some differences among the five original reports: Claridge-Chang et al. [1], 24%; MacDonald et al. [8], 26%; Ceriani et al. [11], 27%; Ueda et al. [10], 30%; and Lin et al. [9], 43% (Table S6).

**Table 1:**
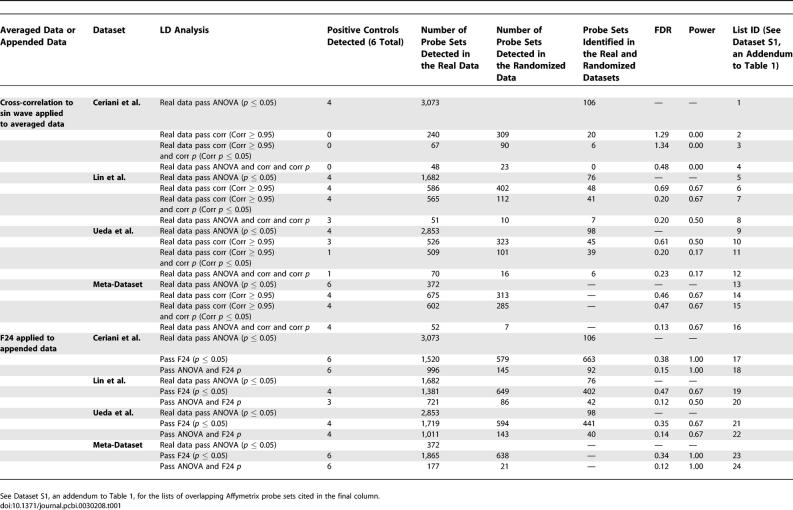
FDR/Power Analysis of Compiled and Individual LD Datasets

In our initial post hoc analyses of the DD data, FDRs were found to be much less favorable than those exhibited by the corresponding LD analyses. This is not surprising, as endogenous transcript cycling is known to be less robust and much less widespread than that driven and/or enhanced by the presence of strong entraining stimuli, particularly light cues [5]. Based on these observations, we decided to focus the majority of our efforts on the LD datasets.

Under our initial analysis conditions, FDRs were higher than the generally desired value of 0.05 or less. Even our best performing analyses, sin-wave cross-correlation of averaged data and F24 of appended data, exhibited FDRs of 0.13 and 0.12, respectively. To determine if adjustment of our selection parameters could improve the FDR, we performed a simple FDR optimization on the F24 procedure, chosen as it detected the largest number of genes with the most favorable FDR. We considered three groupings of the appended LD data: transcripts that passed the ANOVA screen (ANOVA *p* ≤ 0.05), transcripts that did not pass the ANOVA screen, and a third grouping that considered all transcripts.

In each group, we varied the threshold of the F24 permutation–derived *p*-value and observed the impact on the FDR. In the ANOVA-screened group, an F24 permutation *p*-value of 0.02 or less produced FDR values of 0.05 or better with almost no impact on the number of transcripts detected (167 versus 177), and no impact on power (power = 1). In the combined grouping, an FDR of 0.05 or better required an unreasonably stringent *p*-value of 0.0004 or less. The group that failed to pass the ANOVA screen also failed to produce an FDR ≤ 0.05 at our most stringent *p*-value, 0.0001 (Figure S4). Within our meta-dataset, ANOVA prescreening improved the FDR, and its application was necessary to achieve acceptable FDR values with a reasonable degree of stringency (i.e., *p* ≤ 0.02).

To determine the extent to which our ANOVA prescreening procedure improved the FDR, we performed the two most successful (with respect to number of genes identified and the associated FDR) analysis procedures (sin-wave cross-correlation applied to the averaged LD dataset and F24 analysis applied to the appended LD dataset) with and without ANOVA prescreening. ANOVA prescreening significantly improved the FDR with little or no reduction observed with respect to power. In both cases, removal of the ANOVA prescreening procedure increased the number of identified genes, but at significant detriment to statistical fidelity (sin-wave cross-correlation to averaged data, 52–probe sets FDR = 0.13 with ANOVA, 602–probe sets FDR = 0.47 without; F24 to appended data, 177–probe sets FDR = 0.12 with ANOVA versus 1,688–probe sets FDR = 0.38 without; Table 1). In an expanded analysis, we examined the effect of ANOVA prescreening on the LD and DD datasets. We found ANOVA to significantly improve the FDR except where the number of identified cycling transcripts was very small (*n* ≤ 10). In these cases, ANOVA seemed to have no discernable effect (Table 2).

**Table 2:**
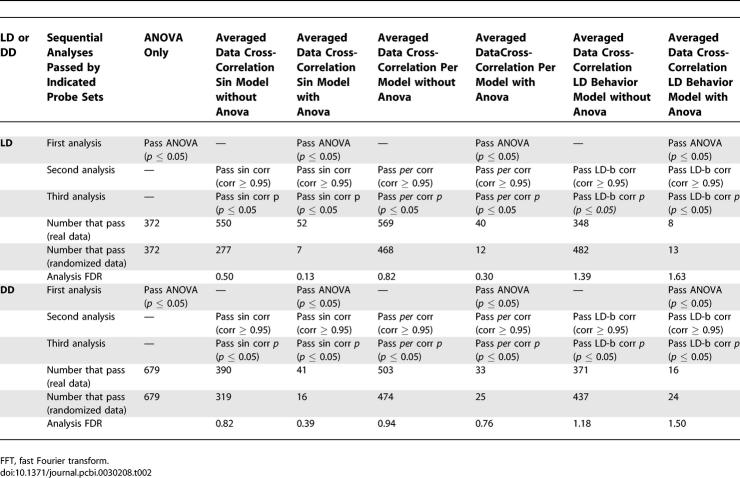
FDR Analyses of Post-Hoc Tests

**Table 2:**
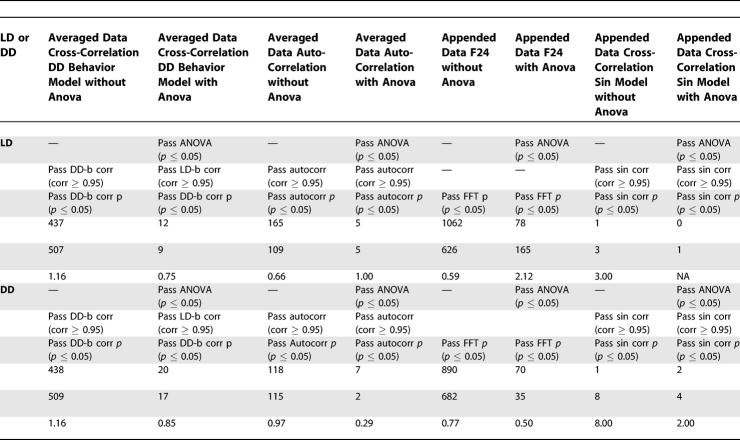
Extended.

**Table 2:**
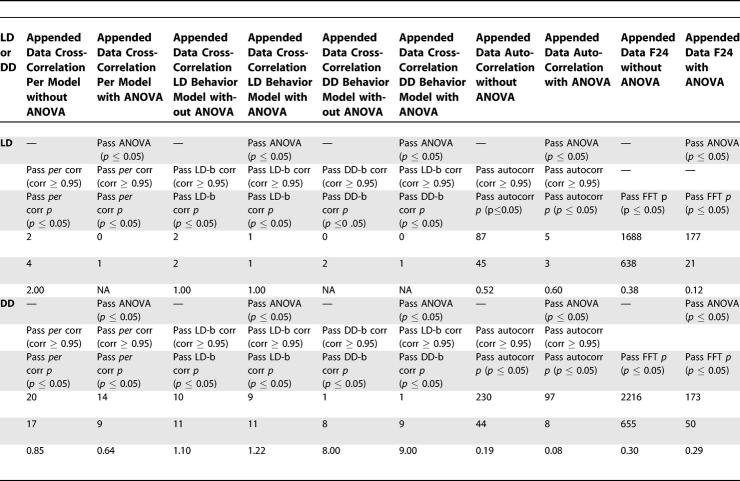
Extended.

To determine if data compiling or ANOVA prescreening could be of benefit to other previously reported cycling-gene–detecting algorithms, we applied both procedures to the McDonald and F24 algorithms. We determined FDRs for both methods with and without application of the ANOVA screen. Under both analyses, the ANOVA prescreening procedure significantly reduced the FDR, from 0.57 to 0.14 for the McDonald algorithm and from 0.34 to 0.12 for F24 analysis (Table 3). The number of identified transcripts was reduced, but power remained unaffected, and the ANOVA screen passed all known cycling genes, suggesting the transcripts it rejected to be false positives. Our tests indicate that regardless of subsequent algorithmic steps, prescreening of the data has a drastic effect on the FDR, and could potentially be of benefit to other similar analyses. Compilation of data also leads to improvements in observed FDRs, but these appear to be less drastic than those introduced by the application of ANOVA prescreening (Table 1).

**Table 3:**
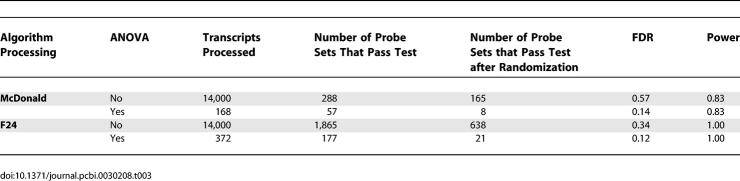
McDonald and F24 Algorithm Processing of the Meta-Dataset with and without ANOVA Prescreening

### The Number and Identity of Cycling Genes Heavily Depends on the Cycling Model Used to Fit the Data

The originally published reports utilized three distinct classes of cycling transcript detection algorithms: cross-correlation to sin-waves [10,11], autocorrelation [1,9], and Fourier transform analysis [1]. We performed all three classes of analyses on our averaged and appended meta-datasets with and without ANOVA prescreening, followed by rigorous statistical testing (see Methods). In addition, we used cross-correlation to compare expression profiles with models derived from experimental observations (per mRNA expression and LD and DD locomotor behavior; see Figure S3). These novel expression models were constructed from a published *period* mRNA expression profile [21] and our own unpublished behavior data.

Results strongly depended on the method and/or expression models employed. F24 analysis, when applied to the appended dataset, appeared to exhibit the greatest sensitivity, detecting a greater number of transcripts with a more favorable FDR than any other technique. However, some transcripts identified by cross-correlation to our experimentally derived expression models were not detected via F24, suggesting that some cyclic expression patterns are poorly matched by sinusoidal waveforms (Table 4; for a complete listing of all ANOVA and post hoc scores, see Dataset S2, a supplemental addendum to Table 4). Reduction in the stringency of our detection thresholds allowed F24 to detect some of these transcripts, but at significant detriment to the FDR (unpublished data). Surprisingly, the results of our initial sin-wave cross-correlation poorly matched those of the corresponding F24 analysis. This was unexpected, as the two techniques are essentially equivalent [2]. The source of this discrepancy was a threshold cutoff applied to our sin-wave cross-correlation method (transcripts had to exhibit an absolute Pearson correlation coefficient of 0.95 or better) that had no equivalent step in F24. When this step was removed from our analysis, agreement between the F24 and sin-wave cross-correlation results was found to be nearly 100% (174 of 177 possible matches; unpublished data).

**Table 4:**
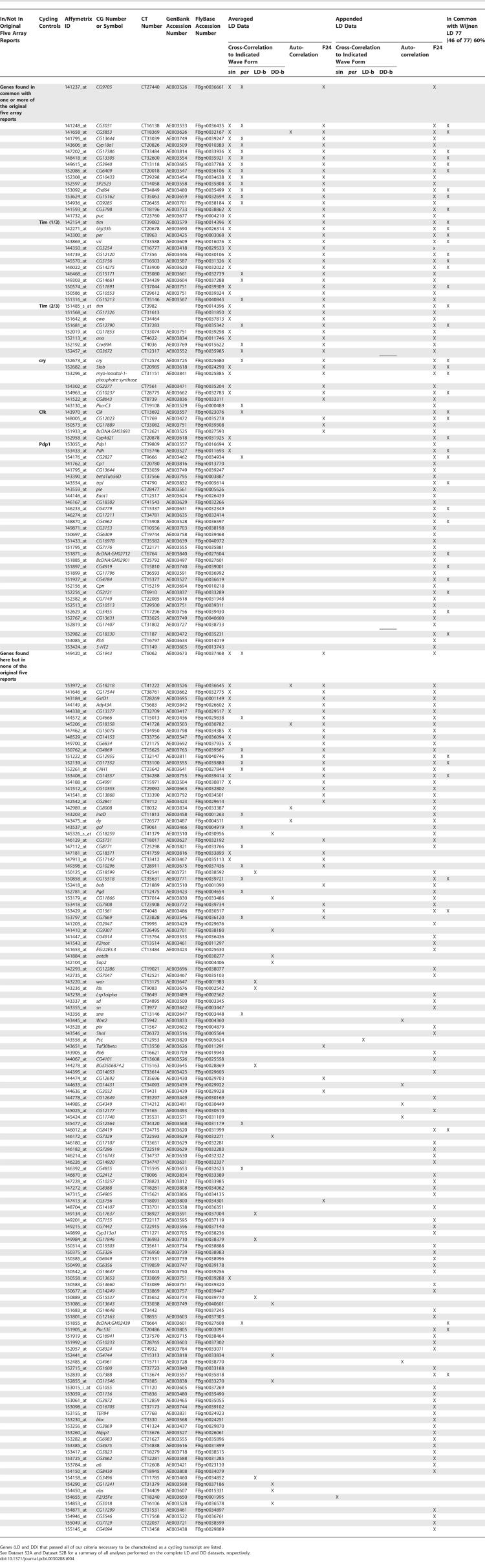
Identified Cycling Transcripts

While the majority of transcripts were identified either by sin-wave cross-correlation or F24 analysis, a small group was not. A total of 21 genes were discovered by one or more of our novel waveform cross-correlation analyses: eight by the LD behavior waveform, 12 by the DD behavior waveform, and 17 via the per expression waveform (Table 4). All 21 of these genes were found among our list of novel genes; none were detected as cycling in the original five reports.

**Figure 2:**
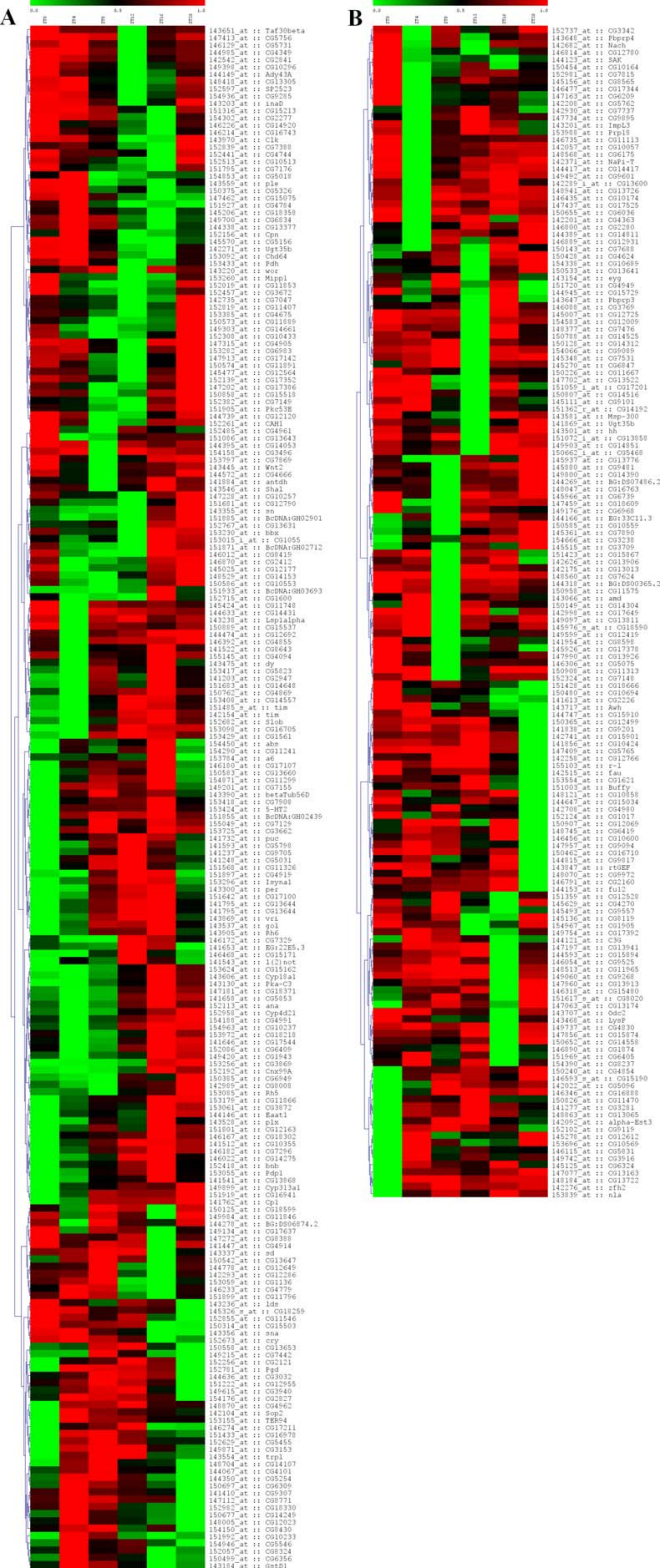
Cluster Analysis of LD Data Hierarchical clustering (LD data only: expression versus time) of genes that passed our ANOVA screening procedure is presented. The expression of all genes was normalized from 0 to 1. Red represents expression troughs; green, expression peaks. Genes are identified by symbol or CG number. (A) Characterized transcripts. Genes that passed the ANOVA prescreening procedure, and one or more of our post hoc tests. These genes are further described in Table 4. (B) Uncharacterized transcripts. Genes that passed our ANOVA screening procedure but none of our post hoc tests.

These results suggest that use of a single cyclic expression detection technique is not sufficient to identify all cycling patterns. Even with our battery of techniques, we were unable to characterize nearly half (160 of 372) of the LD profiles that exhibited significant changes in expression with respect to time as assessed by the ANOVA screen (Figure 2). These may be characterized by the use of additional expression models and/or reduction in the stringency of our initial selection criteria.

### Novel Cycling Genes

Under our initial analysis of the LD data, a total of 214 probe sets passed all selection criteria for one or more of our post hoc tests. Of this group, 81 were in common with one or more of the original five reports, including the known cycling circadian genes *Clk, cry, per, tim, vri,* and *Pdp1.* Remarkably, 133 transcripts identified in our analysis were not found in the original reports, including 21 that no sin-wave–based method was able to detect. Nine members of this novel group were found in a similar meta-analysis conducted by Wijnen et al. [3] (Table 4, last column), but in none of the original five reports.

To examine the LD data more closely, we prepared cluster dendrograms [23,24] of the transcripts that passed our ANOVA prescreening procedure (see Methods; Figure 2). Examination of the expression profiles reveals a wide assortment of patterns not detected by our analyses (present in Figure 2B, absent in Figure 2A), further evidence that a limited number of correlation techniques and/or expression models likely fails to detect a large number of robustly cycling expression patterns.

**Figure 3:**
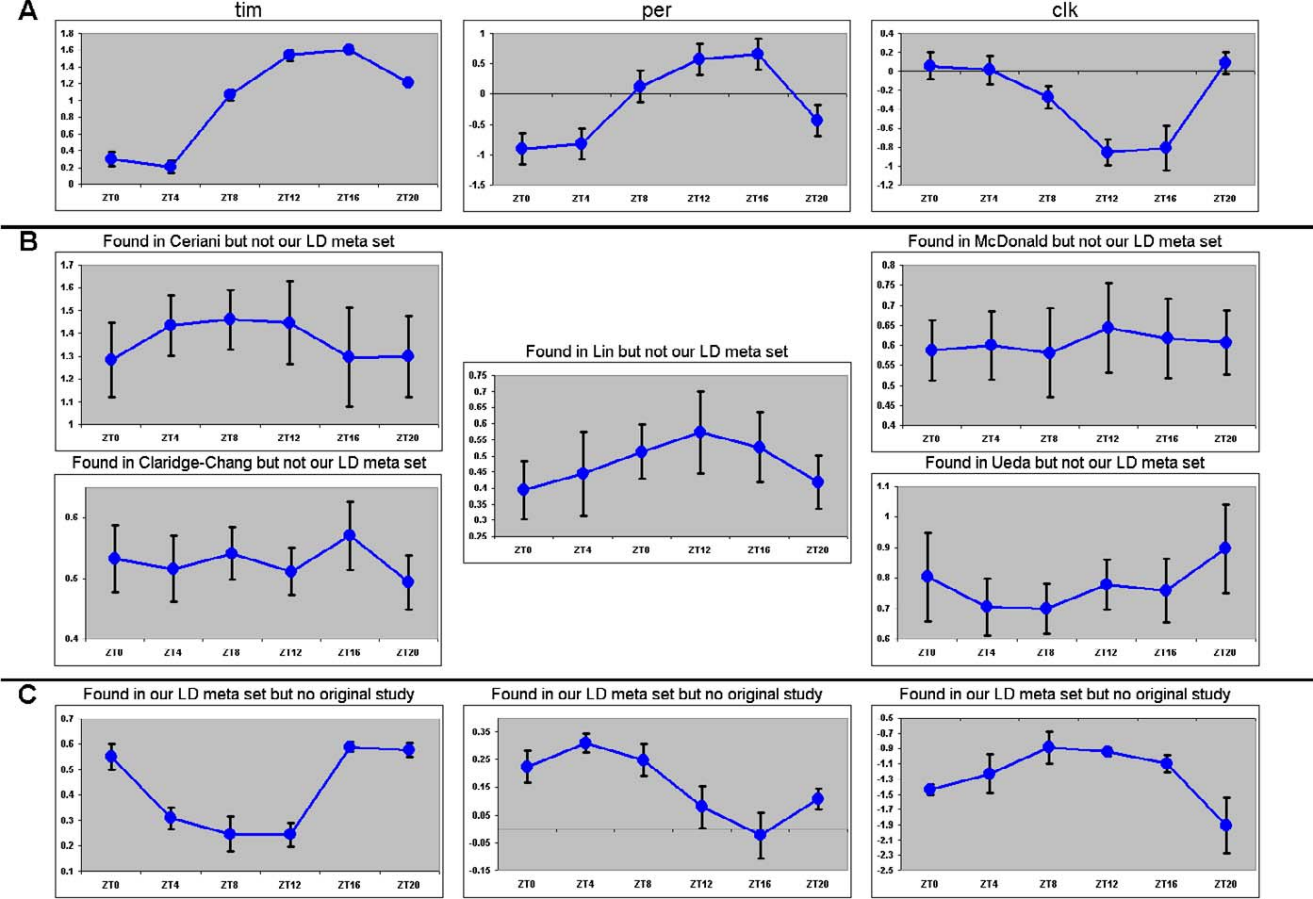
LD Expression Traces Plots are LD expression versus time. Dots represent the averaged standardized expression for all arrays at the indicated timepoint. Error bars indicate the standard error of the mean. Transcripts are referenced by symbol or Affymetrix probe set. (A) Traces of known (class I) cycling genes, a random selection of genes detected by our analysis and one or more of the original reports. (B) Traces of genes missed by our analysis (class II). One example was randomly chosen from each of the five reports. Report author is indicated in parentheses. (C) Novel genes (class III). A random selection of genes detected by our analysis, but none of the original analyses. See Dataset S3, the interactive supplemental addendum to Figure 3: it allows the viewer to observe and print every profile in our averaged LD meta-dataset.

To examine all LD data considered for a randomly selected number of genes, we plotted the average standardized expression values for all arrays at each of the six timepoints for three separate classes of transcripts. Class I transcripts were those found in the majority (three or more) of the original reports that were also detected by our analysis (Figure 3A). Class II consisted of genes identified as cycling in just one of the original reports that failed to be detected as cycling by our analysis (Figure 3B). Class III included those transcripts that were identified as cycling by our technique, but did not appear as cycling in any of the original reports (Figure 3C). Classes I and III appear to be robustly cycling, whereas class II exhibits no significant rhythmicity. This is consistent with our notion that transcripts identified in just one study are likely to be false positives, whereas those found in multiple reports or our compiled datasets are more likely to be bona fide cycling genes. Plots of the average standardized LD expression data for all genes (listed by Affymetrix probe set ID) can be produced from Dataset S3, a supplemental addendum to Figure 3.

### RT-PCR Confirmation of Cycling Transcripts

**Figure 4:**
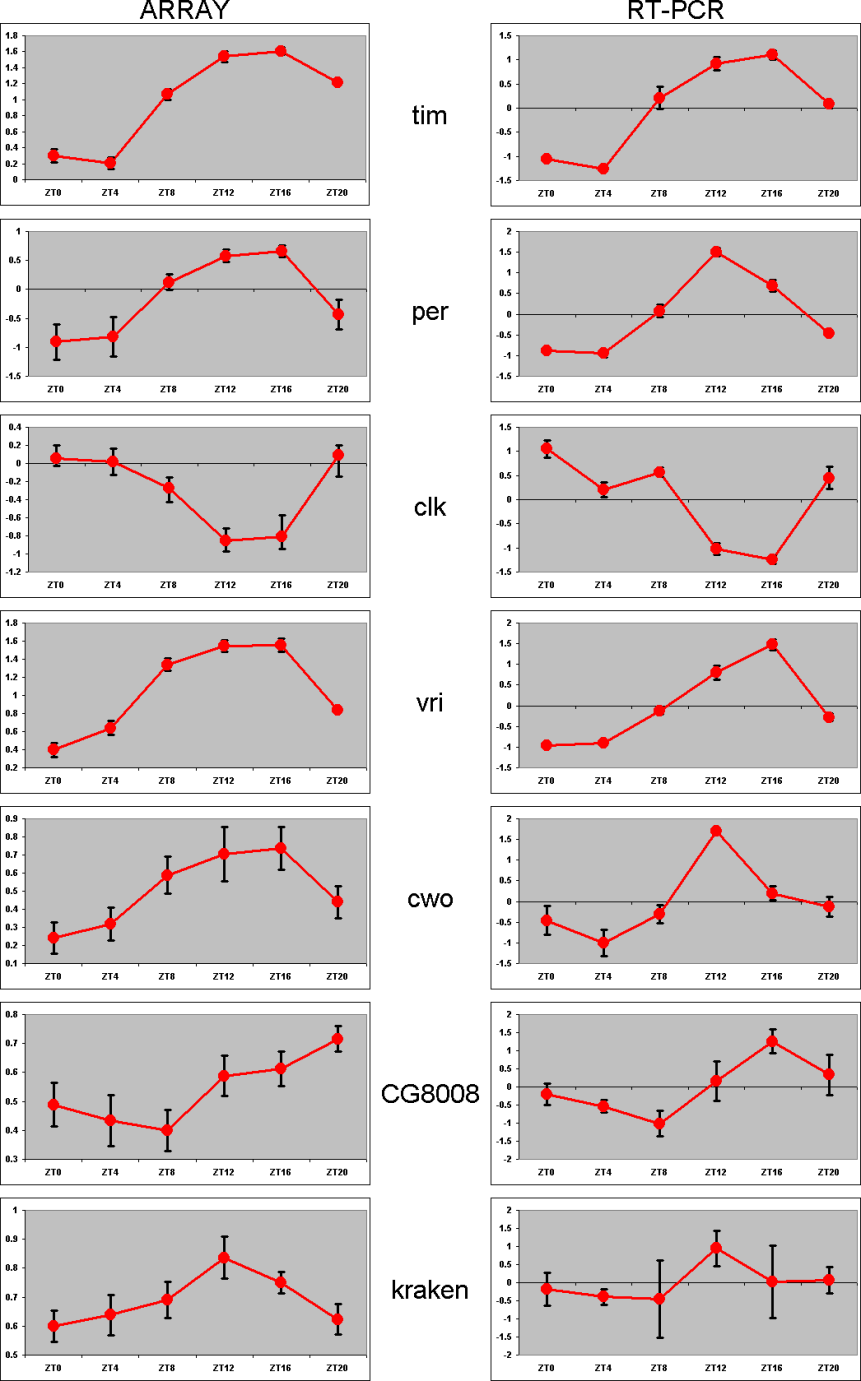
RT-PCR of Selected Genes LD expression array data (left column), and the compiled quantitative RT-PCR results (right column) of six genes. Error bars indicate the standard error of the mean (eight to ten replicates per timepoint for the array data, three to five replicates per timepoint for the RT-PCR data). See Table 5 for results of our algorithmic analysis of these RT-PCR data. RT-PCR data for *CG17100* (*cwo*) were adapted with permission from Lim et al. [26]; all other data are unique to this study.

To determine if the expression profiles of our newly discovered genes were indeed cycling, quantitative RT-PCR was used. We randomly selected three genes, two from our initial analysis (*CG8008* and *CG17100*: *CG8008* was found in none of the original reports, whereas *CG17100* was found in Lin et al. [9] and Ueda et al. [10], but in no other report we are aware of), and another detected under less stringent criteria (*kraken*)*.* While this manuscript was in preparation, three separate reports confirmed not only that *CG17100* (i.e., *clockwork orange* [*cwo*]) exhibits cyclic expression, but that it is a bona fide member of the circadian machinery [25–27]. A cursory examination revealed phase and amplitude values to be comparable between the compiled microarray and RT-PCR expression data for *CG8008* and *CG17100,* but not *kraken.* In addition, we performed RT-PCR on a group of four genes detected in our meta-analysis (well-known cycling circadian genes *tim, per, clk,* and *vri*) and one or more of the original reports (Figure 4). We assessed cycling of all RT-PCR–derived expression profiles with the same method applied to the microarray data (Table 5). For six of the seven genes tested, cycling in the RT-PCR data was confirmed under one or more of our analysis procedures. This included *CG8008* and *cwo. kraken* failed to pass our analysis procedure, not surprisingly, as it was detected under less stringent conditions, suggesting it to be a likely false positive.

**Table 5:**
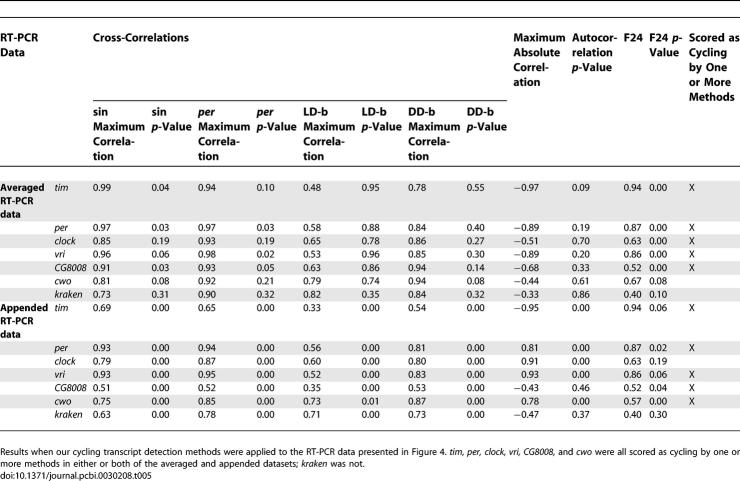
Cycling Transcript Detection Algorithm Processing of RT-PCR Data

## Discussion

This study achieved three objectives. We identified major sources of variation among the five original *Drosophila* circadian array papers, developed an analysis technique to compensate for these, and utilized it to re-examine the compiled data, identifying several novel cycling genes. Our method can easily be adapted to other similar datasets [28–31]. Furthermore, the general principles of data standardization and statistically based prescreening it employs are applicable to most any array-based study. In particular, ANOVA prescreening led to significant improvements in the FDR of our and two previously reported cycling expression detection methods, the McDonald algorithm [8] and F24 analysis [1–3].

Recent reports have identified two major sources of variation common to all microarray-based studies [15,16,32–38]: the use of different sample preparation protocols and platforms/equipment (the lab effect), and the use of different algorithms for expression measure condensation and higher level characterization of expression patterns. While algorithm-based differences among the five fruit fly circadian array reports have been under scrutiny for some time, the impact of the lab effect has only been considered more recently [15,32,33]. No doubt, this is due in large part to the enormous technical difficulty imposed by any attempt to quantify the almost innumerable differences that can occur among nominally identical studies. These differences can introduce sources of inconsistency at every step of any array study, from experimental details: animal selection and treatment, mRNA isolation, cDNA probe generation (especially if a procedure with multiple amplification cycles is selected), chip lot selection, chip hybridization, scanner selection, etc.; to any and all subsequent computational procedures: condensation algorithm selection/processing, pre hoc and post hoc statistical tests, etc. Consistent with these observations, we identified two sources of variation among the five reports analyzed. Application of different algorithms to the same dataset produced unique lists of identified cycling genes, indicating the presence of a strong algorithm-dependent bias. Conversely, when a single algorithm was applied to each of the datasets, results were also inconsistent, indicating the presence of significant lab-dependent inconsistencies.

The meta-dataset consisted of values compiled from all five reports after data were culled with respect to strain, log-transformed, and standardized. While use of robust multiarray average (RMA) processed data would have been preferable to that generated by the more outdated methods used in the original five reports (primarily Affymetrix MAS 4), *.CEL level data, a requisite for RMA processing, for several of the original reports were not publicly available. Expression values derived from average difference scores, the metric used in MAS 4, and RMA-based algorithms cannot easily be compared; differences between the two families of algorithms are significant. As a compromise for the sake of consistency, we used average difference scores, as these were readily available for all five reports.

We reasoned that differences in expression among reports due to the lab effect would be random, and hence suppressed by compilation, whereas true differences would appear in multiple reports and be reinforced by compiling. Variation due to the application of different algorithms was accounted for by applying a single analysis method to all data. Application of our method to the compiled dataset revealed FDRs much lower than those observed when our method was applied to data from several of the original datasets (Table 1).

Our analyses are based on methods described in Blalock et al. [19,20], Tusher et al. [22], and Wijnen/Claridge-Chang et al. [1–3]. The fundamental difference between it and previously reported attempts to identify cycling genes in D. melanogaster is the application of an a priori ANOVA-based screening procedure to drastically reduce the number of subsequent tests, concurrently reducing the number of expected false positives. Although three of the original studies used prescreening procedures (Table S1), these tended to be fairly mild, removing no more than a few thousand transcripts based on ad hoc assumptions or arbitrary expression thresholds with little consideration paid to the statistical significance of expression values. Our ANOVA screen identified just 372 transcripts in LD that exhibit significant changes in expression over time. Application of correlation analyses at the α = 0.05 level to this reduced dataset would be expected to produce just 19 chance positives, as compared to the ≈700 (the original reports cumulatively identify less than 600 cycling transcripts) expected from analysis of the unscreened dataset (14,010 transcripts), a clear improvement.

Whereas the original reports chose cross-correlation to sin waves, autocorrelation, or Fourier transform–based methods, we applied all three strategies. In addition, we introduced experimentally derived model waveforms to our cross-correlation analyses as a means to detect biologically relevant expression patterns that deviate from sinusoidal waveforms. The majority of transcripts found to be cycling in LD were detected by one or more techniques; however, nearly 10% (21 of 214) were not. These were detected exclusively by correlation to our behavior and *per* expression–derived waveforms.

The selection of cyclic expression–detecting techniques we employed was more expansive than that described in any of the original reports; yet, it was still not sufficient to characterize the full set of transcripts that passed our ANOVA prescreening procedure (Figure 2). This observation suggests that additional methods and/or expression models may be necessary to characterize the multitude of unique cyclic expression profiles present in the compiled dataset, a solution others have also suggested [15]. Alternatively, it may be possible to detect an increased number of cycling genes through the reduction of our stringent statistical controls, as evidenced by our FDR optimization of F24 (Figure S4). However, gains with respect to the overall number of detected genes must always be weighed against increased detriment to statistical fidelity.

To combat the multiple testing paradigm common to all array studies, we introduce a multilevel system of statistical controls. Key among these is our a priori ANOVA screening procedure. It allows us to identify transcripts that exhibited significant changes in expression with respect to time, while eliminating those that do not from further consideration. For all correlation-based assays (cross-correlation and autocorrelation), we use three levels of statistical control: (1) ANOVA, followed by a (2) Pearson correlation coefficient threshold, and finally a (3) permutation-derived *p*-value. We find observed FDRs to be improved by the successive application of all three controls, but most significantly by the ANOVA step. Not surprisingly, we find application of ANOVA prescreening to significantly improve FDRs observed under the MacDonald and F24 algorithms (Table 3). Our work suggests that ANOVA-based prescreening can improve FDRs irrespective of whatever post hoc tests are subsequently employed.

Comparison of the results of this and a recently published Fourier-based meta-analysis of the same datasets [3] reiterates the necessity for a multiplicity of approaches to detect the greatest number of cycling genes with high statistical fidelity. Our initial analysis discovered some 214 transcripts cycling in LD; Wijnen et al. [3] find 77 with better statistical fidelity (FDR = 0.05 versus 0.12). Adjustment to our permutation-derived *p* threshold (from 0.05 to 0.02) produces a list of 168 transcripts with the same level of statistical fidelity (FDR = 0.05). A comparison of both FDR = 0.05 LD cycling lists reveals good, but by no means perfect, agreement —60% (46 transcripts) are found in common (Table 4, last column). It is encouraging to note that this degree of overlap is twice that observed between any other pair of reports (the five original reports, our and Wijnen et al.'s meta-analyses [3]), but it is fair to inquire why the parity was not even greater.

While methods invoked here and in Wijnen et al.'s meta-analyses [3] are similar (most strikingly, both utilize a priori filters to screen LD data: Wijnen et al. [3] use the Kruskal-Wallis test on four days of LD data, whereas we applied ANOVA to 9–10 d, depending on data available for each transcript), there are several obvious differences. As our primary interest is to identify cycling genes involved in clock-to-behavior output pathways, we culled data on the basis of conservation of locomotor behavior patterns among the various strains tested; Wijnen et al. [3] do not. The 2006 Wijnen study includes new array data that we did not consider. Our cross-correlation analyses consider several waveforms derived from experimental behavior and known gene expression patterns; these identified 21 genes that no sinusoidally dependent technique (sin-wave cross-correlation or F24) was able to detect. Last, and perhaps most significantly, Wijnen et al. [3] performed RMA to produce initial expression measures; *.CEL level data, a requirement for RMA processing, were not publicly available for all reports while this study was being prepared. The impact that selection of different expression measure condensation algorithms can have with respect to the lists of transcripts ultimately detected as cycling is well-known [15]. Average difference scores, available for all five reports, were used in this study for the sake of data-processing consistency. It is likely that any one or all of these differences could be responsible for the disparity present between this and Wijnen et al.'s meta-analyses [3].

As the discovery of 133 novel cycling genes (genes not detected as cycling in any of the original five reports) was not anticipated, we decided to validate the expression of three randomly selected, two from our initial set of 133 genes (*CG8008* and *cwo*) and one found in an analysis performed with a relaxed Pearson correlation threshold (0.90 instead of 0.95; *kraken*). Quantitative RT-PCR results (Figure 4 and Table 5) indicate that *CG8008* and *cwo* exhibit robust rhythms, while *kraken* does not. This is consistent with our analyses of the meta-array datasets (i.e., genes detected under our initial conditions proved to exhibit the expression patterns predicted by our analysis); a gene detected under less stringent conditions (rejected under our initial analysis) proved itself to be a false positive when its expression was assessed via RT-PCR. Further validation of our initial selection criteria (as applied to meta-array and RT-PCR data) was found when we assessed the expression patterns of *tim, per, clk,* and *vri* (four well-known, cycling circadian genes). Our method detected all four of these as cycling in the array and RT-PCR data (Figure 4 and Table 5).

That two randomly selected members of our stringently characterized genes exhibit strong cycling suggests that a number of our uniquely discovered genes may be bona fide cyclers, strong candidate genes for tracing the molecular pathways, still largely unknown, that mediate central clock signals to various behavioral outputs. Of particular interest in this respect are genes detected by our behaviorally derived expression waveforms (eight by the LD pattern, nine by the DD pattern). Intriguingly, nine transcripts in our novel set are also identified in the 2006 Wijnen et al. study [3]. We feel this illustrates the power of a combinatorial algorithmic approach; both methods identify these genes, never before detected as cycling, with high statistical fidelity. On the other hand, differences in gene lists between our meta-analyses reiterate the need for a multiplicity of cycling-gene detection algorithms. It seems that both types of analyses are necessary, complementing each other's biases and/or weaknesses to construct a more complete characterization of all cycling genes.

It is noteworthy to mention that while this manuscript was under preparation, detailed characterization [25–27] of one of our strong candidate genes (*CG17100*; one of our 133 novel genes)—pursued by this lab [26] in no small part due to the work presented here—verified it to be a bona fide member of the circadian clock, *cwo.* This adds substantive proof to the assertion that our newly discovered genes are strong candidates with respect to their potential circadian functions. These can serve as a focal point for future efforts to identify the remaining pathways of the fruit fly circadian clock.

### Conclusions

We introduce a novel analysis method that compensates for variability observed among the original five *Drosophila* circadian array reports. The principles of ANOVA-based prescreening and rigorous post hoc statistical testing we use are of benefit to our and two previously published techniques (the MacDonald algorithm and F24). We identify a set of cycling transcripts previously found in one or more of the original five reports as well as a novel set. Based on the statistical fidelity of our meta-analysis and the results of our pilot RT-PCR validation experiments (our assessment of expression rhythmicity was found to be consistent between array and RT-PCR data), we predict many of our newly found genes to be bona fide cyclers (e.g., *cwo*), and suggest that they may lead to new insights into the regulatory pathways through which clock mechanisms regulate behavioral rhythms.

## Methods

### Activity histogram.

To produce the behavioral activity histograms (Figure S1), the activity of *n* ∼ 100 flies of the indicated genotype was recorded via the Trikinetics *Drosophila* Activity Monitoring system (http://www.trikinetics.com). After 3–5 d of LD entrainment, data were recorded at 30-min bins for the last LD and first DD days. Data for every fly of each genotype were averaged together and presented as a histogram. Error bars indicate the standard error of the mean across all flies of the indicated genotype at the indicated time. An Excel-based macro (Keegan et al. [26]; available from the Allada lab upon request) was used to process behavioral data and produce the histogram figures.

### Data and algorithm collection.

Data for a few of the reports were accessible through public Web sites. To obtain data and algorithms not available through publicly accessible Web sites, we contacted authors. All data used to produce this report are available upon request. Files that contain the individually formatted results from each of the original reports were too numerous and large to be included with this manuscript on the *PLoS Computational Biology* Web site.

### Gene annotation.

In each table where our characterized genes are presented (Tables 4, S6, and S7), each gene is referred to by its Affymetrix probe set ID and, depending on availability from the Affymetrix *Drosophila* 1 microarray annotation database, the gene name, symbol or CG number, CT number, GenBank accession number (http://www.ncbi.nlm.nih.gov/genbank), and FlyBase accession number (http://flybase.bio.indiana.edu). Other gene lists refer to transcripts by their Affymetrix *Drosophila* Genome 1 microarray probe set. Complete annotation for these can be obtained free of charge from the Affymetrix Web site: (https://www.affymetrix.com/analysis/netaffx/index.affx).

### Comparison of original gene lists.

The original reports utilized a variety of methods to annotate transcripts, Affymetrix probe set numbers, CG number, CT number, gene ID, gene names and/or symbols, etc. We developed a Matlab-based code (MathWorks, http://www.mathworks.com) that could quickly provide the Affymetrix probe set number for any input found in the Affymetrix database (gene name, CG number, CT number, gene ID, gene symbol, etc.) such that each list could be represented with the same annotation. A second Matlab code was implemented to perform comparisons between any two lists of genes. It produces an output file that contains the list of genes found in common between any two input lists. Gene lists for this comparison were obtained directly from the primary literature [1,8–11]. All codes used to produce this report are available upon request.

### Data grouping.

As described in the text, we only used data prepared from *y w* and *CS* flies. Arrays prepared from *cn bw* flies were excluded from our analyses. Several reports contain multiple days worth of data. As our goal was to create a single-day, six-point-long time course, we grouped data from timepoints and whole-day multiples of the same timepoint together: ZT0 and ZT24 were counted as ZT0; CT0 and CT24 were counted as CT0. ZT, or *zeitgeber* (“time-giver”), time refers to 24-h-long days in the presence of an entraining stimulus, which in the case of an LD regime the stimulus is light; ZT0 corresponds to the time when lights turn on, ZT12 when they turn off. Similarly, CT or *circadian time* refers to 24-h–long days under constant conditions, which is complete darkness in the case of a DD light regime; CT0 corresponds to the time when the stimulus (light in this case) would have been expected to turn on, and CT12 corresponds the time it would have been turned off if it were present. We performed this grouping on LD data and DD data such that we were able to produce a six-point LD time course and six-point DD time course, each with *n* ≈ 10 arrays per timepoint. Most datasets followed a ZT or CT 0, 4, 8, 12, 16, 20 sampling regime. Two other sampling windows were used in the original reports, ZT/CT 1, 5, 9, 13, 17, 21 and ZT/CT 2, 6, 10, 14, 18, 22. We included data sampled at the ZT/CT 1, 5, 9, 13, 17, 21, but excluded data sampled at ZT/CT 2, 6, 10, 14, 18, 22. This allowed us to include the greatest possible number of arrays while controlling for expression differences that could be attributed to sampling intervals differentially phased by more than 1 h.

### Single-algorithm processing of individual datasets.

We applied the algorithm described in McDonald et al. [8] to the data from each of the five reports. As the McDonald algorithm was originally used on DD data, and our aim was to reproduce the original technique as closely as was possible, we applied it to DD data only; LD data were not considered in our McDonald algorithm analysis. First, data were grouped as indicated above (Data grouping). Then, DD data from each of the reports were treated exactly as described in McDonald et al. [8]. Briefly, expression data for all replicates of a given timepoint were averaged together, and a standard error was calculated. This produced a single six-point DD expression time course for each transcript. The average expression across all six timepoints had to exceed a mild filter (the averaged average-difference scores had to exceed a value of 20). The expression profile of each transcript that passed this filter was normalized according to the method reported in McDonald et al. [8]

Expression profiles then had to pass the following significance test:

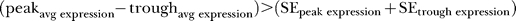
where *peak_avg expression_* and *trough_avg expression_* refer to the peak and trough expression values of a particular transcript expression profile, and *SE_peak expression_* and *SE_trough expression_* refer to the standard error of expression at the peak and trough points, respectively. A Matlab code was generated to automate this test. Profiles had to exhibit 1.5-fold differences between the peak and trough expression values. Last, a Perl script developed by Mike McDonald [8] was used to derive correlation scores between each transcript expression profile and several differentially phased sinusoidal waves with a period of 24 h. A correlation threshold score of 0.90 was used. We found reproduction of the McDonald algorithm to nearly, but not exactly reproduce the original results when it was applied to the McDonald dataset. Reproduction by McDonald produced a list identical to ours, slightly different than that published in the original report. (Independent reproduction of the technique by two parties [Keegan and McDonald] identified 136 probe sets, these include 132 of the originally reported 135 probe sets, and four probe sets not found in the originally published analysis; personal correspondence with Mike McDonald.) Comparison of lists of identified genes was performed using a Matlab code described above (see the section Comparison of original gene lists). Data processed with the McDonald algorithm were not standardized. Matlab- and Excel-based codes are available upon request. Perl scripts are available through Mike McDonald and/or Michael Rosbash [8].

### Multiple-algorithm processing of individual datasets.

The McDonald algorithm was applied to each of the datasets (to the raw, nonstandardized data) as described above. For each dataset, the output from the McDonald algorithm–based analysis was compared with the originally published output. Thus, for each report, we had the lists of transcripts identified under two different algorithmic techniques, the McDonald algorithm and whatever technique was native to the report. The lists of identified genes were compared using the Matlab code described above (see the section Comparison of original gene lists).

### Data transformation.

Expression values were transformed as described in Claridge-Chang et al. [1]: for each timepoint, the expression level relative to the mean (taken in log_2_ coordinates) over that experiment was computed*.* This procedure was separately performed on the LD and DD data expression values from each report. This led to the subsequent exclusion of any negative expression values (an artifact of MAS 4 and 5 expression condensation algorithms) from further analysis.

### Data standardization.

To achieve location and scale normalization among all arrays, we performed the following standardization procedure on each array after data were log transformed:

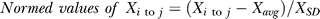
where *i* = 1, *j* = 14,010 (the number of transcripts probed by each array). *X_i_* is the expression value of the i^th^ transcript, *X_avg_* is the average expression of all expression measures *X_i_* to *X_j_*, and *X_SD_* is the standard deviation of expression for all expression measures *X_i_* to *X_j_* [17]. This normalization procedure sets the mean expression of each array to 0, and the variance to 1. Data from McDonald et al. [8] were standardized as described above, but as McDonald expression values represented an average calculated from three to five arrays, not the expression from individual arrays as was reported in each of the other studies, the standardized McDonald data were subsequently weighted such that each expression value was counted three times (the McDonald et al. [8] text specifies three to five replicates per timepoint, but does not indicate the exact number of replicates used for each timepoint; we used the most conservative estimate, three replicates for each timepoint). The standardized data were grouped as indicated above (see the section Data grouping) and used in all analyses described below.

### Production of averaged datasets.

After transformation and standardization, the remaining expression values were grouped according to LD or DD timepoint. When present, whole-day multiples of a single timepoint were grouped together (i.e., ZT4 and ZT28 were treated as ZT4). All expression measures within a given LD or DD timepoint were averaged together. The standard error of the mean for each timepoint was also determined.

### Production of appended datasets.

After transformation and standardization, the remaining expression values were grouped according to report and then LD or DD timepoint.

The data for each day in the multiday profiles (data for each single day came from a single report/lab) underwent one additional standardization procedures such that the data for each day would exhibit a mean of 0 and variance of 1.

This allowed for roughly equal weighting of each day in the expression profile; that is, expression values for each day were constrained such that their location and scale would be the same, but profile contours remained unchanged. In all correlation-based analyses, missing data (due to incomplete time series) and those data that failed to pass the log_2_ transformation were ignored. Under fast Fourier transform (F24) analysis, missing data (three timepoints in the LD dataset) and those data that failed to pass the log_2_ transformation were replaced with a value of 0.

### ANOVA analysis.

After grouping and standardization (described above), we had two datasets, a six-point LD time course with nine to ten arrays per timepoint, and a six-point DD time course with ten to 13 arrays per timepoint. Data from the LD and DD sets were treated separately. For each of the 14,010 transcripts on the arrays, a single-factor ANOVA was performed across all six timepoints. Each timepoint was treated as a group, and arrays at each timepoint were treated as the individuals within that group. We applied a fairly liberal selection criterion; a nonadjusted ANOVA *p*-value of 0.05 or less was required for any particular transcript to pass the screen. Data were grouped and sorted in Excel, then exported via tab-delimited text files to a Matlab code created to automate ANOVA processing. Our code utilized the embedded Matlab ANOVA function, and is available upon request.

### Correlation analysis.

*Construction of expression models.* As our primary interest was in identifying transcripts that exhibit cycling patterns, and not in finding the specific peak and trough expression timepoint(s) responsible for ANOVA results, we decided not to perform standard post hoc analyses. Instead, we used correlation techniques to identify transcripts that exhibited significant cycling. We developed three model waveforms. The first was a simple sinusoidal wave with a 24-h period. To account for the total range of possible phases, our model included 24 individual sin waves phased at hourly intervals to account for the total range of possible phases across a 24-h period. As our data took the form of six-point time courses, our models were also represented as six-point time courses. To accomplish this, we sampled each of the 24 sin waves at 4-h intervals, creating a single six-point representation for each of the 24 continuous sin waves. Thus, our sin model was composed of 24 differentially phased six-point sin wave profiles. Our second model was constructed from previously reported *per* expression data [21]. NIH image (http://rsb.info.nih.gov/nih-image/) was used to derive approximate numerical values of *per* mRNA expression from Figure 2A of So et al. [21], a Northern blot profile of *per* expression. We sampled this plot at 4-h intervals in 24 different phases to produce 24 differentially phased six-point *per* expression profiles. Our last model was similarly produced from behavior profiles of *CS* flies in LD or DD. The LD model was derived from the averaged LD behavior profile of *n* ≈ 100 flies (wild-type *CS*). As with the two previous models, the initial waveform was sampled at 4-h intervals in 24 phases. The DD model was created by application of an identical technique to an averaged DD behavior profile of *n* ≈ 100 flies (wild-type *CS*). The LD behavior-derived model was used to probe LD expression data, whereas the DD behavior-derived model was used to probe DD expression data. For correlation to appended data, the models described above were self-appended until the requisite profile length was achieved.

*Production of raw correlation scores and correlation p-values.* We used a Matlab code to perform Pearson correlation analyses between each transcript expression profile and our expression models. LD expression data were correlated with the sin expression, *per* expression, and LD behavior-based models. DD expression data were correlated to the sin expression, *per* expression, and the DD behavior based models. The code performed correlation between each transcript's expression profile and the selected expression models. When processing our averaged datasets, profiles were considered only if they possessed expression values for each of the six timepoints. Similarly, when processing the appended datasets, we required that each profile possess at least six data points. For incomplete appended profiles (those that had more than six but fewer than the total number of timepoints), missing data points were ignored. As each expression model contained 24 individual waveforms (one corresponding to each of the 24 hour-phased representations of the model; see the section Construction of expression models above), 24 correlations were performed for each transcript to model comparison. The code found the model phase that exhibited the highest absolute correlation score (accessed via the Pearson correlation coefficient) of these 24 correlations, recording the score and the model phase. To assess the significance of each correlation, we employed a permutation technique. First, correlation analysis was performed between a transcript and a given expression model, as described above. Then, the original transcript expression profile was randomized, and the highest absolute correlation score was recomputed and stored. This process was performed until all possible permutations of the averaged expression profile (six timepoints yield 720 possible arrangements of each average expression profile) or 10,000 randomly generated permutations of the appended profiles had been tested. Our correlation *p*-value was calculated as the number of permutation correlations that exhibited a higher correlation score than the real (nonpermuted) expression profile divided by the total number of permutations tested (720 or 10,000). This *p*-value can be interpreted as the probability that a correlation greater than that observed in the original (nonpermuted) profile could occur given any random arrangement of the expression values it contained. After the correlation score and correlation *p*-values were calculated by our Matlab code, we applied two filters to the output. (1) We required transcripts to exhibit a Pearson correlation score of at least 0.95. (2) To be deemed significant, correlations had to exhibit a permutation-derived *p*-value of 0.05 or less.

*Permutation-derived FDR calculations.* In addition to evaluating the significance of each individual correlation, we also wished to determine the FDR, the rate at which any positive result can be expected to be false, a measure of experiment-wide, as opposed to individual-test, significance [17]. We derived an FDR value for each of the expression models with a method similar to SAM [22]. First, to determine the number of positive results from the true data, we performed correlation analysis as indicated above, and determined the number of transcripts that passed all of our selection criteria. Next, to approximate the number of false positives, the expression profiles of all 14,010 transcripts was randomized. Correlation analysis was then performed on the randomized dataset. We scored transcripts that passed our selection criteria after randomization as false positives. Our FDR was then calculated using the following formula: (*number of false positives* / *number of original positives*).

*Power approximation.* To approximate the statistical power (essentially, a measure of how well any method could detect known cycling genes), we used a simple empirical procedure. We tested each method's ability to detect six well-known circadian genes *(Clk, cry, per, tim, vri,* and *Pdp1).* Our power statistic was calculated as *n*/6, where *n* was the number of the known cycling genes detected.

### Autocorrelation.

Whereas the standard application of autocorrelation would have required two or more days of data (a data profile at least two times the length of the period we were primarily interested in finding), we used a method that allowed for us to perform autocorrelation on a single day of data. Autocorrelation requires two representations of the same profile. The first representation (A) is held constant as the second (B) is shifted out of register with it one timepoint at a time; with each shift, data points at the extremities are “lost” as they are moved beyond the point where they can be paired with a point from the other representation. To avoid this loss of data, we considered each representation circularly. The point lost from the end of B after a single shift would be moved to B's beginning such that all points would be compared to all points at each registration (six timepoints at five nonidentical registrations for the six-point–long averaged time courses). In addition, we considered the absolute, rather than raw, value of the Pearson correlation coefficient. This allowed us to detect 24-h rhythmicity with a single day of data—two 24-h sin waves exactly 12 h out of phase with each other will produce an absolute Pearson correlation coefficient of 1. Autocorrelation was applied to averaged profiles only if they possessed expression values for each of the six timepoints. Autocorrelation was applied to appended profiles if they possessed six or more data points. The autocorrelation *p*-value was determined using the same procedure employed for determination of cross-correlation *p*-values. Our autocorrelation routine was implemented as a C++ script.

### F24 analysis.

Discrete Fourier transform (DFT)–based analysis, as nearly as we were able to reproduce the technique, was performed as described in the Wijnen studies [2,3]. Briefly, the F24 score represents the normalized spectral power determined for the 24-h period component in the expression profile. Under the Wijnen scaling of power, all values, regardless of expression profile length, will scale between 0 and 1, with 1 representing a perfectly sinusoidal signal. Thus, the power from profiles of different lengths can be directly compared [2,3]). The example of Matlab code below was produced from our best understanding of the sample code provided in Wijnen et al. [2]:

In the following example code, fast Fourier transform data are presented as a tab delimited text file imported as an array.

Each row is the expression for a single transcript, and each column is the expression for a single timepoint. Prior to implementation of the code, the data for each expression profile were standardized by subtraction of the mean and division by the standard deviation.

For *i* = 1:number_of_expression_profiles

transcript_i = FFT_data(i,:);

transcript_i _squared= transcript_i./sqrt(sum(transcript_i. ^2));

y=fft(transcript_i _squared);

z=y.*conj(y);

fft_power=z/length(transcript_i _squared)*2;

end;

where *fft* refers to the DFT; as stated in the Matlab help file for this function, *fft(X)* is the DFT of vector *X*. For length *N* input vector *x*, the DFT is a length *N* vector *X*, with elements:

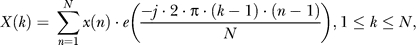



The portion of our code presented above simply performs the power calculations: our complete code produces a labeled output file with power and power *p*-values for all determinable periods. The complete code is available upon request to the authors.

### Clustering.

Clustering was performed as has previously been described [23]. We used freely available software to produce the cluster images [24]. Standardized expression values were averaged together for each timepoint to produce a single six-point LD time course for each gene. The standardized, averaged expression values for each transcript were then normalized such that all transcripts exhibited an expression range from 0 to 1. The Pearson correlation coefficient was selected as our distance metric.

### Quantitative RT-PCR.

We performed three separate experiments. Flies were entrained in an LD environment for 3–6 d and then collected at 4-h intervals through a single day. RNA was isolated from fly heads using the Invitrogen Trizol reagent [39]. DNAse I treatment of isolated nucleic acid was utilized to eliminate DNA contamination of samples. SYBR green reagent was used along with the following oligos to perform a real time PCR analysis of mRNA levels. Expression levels were quantified using an ABI 7900HT along with Sequence Detection System software (Applied Biosystems, http://www.appliedbiosystems.com). Expression profiles were assessed using all four of our cross-correlation methods, autocorrelation, and F24. Table S5 contains sequence data for all oligos used to perform our RT-PCRs. RT-PCR data for *CG17100* (*cwo*) were adapted from Lim et al. [26].

## Supporting Information

Dataset S1 Addendum to Table 1
This addendum to Table 1 contains the lists referred to in that table.
(549 KB XLS)

Dataset S2 Addendum to Table 4
This addendum to Table 4 contains a summary of all analyses performed on all transcripts in the compiled datasets (LD data in addendum A, DD data in addendum B). Data are split between transcripts that passed our ANOVA prescreen, and those that did not.
(15.2 MB XLS)

Dataset S3 Interactive Addendum to Figure 3
This interactive Excel worksheet (instructions on use are included in the file) allows the user to examine and print the averaged LD data (with calculated standard error) for all 14,010 transcripts examined in this study. Transcripts are identified by their Affymetrix probe set, and sorted according to the post hoc test (if any) by which they were identified as cycling.
(4.5MB XLS).

Figure S1 Behavior of Wild-Type Strains This Word document presents the data we used to screen strains with respect to their circadian behavior.
(30 KB DOC)

Figure S2 Distribution of Expression Values at Each Preprocessing Stage These Powerpoint files display the distributions of expression values (LD in [A], DD in [B]) of data from the individual and compiled datasets at each stage of our preprocessing procedure.
(150 KB DOC)

Figure S3 Cross-correlation Model Waveforms This Word file displays the waveforms used to construct the expression models utilized in our cross-correlation post hoc tests.
(97 KB DOC)

Figure S4 Simple FDR Optimization for Our Application of the F24 Method to the Meta-Dataset This Excel worksheet contains the results for the FDR optimization of our implementation of F24.
(21 KB DOC)

Table S1 Summary of Original Methods Table S1 summarizes the various methods used in the original five reports to identify cycling transcripts.
(65 KB DOC)

Table S2 Overlap among Original Reports Table S2 indicates the overlap found among transcripts identified in the original five reports.
(278 KB DOC)

Table S3 Comparison of Multiple-Algorithm Output of the Same Dataset Differences in identified transcripts present when each dataset is processed with two different algorithms.
(95 KB DOC)

Table S4 Overlap among Reports Using a Single Algorithm Overlap present among all datasets when each is processed with the same algorithmic technique.
(91 KB DOC)

Table S5 Oligos Used for RT-PCR (13 KB DOC)

Table S6 Originally Discovered Genes Found in Common with the LD Meta-Dataset Comparison between the LD meta-dataset and the list of transcripts identified in each of the original reports. It contains the number and identity (as Affymetrix probe set ID) of all transcripts found in common between the LD meta-dataset and each original set.
(152 KB DOC)

Table S7 ANOVA Significant Probe Sets All genes identified by our ANOVA screen (LD and DD datasets) possessing significant changes in expression over time. Listed are all probe sets that passed our initial ANOVA screen (sets exhibit a nonadjusted ANOVA *p*-value of 0.05 or less). ANOVA values were calulate using a Matlab script; see Methods for details
(1.8 MB DOC)

## Abbreviations

DD: constant darkness
DFT: discrete Fourier transform
FDR: false discovery rate
LD: light–dark
RMA: robust multiarray average
