# Supplementary material for: Meta-Analysis of Drosophila Circadian Microarray Studies Identifies a Novel Set of Rhythmically Expressed Genes
Source: PLoS Comput Biol. 2007 Nov 2;3(11):e208. doi: 10.1371/journal.pcbi.0030208 (PMC2098839; doi:10.1371/journal.pcbi.0030208)
Supplement: Figure S1 — This Word document presents the data we used to screen strains with respect to their circadian behavior. (30 KB DOC) [file pcbi.0030208.sg001.doc]

# Supplemental
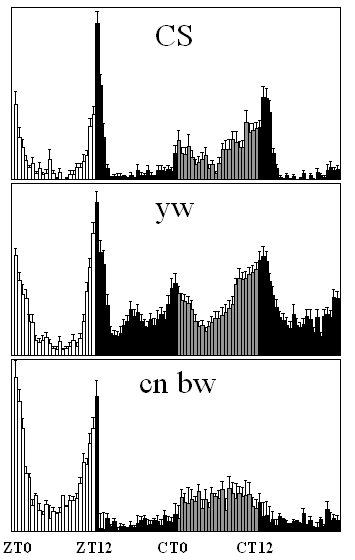
Figure 1. Behavior of wild type strains

Averaged behavior for flies is shown for three genotypes through the last day of LD (light/black bars) and the first day in DD (gray/black bars).

Each bar represents the average activity of n ≈ 100 flies for one half hour. Error bars indicate the standard error of the mean.
