# Supplementary material for: Meta-Analysis of Drosophila Circadian Microarray Studies Identifies a Novel Set of Rhythmically Expressed Genes
Source: PLoS Comput Biol. 2007 Nov 2;3(11):e208. doi: 10.1371/journal.pcbi.0030208 (PMC2098839; doi:10.1371/journal.pcbi.0030208)
Supplement: Figure S2 — These Powerpoint files display the distributions of expression values (LD in [A], DD in [B]) of data from the individual and compiled datasets at each stage of our preprocessing procedure. (150 KB DOC) [file pcbi.0030208.sg002.pdf]

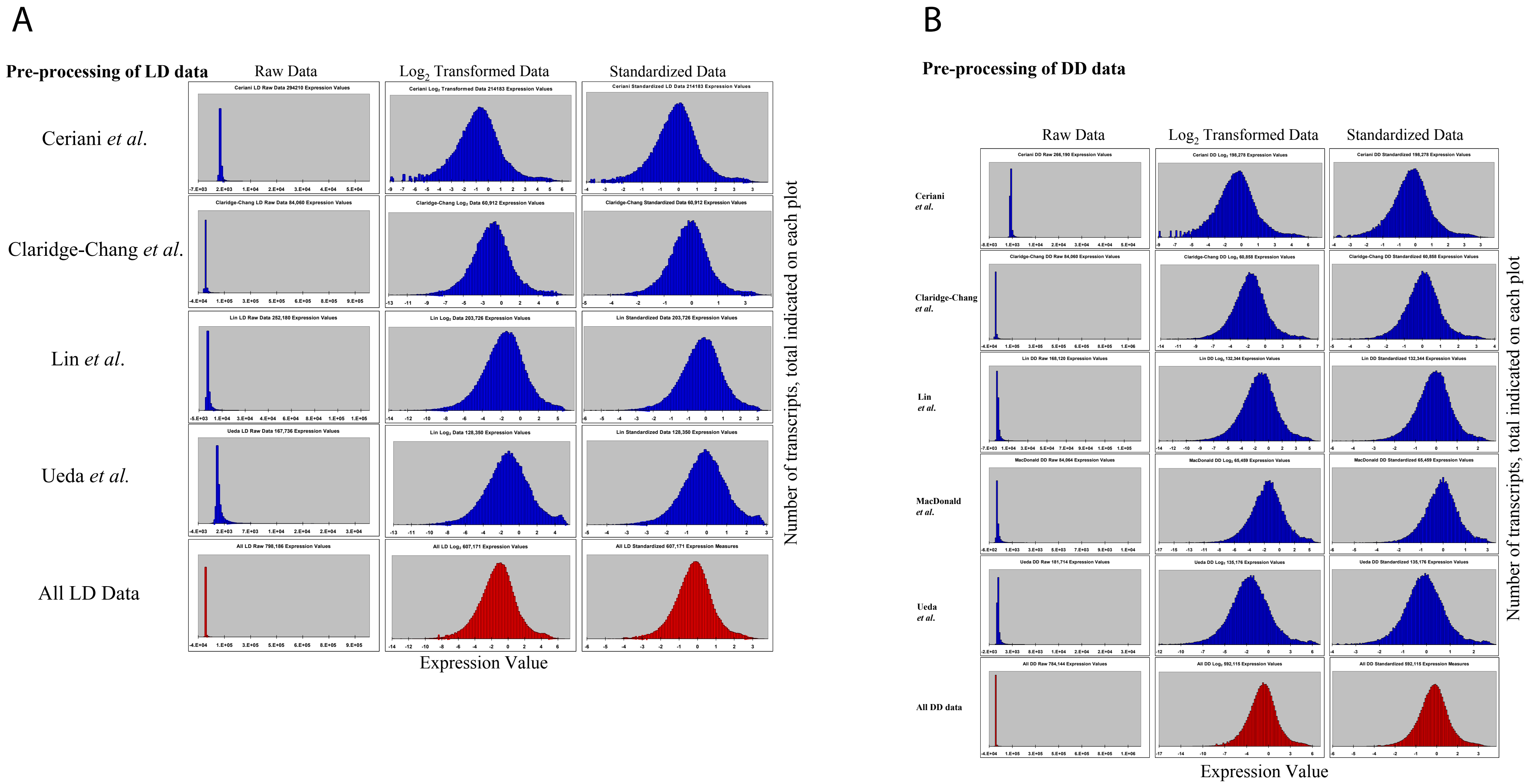
A
Pre-processing of LD data
Raw Data
Log2 Transformed Data
Standardized Data
Ceriani et al.
Claridge-Chang et al.
Lin et al.
Ueda et al.
All LD Data
Expression Value
Number of transcripts, total indicated on each plot
B
Pre-processing of DD data
Raw Data
Log2 Transformed Data
Standardized Data
Ceriani et al.
Claridge-Chang et al.
Lin et al.
MacDonald et al.
Ueda et al.
All DD data
Expression Value
Number of transcripts, total indicated on each plot
