# Supplementary material for: Meta-Analysis of Drosophila Circadian Microarray Studies Identifies a Novel Set of Rhythmically Expressed Genes
Source: PLoS Comput Biol. 2007 Nov 2;3(11):e208. doi: 10.1371/journal.pcbi.0030208 (PMC2098839; doi:10.1371/journal.pcbi.0030208)
Supplement: Figure S3 — This Word file displays the waveforms used to construct the expression models utilized in our cross-correlation post hoc tests. (97 KB DOC) [file pcbi.0030208.sg003.doc]

**Sin**

0

5

10

15

20

**So 97 Per Expression**

0

5

10

15

20

**LD Behavior**

1

6

11

16

21

**DD Behavior**

1

6

11

16

21

**So 97 Per Expression**

0

5

10

15

20

Cross Correlation Model Waveforms

Supplemental Figure 3. Cross-Correlation Model Waveforms

A. The four model waveforms used in our cross-correlation analyses are presented. Each waveform spans a 24 hour period. The displayed models were sampled at a four-hour interval in 24 hour-differentiated phases. These hour-phased profiles were correlated to each of the 14010 probe set expression profiles. For correlations to the appended data set, the model waveform was appended to itself to achieve the requisite length and then sampled as described. See Methods for details.

B. Per expression as accessed via three separate techniques, northern blot (So *et al*. 1997) , averaged LD microarray data considered in this study, averaged RT-PCR results.

A

B

Northern Blot Averaged LD Microarray Averaged RT-PCR
