# Supplementary material for: Meta-Analysis of Drosophila Circadian Microarray Studies Identifies a Novel Set of Rhythmically Expressed Genes
Source: PLoS Comput Biol. 2007 Nov 2;3(11):e208. doi: 10.1371/journal.pcbi.0030208 (PMC2098839; doi:10.1371/journal.pcbi.0030208)
Supplement: Table S1 — summarizes the various methods used in the original five reports to identify cycling transcripts. (65 KB DOC) [file pcbi.0030208.st001.doc]

###### Supplemental Table 1. Summary of Original Methods

Table 1 summarizes the analysis methods used in the five original reports. NA (not applicable) refers to procedures not performed in the indicated report(s).

**Pre-screening** refers to data culling processes performed before cyclic expression detection algorithms were applied to the indicated author’s data set. *Threshold* indicates that an absolute or fold change based expression level threshold was used to cull genes.

**Early processing** refers to procedures employed to scan and preliminarily process arrays. *Affymetrix* indicates that proprietary Affymetrix software was used to produce “*.DAT” and “*.CEL” level files.

**Expression** refers to the software package used to derive expression measures for each probe set from “*.CEL” files.

**Correlation method** indicates the general type of correlation performed. Each of the original reports utilized at least one such procedure to identify cyclic transcripts.

**Other methods** refers to methods other than the indicated correlation techniques utilized to identify cycling transcripts.

**Significance test** indicates the type of significance test employed to evaluate correlation results.

**False + test** and **False – test** indicate the general method by which the false positive and false negative discovery rates were determined.
