# Supplementary material for: Meta-Analysis of Drosophila Circadian Microarray Studies Identifies a Novel Set of Rhythmically Expressed Genes
Source: PLoS Comput Biol. 2007 Nov 2;3(11):e208. doi: 10.1371/journal.pcbi.0030208 (PMC2098839; doi:10.1371/journal.pcbi.0030208)
Supplement: Table S3 — Differences in identified transcripts present when each dataset is processed with two different algorithms. (95 KB DOC) [file pcbi.0030208.st003.doc]

Supplemental Table 3. Comparison of Multiple Algorithm Output of the Same Data Set

Column headings indicate the author of the analyzed data set. For each data set, the number of transcripts identified under the native and McDonald algorithms are indicated. In the bottom row, solid values indicate the number, and in parenthesis the percent, of total possible matches between the original and McDonald derived lists of cycling transcripts. After the colon, italicized values indicate the number, and in parentheses the percent, of total possible matches expected given any two lists of randomly selected genes equal in length to those produced by the two analyses.
