# Supplementary material for: Meta-Analysis of Drosophila Circadian Microarray Studies Identifies a Novel Set of Rhythmically Expressed Genes
Source: PLoS Comput Biol. 2007 Nov 2;3(11):e208. doi: 10.1371/journal.pcbi.0030208 (PMC2098839; doi:10.1371/journal.pcbi.0030208)
Supplement: Table S4 — Overlap present among all datasets when each is processed with the same algorithmic technique. (91 KB DOC) [file pcbi.0030208.st004.doc]

Supplemental Table 4. Overlap Among Reports Using a Single Algorithm

Results indicate the number of transcripts identified in common under McDonald algorithm processing of each data set.

**A.** Numbers next to a bracketed author name indicate the number of cycling transcripts identified by that author. For each between report comparison, four values are presented. The first and second indicate the number of matches found in common between the two reports, expressed first as a raw sum, and in parentheses as the percent of the total possible matches achieved. The third and fourth values (italicized, after colon) indicate the number of matches expected by chance, first as a raw sum and, in parentheses as the percent of total possible matches achieved.

**B.** The first entry indicates the range of transcripts identified in each of the five reports. The second through fifth entries each contain four values. The first two indicate the number and, in parenthesis, the percent of total possible matches achieved by the indicated comparison. Italicized values indicate the number and, in parenthesis, the percent of total possible matches expected by chance given randomly selected lists of genes equal in length to those reported among the five studies.
