# Supplementary material for: Meta-Analysis of Drosophila Circadian Microarray Studies Identifies a Novel Set of Rhythmically Expressed Genes
Source: PLoS Comput Biol. 2007 Nov 2;3(11):e208. doi: 10.1371/journal.pcbi.0030208 (PMC2098839; doi:10.1371/journal.pcbi.0030208)
Supplement: Table S7 — All genes identified by our ANOVA screen (LD and DD datasets) possessing significant changes in expression over time. Listed are all probe sets that passed our initial ANOVA screen (sets exhibit a nonadjusted ANOVA p-value of 0.05 or less). ANOVA values were calulate using a Matlab script; see Methods for details (1.8 MB DOC) [file pcbi.0030208.st007.doc]

**Table 1.** ANOVA Significant Probe Sets

| LD Data or DD Data |  | Affymetrix Probe Set | ANOVA F Stat | ANOVA *p*-Value | CG Number or Gene Symbol | CT Number | GenBank Accession Number | FlyBase Accession Number |
| --- | --- | --- | --- | --- | --- | --- | --- | --- |
| **LD data (372)** | 1 | 141203_at | 2.4678 | 0.044585 | *CG2947* | CT9995 | AE003429 | FBgn0029676 |
|  | 2 | 141237_at | 5.1027 | 0.000721 | *CG9705* | CT27440 | AE003526 | FBgn0036661 |
|  | 3 | 141248_at | 3.6774 | 0.006438 | *CG5031* | CT16138 | AE003533 | FBgn0036435 |
|  | 4 | 141277_at | 2.7414 | 0.029444 | *CG3281* | CT10955 | AE003693 | FBgn0037967 |
|  | 5 | 141410_at | 3.1543 | 0.014954 | *CG9307* | CT26495 | AE003701 | FBgn0038180 |
|  | 6 | 141447_at | 4.2541 | 0.002724 | *CG4914* | CT15764 | AE003533 | FBgn0036436 |
|  | 7 | 141512_at | 2.891 | 0.022561 | *CG10355* | CT29092 | AE003663 | FBgn0032802 |
|  | 8 | 141522_at | 7.5826 | 2.30E-05 | *CG8643* | CT8739 | AE003836 | FBgn0033311 |
|  | 9 | 141541_at | 2.7879 | 0.026628 | *CG13868* | CT33390 | AE003792 | FBgn0034501 |
|  | 10 | 141543_at | 3.06 | 0.0172 | *l(2)not* | CT13514 | AE003461 | FBgn0011297 |
|  | 11 | 141593_at | 25.4483 | 8.91E-13 | *CG5798* | CT18196 | AE003733 | FBgn0038862 |
|  | 12 | 141613_at | 3.364 | 0.019194 | *CG2226* | CT7392 | AE003838 | FBgn0033262 |
|  | 13 | 141646_at | 5.1999 | 0.000623 | *CG17544* | CT38761 | AE003662 | FBgn0032775 |
|  | 14 | 141653_at | 2.8312 | 0.024836 | *EG:22E5.3* | CT13484 | AE003423 | FBgn0025630 |
|  | 15 | 141658_at | 2.3981 | 0.049883 | *CG5853* | CT18369 | AE003626 | FBgn0032167 |
|  | 16 | 141732_at | 3.3151 | 0.011438 | *puc* | CT23760 | AE003677 | FBgn0004210 |
|  | 17 | 141762_at | 3.0163 | 0.018448 | *Cp1* | CT20780 | AE003816 | FBgn0013770 |
|  | 18 | 141795_at | 2.7738 | 0.027714 | *CG13644* | CT33039 | AE003749 | FBgn0039247 |
|  | 19 | 141838_at | 3.3833 | 0.047732 | *CG15642 CG9201* |  |  | FBgn0030644 |
|  | 20 | 141856_at | 2.9877 | 0.027677 | *CG11577 CG10424* |  |  | FBgn0036848 |
|  | 21 | 141869_at | 9.0276 | 0.027851 | *Ugt35b* |  |  | FBgn0037867 |
|  | 22 | 141884_at | 3.2685 | 0.028358 | *antdh* |  |  | FBgn0030277 |
|  | 23 | 141954_at | 7.5213 | 0.024102 | *CG8600 CG8598* |  |  | FBgn0035766 |
|  | 24 | 142022_at | 2.8263 | 0.036193 | *CG5091 CG5096* |  |  | FBgn0032235 |
|  | 25 | 142057_at | 2.681 | 0.032412 | *CG13650 CG10057* |  |  | FBgn0039278 |
|  | 26 | 142092_at | 97450.9902 | 0.002355 | *alpha-Est3* |  |  | FBgn0015571 |
|  | 27 | 142104_at | 2.8006 | 0.038427 | *Sop2* |  |  | FBgn0004406 |
|  | 28 | 142154_at | 86.001 | 0 | *tim* | CT39082 | AE003579 | FBgn0014396 |
|  | 29 | 142175_at | 5.9951 | 0.001207 | *CG13013* | CT32225 | AE003503 | FBgn0040876 |
|  | 30 | 142201_at | 2.4767 | 0.048436 | *CG4363* | CT14230 | AE003455 | FBgn0034663 |
|  | 31 | 142208_at | 3.157 | 0.024099 | *CG5762* | CT18086 | AE003747 | FBgn0039190 |
|  | 32 | 142258_at | 2.6945 | 0.046584 | *CG12766* | CT30409 | AE003479 | FBgn0035476 |
|  | 33 | 142271_at | 16.8886 | 8.08E-10 | *Ugt35b* | CT20678 | AE003690 | FBgn0026314 |
|  | 34 | 142276_at | 2.7405 | 0.029485 | *zfh2* | CT3397 | AE003843 | FBgn0004607 |
|  | 35 | 142289_i_at | 4.6318 | 0.00935 | *CG13600* | CT32985 | AE003745 | FBgn0039134 |
|  | 36 | 142293_at | 2.5606 | 0.038398 | *CG12286* | CT19021 | AE003696 | FBgn0038077 |
|  | 37 | 142371_at | 6.1065 | 0.001372 | *NaPi-T* | CT28711 | AE003813 | FBgn0016684 |
|  | 38 | 142515_at | 2.5799 | 0.049454 | *fau* | CT41978 | AE003688 | FBgn0020439 |
|  | 39 | 142542_at | 3.6124 | 0.010555 | *CG2841* | CT9712 | AE003423 | FBgn0029614 |
|  | 40 | 142626_at | 10.766 | 0.006644 | *CG13906* | CT33443 | AE003469 | FBgn0035177 |
|  | 41 | 142682_at | 3.4401 | 0.012128 | *Nach* | CT24238 | AE003806 | FBgn0024319 |
|  | 42 | 142708_at | 2.9178 | 0.022729 | *CG4980* | CT15977 | AE003763 | FBgn0039558 |
|  | 43 | 142735_at | 3.5063 | 0.00844 | *CG7047* | CT42521 | AE003467 | FBgn0035103 |
|  | 44 | 142741_at | 3.6522 | 0.011413 | *CG15901* | CT34188 | AE003803 | FBgn0034211 |
|  | 45 | 142930_at | 3.0475 | 0.025515 | *CG7737* | CT23553 | AE003826 | FBgn0033584 |
|  | 46 | 142989_at | 3.0911 | 0.016364 | *CG8008* | CT8032 | AE003834 | FBgn0033387 |
|  | 47 | 142998_at | 767.4619 | 0.025516 | *CG17649* | CT38941 | AE003585 | FBgn0031355 |
|  | 48 | 143066_at | 16.4886 | 0.022095 | *amd* | CT29468 | AE003661 | FBgn0000075 |
|  | 49 | 143130_at | 3.873 | 0.004733 | *Pka-C3* | CT19108 | AE003529 | FBgn0000489 |
|  | 50 | 143154_at | 2.7945 | 0.03329 | *eyg* | CT29422 | AE003541 | FBgn0000625 |
|  | 51 | 143184_at | 6.1017 | 0.000168 | *GstD1* | CT28269 | AE003695 | FBgn0001149 |
|  | 52 | 143201_at | 2.4589 | 0.049767 | *ImpL3* | CT28577 | AE003563 | FBgn0001258 |
|  | 53 | 143203_at | 5.6841 | 0.000306 | *inaD* | CT11813 | AE003458 | FBgn0001263 |
|  | 54 | 143220_at | 5.4732 | 0.006288 | *wor* | CT13175 | AE003647 | FBgn0001983 |
|  | 55 | 143236_at | 2.9312 | 0.023344 | *lds* | CT9083 | AE003676 | FBgn0002542 |
|  | 56 | 143238_at | 2.4533 | 0.046561 | *Lsp1alpha* | CT8649 | AE003489 | FBgn0002562 |
|  | 57 | 143300_at | 9.0719 | 4.33E-06 | *per* | CT8963 | AE003425 | FBgn0003068 |
|  | 58 | 143337_at | 2.5941 | 0.038509 | *sd* | CT24895 | AE003500 | FBgn0003345 |
|  | 59 | 143355_at | 2.7595 | 0.027876 | *sn* | CT3977 | AE003442 | FBgn0003447 |
|  | 60 | 143356_at | 4.3432 | 0.027402 | *sna* | CT13146 | AE003647 | FBgn0003448 |
|  | 61 | 143390_at | 4.3798 | 0.002154 | *betaTub56D* | CT37566 | AE003795 | FBgn0003887 |
|  | 62 | 143445_at | 7.7805 | 0.013368 | *Wnt2* | CT5942 | AE003833 | FBgn0004360 |
|  | 63 | 143468_at | 3.1227 | 0.028006 | *LysP* | CT8825 | AE003470 | FBgn0004429 |
|  | 64 | 143475_at | 2.9173 | 0.022046 | *dy* | CT26577 | AE003487 | FBgn0004511 |
|  | 65 | 143501_at | 2.5403 | 0.040856 | *hh* | CT14986 | AE003742 | FBgn0004644 |
|  | 66 | 143528_at | 2.4175 | 0.048349 | *plx* | CT1567 | AE003602 | FBgn0004879 |
|  | 67 | 143537_at | 5.0826 | 0.000742 | *gol* | CT9061 | AE003466 | FBgn0004919 |
|  | 68 | 143546_at | 2.6355 | 0.034294 | *Shal* | CT26372 | AE003516 | FBgn0005564 |
|  | 69 | 143554_at | 11.1443 | 2.75E-07 | *trpl* | CT4790 | AE003832 | FBgn0005614 |
|  | 70 | 143559_at | 6.5971 | 0.000176 | *ple* | CT28477 | AE003561 | FBgn0005626 |
|  | 71 | 143581_at | 205.9981 | 0.004835 | *Msp-300* | CT41346 | AE003608 | FBgn0010070 |
|  | 72 | 143606_at | 5.9781 | 0.0002 | *Cyp18a1* | CT20826 | AE003509 | FBgn0010383 |
|  | 73 | 143647_at | 2.6578 | 0.033087 | *Pbprp3* | CT31758 | AE003601 | FBgn0011281 |
|  | 74 | 143648_at | 4.5279 | 0.010249 | *Pbprp4* | CT2057 | AE003672 | FBgn0011282 |
|  | 75 | 143651_at | 3.0149 | 0.028151 | *Taf30beta* | CT13550 | AE003626 | FBgn0011291 |
|  | 76 | 143707_at | 3.1339 | 0.03001 | *Odc2* | CT9690 | AE003839 | FBgn0013308 |
|  | 77 | 143717_at | 2.6496 | 0.037237 | *Awh* | CT1345 | AE003479 | FBgn0013751 |
|  | 78 | 143847_at | 3.8481 | 0.008515 | *rtGEF* | CT28279 | AE003666 | FBgn0015803 |
|  | 79 | 143869_at | 54.1925 | 0 | *vri* | CT33588 | AE003609 | FBgn0016076 |
|  | 80 | 143905_at | 3.9146 | 0.004435 | *Rh6* | CT16621 | AE003709 | FBgn0019940 |
|  | 81 | 143970_at | 7.8694 | 1.58E-05 | *Clk* | CT13692 | AE003557 | FBgn0023076 |
|  | 82 | 144067_at | 2.582 | 0.0371 | *CG4101* | CT13608 | AE003526 | FBgn0025558 |
|  | 83 | 144121_at | 7.1025 | 0.008125 | *C3G* | CT10408 | AE003438 | FBgn0026145 |
|  | 84 | 144123_at | 4.2354 | 0.029426 | *SAK* | CT22193 | AE003594 | FBgn0026371 |
|  | 85 | 144146_at | 3.2739 | 0.012216 | *Eaat1* | CT12517 | AE003624 | FBgn0026439 |
|  | 86 | 144149_at | 2.4115 | 0.048815 | *Ady43A* | CT5683 | AE003842 | FBgn0026602 |
|  | 87 | 144153_at | 2.6578 | 0.033086 | *fu12* | CT32749 | AE003621 | FBgn0026718 |
|  | 88 | 144166_at | 3.5767 | 0.024993 | *EG:33C11.3* | CT12157 | AE003420 | FBgn0026875 |
|  | 89 | 144269_at | 4.264 | 0.028637 | *BG:DS07486.2* | CT30353 | AE003649 | FBgn0028858 |
|  | 90 | 144278_at | 2.7427 | 0.03054 | *BG:DS06874.2* | CT15163 | AE003645 | FBgn0028869 |
|  | 91 | 144318_at | 3.2097 | 0.023317 | *BG:DS00365.2* | CT40635 | AE003649 | FBgn0028934 |
|  | 92 | 144338_at | 4.4984 | 0.001796 | *CG13377* | CT32709 | AE003417 | FBgn0029517 |
|  | 93 | 144350_at | 3.2708 | 0.012424 | *CG5254* | CT16777 | AE003418 | FBgn0029533 |
|  | 94 | 144389_at | 3.585 | 0.007672 | *CG14811* | CT34624 | AE003422 | FBgn0029590 |
|  | 95 | 144395_at | 3.2978 | 0.013563 | *CG14053* | CT33614 | AE003423 | FBgn0029603 |
|  | 96 | 144417_at | 3.0612 | 0.02915 | *CG14417* | CT34074 | AE003425 | FBgn0029637 |
|  | 97 | 144474_at | 4.4511 | 0.018352 | *CG12692* | CT35696 | AE003430 | FBgn0029703 |
|  | 98 | 144572_at | 2.426 | 0.047994 | *CG4666* | CT15013 | AE003436 | FBgn0029838 |
|  | 99 | 144593_at | 284.2904 | 0.044996 | *CG15894* | CT34111 | AE003437 | FBgn0029864 |
|  | 100 | 144633_at | 12.7218 | 0.031149 | *CG14431* | CT34093 | AE003439 | FBgn0029922 |
|  | 101 | 144636_at | 3.1844 | 0.025957 | *CG3032* | CT9431 | AE003439 | FBgn0029928 |
|  | 102 | 144647_at | 2.8437 | 0.036296 | *CG15034* | CT34897 | AE003440 | FBgn0029946 |
|  | 103 | 144739_at | 3.8376 | 0.005174 | *CG12120* | CT7356 | AE003446 | FBgn0030106 |
|  | 104 | 144747_at | 2.6171 | 0.035319 | *CG15910* | CT35394 | AE003446 | FBgn0030117 |
|  | 105 | 144778_at | 4.4867 | 0.00217 | *CG12649* | CT35297 | AE003449 | FBgn0030169 |
|  | 106 | 144815_at | 4.9537 | 0.041469 | *CG9817* | CT27740 | AE003451 | FBgn0030219 |
|  | 107 | 144945_at | 2.8645 | 0.023982 | *CG15729* | CT35962 | AE003489 | FBgn0030399 |
|  | 108 | 144985_at | 5.1432 | 0.034382 | *CG4349* | CT14212 | AE003491 | FBgn0030449 |
|  | 109 | 145007_at | 5.6422 | 0.016069 | *CG12725* | CT36000 | AE003492 | FBgn0030483 |
|  | 110 | 145025_at | 5.3669 | 0.000554 | *CG12177* | CT9165 | AE003493 | FBgn0030510 |
|  | 111 | 145111_at | 4.9404 | 0.001664 | *CG9101* | CT26120 | AE003498 | FBgn0030622 |
|  | 112 | 145125_at | 3.1406 | 0.031168 | *CG6324* | CT19774 | AE003499 | FBgn0030647 |
|  | 113 | 145136_at | 4.2764 | 0.012856 | *CG8119* | CT24274 | AE003499 | FBgn0030664 |
|  | 114 | 145156_at | 5.1119 | 0.013836 | *CG8565* | CT24957 | AE003500 | FBgn0030697 |
|  | 115 | 145206_at | 5.9873 | 0.000205 | *CG18358* | CT41728 | AE003503 | FBgn0030782 |
|  | 116 | 145270_at | 2.6909 | 0.032784 | *CG6847* | CT21205 | AE003507 | FBgn0030884 |
|  | 117 | 145278_at | 146.5332 | 0.006786 | *CG12612* | CT34934 | AE003507 | FBgn0030896 |
|  | 118 | 145326_s_at | 3.7341 | 0.005981 | *CG18259* | CT41379 | AE003510 | FBgn0030956 |
|  | 119 | 145348_at | 3.5027 | 0.008728 | *CG7531* | CT23069 | AE003511 | FBgn0030988 |
|  | 120 | 145361_at | 3.1856 | 0.024934 | *CG7890* | CT23770 | AE003511 | FBgn0031005 |
|  | 121 | 145424_at | 4.5321 | 0.020316 | *CG11748* | CT35531 | AE003571 | FBgn0031109 |
|  | 122 | 145477_at | 2.7299 | 0.032186 | *CG12564* | CT34320 | AE003568 | FBgn0031179 |
|  | 123 | 145493_at | 2.7198 | 0.042777 | *CG9557* | CT17180 | AE003574 | FBgn0031199 |
|  | 124 | 145515_at | 2.5466 | 0.039836 | *CG3709* | CT12433 | AE003590 | FBgn0031227 |
|  | 125 | 145570_at | 19.6983 | 7.05E-11 | *CG5156* | CT16503 | AE003587 | FBgn0031326 |
|  | 126 | 145629_at | 3.0013 | 0.032555 | *CG4270* | CT11353 | AE003583 | FBgn0031407 |
|  | 127 | 145880_at | 5.2614 | 0.015544 | *CG9481* | CT26858 | AE003613 | FBgn0031790 |
|  | 128 | 145926_at | 3.265 | 0.012391 | *CG17378* | CT33256 | AE003615 | FBgn0031858 |
|  | 129 | 145937_at | 4.135 | 0.007516 | *CG13776* | CT33264 | AE003615 | FBgn0031880 |
|  | 130 | 145966_at | 4976.0039 | 0.010024 | *CG6739* | CT20919 | AE003618 | FBgn0031926 |
|  | 131 | 145976_s_at | 3.9592 | 0.007088 | *CG18590* | CT42499 | AE003618 | FBgn0031938 |
|  | 132 | 146012_at | 13.1195 | 3.19E-08 | *CG8419* | CT24715 | AE003620 | FBgn0031999 |
|  | 133 | 146022_at | 14.4966 | 7.84E-09 | *CG14275* | CT33900 | AE003620 | FBgn0032022 |
|  | 134 | 146054_at | 4.0736 | 0.006641 | *CG9525* | CT26950 | AE003623 | FBgn0032080 |
|  | 135 | 146088_at | 3.0312 | 0.019024 | *CG3769* | CT12594 | AE003624 | FBgn0032119 |
|  | 136 | 146115_at | 4.0262 | 0.029107 | *CG5831* | CT18295 | AE003627 | FBgn0032173 |
|  | 137 | 146129_at | 3.0679 | 0.02013 | *CG5731* | CT18017 | AE003627 | FBgn0032192 |
|  | 138 | 146167_at | 3.2261 | 0.013184 | *CG18302* | CT41543 | AE003629 | FBgn0032266 |
|  | 139 | 146172_at | 3.3563 | 0.036406 | *CG7329* | CT22593 | AE003629 | FBgn0032271 |
|  | 140 | 146180_at | 2.8574 | 0.024028 | *CG17107* | CT33651 | AE003629 | FBgn0032281 |
|  | 141 | 146182_at | 3.8213 | 0.005133 | *CG7296* | CT22519 | AE003629 | FBgn0032283 |
|  | 142 | 146214_at | 3.5355 | 0.008058 | *CG16743* | CT34737 | AE003630 | FBgn0032322 |
|  | 143 | 146226_at | 2.4159 | 0.048776 | *CG14920* | CT34747 | AE003631 | FBgn0032337 |
|  | 144 | 146233_at | 2.6353 | 0.034306 | *CG4779* | CT15337 | AE003631 | FBgn0032349 |
|  | 145 | 146274_at | 3.1897 | 0.013974 | *CG17211* | CT34781 | AE003635 | FBgn0032414 |
|  | 146 | 146306_at | 3.9818 | 0.026247 | *CG5075* | CT16217 | AE003638 | FBgn0032464 |
|  | 147 | 146318_at | 3.2603 | 0.034358 | *CG15480* | CT35574 | AE003639 | FBgn0032489 |
|  | 148 | 146346_at | 223.9201 | 0.004451 | *CG16888* | CT35255 | AE003641 | FBgn0032533 |
|  | 149 | 146392_at | 2.4625 | 0.045259 | *CG4855* | CT15595 | AE003653 | FBgn0032623 |
|  | 150 | 146435_at | 3.8776 | 0.007116 | *CG10174* | CT28605 | AE003659 | FBgn0032680 |
|  | 151 | 146456_at | 3.5596 | 0.02542 | *CG10600* | CT29714 | AE003660 | FBgn0032717 |
|  | 152 | 146468_at | 5.0479 | 0.000782 | *CG15171* | CT35080 | AE003661 | FBgn0032739 |
|  | 153 | 146477_at | 5.5198 | 0.001359 | *CG17344* | CT35082 | AE003661 | FBgn0032755 |
|  | 154 | 146593_s_at | 4.4597 | 0.025401 | *CG15190* | CT35106 | AE003781 | FBgn0032953 |
|  | 155 | 146735_at | 4.6799 | 0.002071 | *CG11113* | CT8225 | AE003841 | FBgn0033165 |
|  | 156 | 146791_at | 28.6464 | 0.010032 | *CG2160* | CT7060 | AE003838 | FBgn0033266 |
|  | 157 | 146800_at | 3.8463 | 0.014134 | *CG2280* | CT7579 | AE003838 | FBgn0033281 |
|  | 158 | 146814_at | 2.5625 | 0.048813 | *CG12780* | CT36767 | AE003836 | FBgn0033301 |
|  | 159 | 146870_at | 3.9605 | 0.004443 | *CG2412* | CT8006 | AE003834 | FBgn0033389 |
|  | 160 | 146889_at | 2.9765 | 0.036207 | *CG12931* | CT32079 | AE003833 | FBgn0033422 |
|  | 161 | 146890_at | 11.3016 | 0.010175 | *CG1874* | CT5782 | AE003833 | FBgn0033425 |
|  | 162 | 147063_at | 2.4352 | 0.046988 | *CG13174* | CT32415 | AE003823 | FBgn0033694 |
|  | 163 | 147077_at | 2.7404 | 0.046639 | *CG13163* | CT32404 | AE003822 | FBgn0033712 |
|  | 164 | 147112_at | 3.1441 | 0.015032 | *CG8771* | CT25298 | AE003821 | FBgn0033766 |
|  | 165 | 147163_at | 3 | 0.029559 | *CG6209* | CT19464 | AE003817 | FBgn0033862 |
|  | 166 | 147181_at | 2.6874 | 0.038826 | *CG18371* | CT41759 | AE003816 | FBgn0033893 |
|  | 167 | 147197_at | 9.856 | 0.002875 | *CG13941* | CT33487 | AE003815 | FBgn0033928 |
|  | 168 | 147202_at | 14.869 | 2.14E-08 | *CG17386* | CT33484 | AE003814 | FBgn0033936 |
|  | 169 | 147228_at | 2.7951 | 0.026325 | *CG10257* | CT28823 | AE003812 | FBgn0033985 |
|  | 170 | 147272_at | 2.7123 | 0.031113 | *CG8388* | CT18261 | AE003808 | FBgn0034062 |
|  | 171 | 147315_at | 2.6576 | 0.033364 | *CG4905* | CT15621 | AE003806 | FBgn0034135 |
|  | 172 | 147409_at | 37.8572 | 0.025901 | *CG5765* | CT18116 | AE003801 | FBgn0034294 |
|  | 173 | 147413_at | 4.772 | 0.02085 | *CG5756* | CT18091 | AE003800 | FBgn0034301 |
|  | 174 | 147437_at | 7.1777 | 0.017981 | *CG17525* | CT38743 | AE003799 | FBgn0034338 |
|  | 175 | 147459_at | 2.5677 | 0.044245 | *CG18609* | CT42569 | AE003798 | FBgn0034382 |
|  | 176 | 147462_at | 3.0071 | 0.020504 | *CG15075* | CT34950 | AE003798 | FBgn0034385 |
|  | 177 | 147702_at | 9.0235 | 0.049925 | *CG13522* | CT32893 | AE003458 | FBgn0034765 |
|  | 178 | 147734_at | 12.7366 | 0.031691 | *CG9895* | CT27882 | AE003459 | FBgn0034810 |
|  | 179 | 147856_at | 2.5599 | 0.041793 | *CG15874* | CT32972 | AE003464 | FBgn0035004 |
|  | 180 | 147913_at | 4.1015 | 0.046398 | *CG17142* | CT33412 | AE003467 | FBgn0035113 |
|  | 181 | 147957_at | 3.1766 | 0.046833 | *CG9094* | CT26098 | AE003470 | FBgn0035184 |
|  | 182 | 147960_at | 15.8351 | 9.87E-05 | *CG13913* | CT33450 | AE003470 | FBgn0035190 |
|  | 183 | 147990_at | 32.8807 | 0.029733 | *CG13926* | CT33465 | AE003472 | FBgn0035243 |
|  | 184 | 148005_at | 4.3562 | 0.002234 | *CG12023* | CT1769 | AE003472 | FBgn0035278 |
|  | 185 | 148047_at | 8.1186 | 0.033442 | *CG16763* | CT37281 | AE003475 | FBgn0035341 |
|  | 186 | 148070_at | 3.5855 | 0.010645 | *CG9972* | CT28115 | AE003476 | FBgn0035379 |
|  | 187 | 148121_at | 4.416 | 0.005099 | *CG10858* | CT30399 | AE003479 | FBgn0035458 |
|  | 188 | 148184_at | 9.7435 | 0.006802 | *CG13722* | CT33188 | AE003481 | FBgn0035553 |
|  | 189 | 148377_at | 5.5082 | 0.010805 | *CG7476* | CT22963 | AE003556 | FBgn0035847 |
|  | 190 | 148418_at | 8.589 | 9.00E-06 | *CG13305* | CT32600 | AE003554 | FBgn0035921 |
|  | 191 | 148513_at | 3.0944 | 0.038406 | *CG11965* | CT37129 | AE003549 | FBgn0036074 |
|  | 192 | 148529_at | 5.2594 | 0.000571 | *CG14153* | CT33756 | AE003547 | FBgn0036094 |
|  | 193 | 148560_at | 221.6597 | 0.004491 | *CG7624* | CT23263 | AE003546 | FBgn0036140 |
|  | 194 | 148568_at | 2.7105 | 0.031493 | *CG6175* | CT19380 | AE003545 | FBgn0036152 |
|  | 195 | 148704_at | 2.9799 | 0.021962 | *CG14107* | CT33701 | AE003538 | FBgn0036351 |
|  | 196 | 148745_at | 3.6132 | 0.012459 | *CG6419* | CT18675 | AE003535 | FBgn0036413 |
|  | 197 | 148863_at | 2.5164 | 0.047736 | *CG13065* | CT32284 | AE003528 | FBgn0036590 |
|  | 198 | 148870_at | 10.2334 | 2.29E-06 | *CG4962* | CT15908 | AE003528 | FBgn0036597 |
|  | 199 | 148941_at | 2.5059 | 0.044583 | *CG13726* | CT33192 | AE003524 | FBgn0036709 |
|  | 200 | 149060_at | 2.542 | 0.04043 | *CG9268* | CT26410 | AE003516 | FBgn0036885 |
|  | 201 | 149097_at | 3.5875 | 0.009582 | *CG13811* | CT33306 | AE003514 | FBgn0036944 |
|  | 202 | 149134_at | 3.3597 | 0.011235 | *CG17637* | CT38927 | AE003591 | FBgn0037004 |
|  | 203 | 149176_at | 2.892 | 0.034057 | *CG6968* | CT21581 | AE003594 | FBgn0037077 |
|  | 204 | 149201_at | 2.9483 | 0.027278 | *CG7155* | CT22117 | AE003595 | FBgn0037119 |
|  | 205 | 149215_at | 2.4503 | 0.045858 | *CG7442* | CT22915 | AE003596 | FBgn0037140 |
|  | 206 | 149303_at | 5.3379 | 0.000508 | *CG14661* | CT34439 | AE003604 | FBgn0037288 |
|  | 207 | 149398_at | 4.9089 | 0.006514 | *CG10296* | CT28911 | AE003675 | FBgn0037436 |
|  | 208 | 149420_at | 12.8588 | 4.20E-08 | *CG1943* | CT6062 | AE003673 | FBgn0037468 |
|  | 209 | 149492_at | 2.5895 | 0.037199 | *CG9601* | CT27152 | AE003678 | FBgn0037578 |
|  | 210 | 149599_at | 2.5884 | 0.042351 | *CG12419* | CT31932 | AE003685 | FBgn0037769 |
|  | 211 | 149615_at | 5.0821 | 0.000743 | *CG3940* | CT13118 | AE003685 | FBgn0037788 |
|  | 212 | 149700_at | 2.5295 | 0.04065 | *CG6834* | CT21175 | AE003692 | FBgn0037935 |
|  | 213 | 149737_at | 18.7096 | 0.00298 | *CG4830* | CT15487 | AE003695 | FBgn0037996 |
|  | 214 | 149742_at | 4.0613 | 0.004875 | *CG3916* | CT13055 | AE003695 | FBgn0038003 |
|  | 215 | 149754_at | 2.7536 | 0.030344 | *CG17392* | CT38399 | AE003695 | FBgn0038019 |
|  | 216 | 149800_at | 4.053 | 0.020094 | *CG14390* | CT34039 | AE003697 | FBgn0038084 |
|  | 217 | 149871_at | 3.3267 | 0.01123 | *CG3153* | CT10556 | AE003703 | FBgn0038198 |
|  | 218 | 149899_at | 2.9841 | 0.019427 | *Cyp313a1* | CT11271 | AE003705 | FBgn0038236 |
|  | 219 | 149903_at | 3.8551 | 0.012344 | *CG14851* | CT34667 | AE003705 | FBgn0038240 |
|  | 220 | 149984_at | 9.8942 | 0.007316 | *CG11846* | CT36983 | AE003710 | FBgn0038379 |
|  | 221 | 150125_at | 6.0833 | 0.002861 | *CG18599* | CT42541 | AE003721 | FBgn0038592 |
|  | 222 | 150128_at | 10.9293 | 0.003924 | *CG14312* | CT33942 | AE003721 | FBgn0038596 |
|  | 223 | 150143_at | 2.7284 | 0.040351 | *CG7688* | CT22823 | AE003722 | FBgn0038623 |
|  | 224 | 150149_at | 2.6163 | 0.04302 | *CG14304* | CT33934 | AE003722 | FBgn0038629 |
|  | 225 | 150226_at | 3.2744 | 0.047026 | *CG11667* | CT25514 | AE003728 | FBgn0038743 |
|  | 226 | 150240_at | 7.8053 | 0.004306 | *CG4854* | CT15583 | AE003728 | FBgn0038766 |
|  | 227 | 150314_at | 2.6644 | 0.032738 | *CG15503* | CT35611 | AE003734 | FBgn0038888 |
|  | 228 | 150365_at | 12.5112 | 0.001293 | *CG12499* | CT33360 | AE003739 | FBgn0038968 |
|  | 229 | 150375_at | 3.8909 | 0.004941 | *CG5326* | CT16950 | AE003739 | FBgn0038983 |
|  | 230 | 150385_at | 2.8396 | 0.024503 | *CG6949* | CT21531 | AE003739 | FBgn0038996 |
|  | 231 | 150428_at | 3.1842 | 0.014786 | *CG4624* | CT14942 | AE003742 | FBgn0039058 |
|  | 232 | 150454_at | 3.062 | 0.02227 | *CG10164* | CT28591 | AE003743 | FBgn0039088 |
|  | 233 | 150462_at | 3.3872 | 0.010612 | *CG16710* | CT37187 | AE003744 | FBgn0039101 |
|  | 234 | 150480_at | 2.9556 | 0.028912 | *CG10694* | CT29977 | AE003746 | FBgn0039147 |
|  | 235 | 150499_at | 3.0167 | 0.018435 | *CG6356* | CT19859 | AE003747 | FBgn0039178 |
|  | 236 | 150533_at | 3.3066 | 0.017479 | *CG13641* | CT33035 | AE003749 | FBgn0039239 |
|  | 237 | 150542_at | 2.5588 | 0.038781 | *CG13647* | CT33043 | AE003750 | FBgn0039256 |
|  | 238 | 150558_at | 935.5998 | 0.024816 | *CG13653* | CT33069 | AE003751 | FBgn0039288 |
|  | 239 | 150573_at | 3.3888 | 0.010171 | *CG11889* | CT33082 | AE003751 | FBgn0039308 |
|  | 240 | 150574_at | 21.5316 | 1.61E-11 | *CG11891* | CT37044 | AE003751 | FBgn0039309 |
|  | 241 | 150583_at | 3.3229 | 0.011297 | *CG13660* | CT33089 | AE003751 | FBgn0039320 |
|  | 242 | 150585_at | 23.7537 | 0.01284 | *CG10559* | CT29616 | AE003751 | FBgn0039323 |
|  | 243 | 150586_at | 13.0797 | 3.33E-08 | *CG10553* | CT29612 | AE003751 | FBgn0039324 |
|  | 244 | 150652_at | 3.2357 | 0.014895 | *CG14558* | CT34289 | AE003755 | FBgn0039416 |
|  | 245 | 150655_at | 3.6917 | 0.006295 | *CG6036* | CT18937 | AE003756 | FBgn0039421 |
|  | 246 | 150662_i_at | 2.6625 | 0.033375 | *CG5468* | CT17338 | AE003757 | FBgn0039434 |
|  | 247 | 150677_at | 3.895 | 0.005107 | *CG14249* | CT33869 | AE003757 | FBgn0039447 |
|  | 248 | 150697_at | 2.5088 | 0.041736 | *CG6309* | CT19744 | AE003758 | FBgn0039468 |
|  | 249 | 150762_at | 3.6561 | 0.006659 | *CG4869* | CT15625 | AE003763 | FBgn0039567 |
|  | 250 | 150788_at | 2.8076 | 0.030134 | *CG14525* | CT34252 | AE003767 | FBgn0039608 |
|  | 251 | 150807_at | 4.0334 | 0.0161 | *CG14516* | CT34241 | AE003768 | FBgn0039640 |
|  | 252 | 150826_at | 15.3743 | 0.010734 | *CG11470* | CT36281 | AE003770 | FBgn0039671 |
|  | 253 | 150858_at | 5.3796 | 0.000478 | *CG15518* | CT35631 | AE003771 | FBgn0039721 |
|  | 254 | 150889_at | 2.6022 | 0.045361 | *CG15537* | CT35652 | AE003774 | FBgn0039770 |
|  | 255 | 150907_at | 199.8211 | 0.04996 | *CG12069* | CT4640 | AE003775 | FBgn0039796 |
|  | 256 | 150908_at | 28.3693 | 0.033489 | *CG11313* | CT31571 | AE003775 | FBgn0039798 |
|  | 257 | 150958_at | 1401.6082 | 0.000713 | *CG11575* | CT35380 | AE003779 | FBgn0039879 |
|  | 258 | 151003_at | 2.7489 | 0.030243 | *Buffy* | CT24465 | AE003825 | FBgn0040491 |
|  | 259 | 151059_i_at | 15.0251 | 0.027348 | *CG17201* | CT35875 | AE003729 | FBgn0040572 |
|  | 260 | 151072_i_at | 30.8549 | 0.009009 | *CG13858* | CT33372 | AE003738 | FBgn0040585 |
|  | 261 | 151086_at | 28.7418 | 0.033963 | *CG13643* | CT33038 | AE003749 | FBgn0040601 |
|  | 262 | 151222_at | 3.7289 | 0.006337 | *CG12955* | CT32147 | AE003811 | FBgn0040746 |
|  | 263 | 151316_at | 2.7852 | 0.027464 | *CG15213* | CT35146 | AE003567 | FBgn0040843 |
|  | 264 | 151359_at | 10.2364 | 0.00755 | *CG12528* | CT33799 | AE003511 | FBgn0040886 |
|  | 265 | 151362_r_at | 6.2766 | 0.034674 | *CG14192* | CT33805 | AE003511 | FBgn0040889 |
|  | 266 | 151423_at | 160.5292 | 0.000107 | *CG15867* | CT32330 | AE003622 | FBgn0040961 |
|  | 267 | 151428_at | 2.688 | 0.043593 | *CG18666* | CT42597 | AE003630 | FBgn0040967 |
|  | 268 | 151433_at | 8.2119 | 9.57E-06 | *CG16978* | CT35582 | AE003639 | FBgn0040972 |
|  | 269 | 151485_s_at | 4.5794 | 0.001624 | *tim* | CT3982 |  | FBgn0014396 |
|  | 270 | 151568_at | 7.4432 | 2.63E-05 | *CG11326* | CT31613 |  | FBgn0031850 |
|  | 271 | 151617_s_at | 106.931 | 0.00928 | *CG8020* | CT2486 |  | FBgn0031009 |
|  | 272 | 151642_at | 3.7362 | 0.005869 | *CG17100* | CT34464 |  | FBgn0037813 |
|  | 273 | 151681_at | 3.2688 | 0.012316 | *CG12790* | CT37283 |  | FBgn0035342 |
|  | 274 | 151683_at | 2.4979 | 0.042475 | *CG14648* | CT3442 |  | FBgn0037245 |
|  | 275 | 151720_at | 3.5099 | 0.019363 | *CG4949* | CT15888 |  | FBgn0030813 |
|  | 276 | 151795_at | 5.1902 | 0.000633 | *CG7176* | CT22171 | AE003555 | FBgn0035881 |
|  | 277 | 151801_at | 2.7375 | 0.028882 | *CG12163* | CT8855 | AE003603 | FBgn0037303 |
|  | 278 | 151855_at | 2.7507 | 0.028273 | *BcDNA:GH02439* | CT6664 | AE003601 | FBgn0027608 |
|  | 279 | 151871_at | 14.0039 | 1.28E-08 | *BcDNA:GH02712* | CT6764 | AE003840 | FBgn0027604 |
|  | 280 | 151885_at | 4.7709 | 0.001186 | *BcDNA:GH02901* | CT25792 | AE003497 | FBgn0027601 |
|  | 281 | 151897_at | 4.0161 | 0.003784 | *CG4919* | CT15810 | AE003740 | FBgn0039001 |
|  | 282 | 151899_at | 4.4803 | 0.001846 | *CG11796* | CT36593 | AE003591 | FBgn0036992 |
|  | 283 | 151905_at | 2.4057 | 0.049276 | *Pkc53E* | CT20486 | AE003805 | FBgn0003091 |
|  | 284 | 151919_at | 3.5759 | 0.007559 | *CG16941* | CT37570 | AE003715 | FBgn0038464 |
|  | 285 | 151927_at | 6.9724 | 4.97E-05 | *CG4784* | CT15377 | AE003527 | FBgn0036619 |
|  | 286 | 151933_at | 3.6748 | 0.006464 | *BcDNA:GH03693* | CT12621 | AE003525 | FBgn0027593 |
|  | 287 | 151969_at | 2.6775 | 0.033808 | *CG6405* | CT19966 | AE003636 | FBgn0032428 |
|  | 288 | 151992_at | 3.3314 | 0.011145 | *CG10233* | CT28765 | AE003603 | FBgn0037302 |
|  | 289 | 152019_at | 3.979 | 0.004157 | *CG11853* | CT33074 | AE003751 | FBgn0039298 |
|  | 290 | 152057_at | 3.6377 | 0.006854 | *CG8324* | CT4932 | AE003784 | FBgn0033071 |
|  | 291 | 152086_at | 2.4316 | 0.047262 | *CG6409* | CT20018 | AE003547 | FBgn0036106 |
|  | 292 | 152102_at | 2.6129 | 0.036726 | *CG9119* | CT9987 | AE003470 | FBgn0035189 |
|  | 293 | 152113_at | 8.5017 | 6.59E-06 | *ana* | CT4622 | AE003834 | FBgn0011746 |
|  | 294 | 152124_at | 2.7476 | 0.029704 | *CG1017* | CT1020 | AE003473 | FBgn0035294 |
|  | 295 | 152139_at | 2.8556 | 0.023881 | *CG17352* | CT33100 | AE003555 | FBgn0035880 |
|  | 296 | 152156_at | 3.0889 | 0.016421 | *Cpn* | CT15219 | AE003694 | FBgn0010218 |
|  | 297 | 152192_at | 7.3985 | 2.79E-05 | *Cnx99A* | CT4036 | AE003769 | FBgn0015622 |
|  | 298 | 152256_at | 10.1546 | 1.12E-06 | *CG2121* | CT6910 | AE003837 | FBgn0033289 |
|  | 299 | 152261_at | 3.4527 | 0.00919 | *CAH1* | CT23642 | AE003641 | FBgn0027844 |
|  | 300 | 152308_at | 2.5427 | 0.039519 | *CG10433* | CT29298 | AE003454 | FBgn0034638 |
|  | 301 | 152324_at | 2.4284 | 0.047506 | *CG7148* | CT22091 | AE003595 | FBgn0037136 |
|  | 302 | 152382_at | 2.5067 | 0.04188 | *CG7149* | CT22085 | AE003618 | FBgn0031948 |
|  | 303 | 152418_at | 3.1196 | 0.015634 | *bnb* | CT21889 | AE003510 | FBgn0001090 |
|  | 304 | 152441_at | 3.1188 | 0.018351 | *CG4744* | CT15313 | AE003818 | FBgn0033834 |
|  | 305 | 152457_at | 2.8448 | 0.0243 | *CG3672* | CT12317 | AE003552 | FBgn0035985 |
|  | 306 | 152485_at | 4.9615 | 0.018568 | *CG4961* | CT15711 | AE003728 | FBgn0038770 |
|  | 307 | 152513_at | 14.8113 | 5.75E-09 | *CG10513* | CT29500 | AE003751 | FBgn0039311 |
|  | 308 | 152597_at | 3.4775 | 0.008835 | *SP2523* | CT14058 | AE003558 | FBgn0035808 |
|  | 309 | 152629_at | 2.9843 | 0.019421 | *CG5455* | CT17296 | AE003756 | FBgn0039430 |
|  | 310 | 152673_at | 7.1632 | 3.83E-05 | *cry* | CT12574 | AE003725 | FBgn0025680 |
|  | 311 | 152682_at | 16.1565 | 1.59E-09 | *Slob* | CT20985 | AE003618 | FBgn0024290 |
|  | 312 | 152715_at | 2.9451 | 0.020683 | *CG1600* | CT37723 | AE003840 | FBgn0033188 |
|  | 313 | 152737_at | 3.5213 | 0.017264 | *CG3342* | CT11209 | AE003437 | FBgn0029874 |
|  | 314 | 152767_at | 3.8166 | 0.005439 | *CG13631* | CT33025 | AE003749 | FBgn0040600 |
|  | 315 | 152781_at | 3.9934 | 0.003921 | *Pgd* | CT12475 | AE003423 | FBgn0004654 |
|  | 316 | 152819_at | 12.7789 | 4.57E-08 | *CG11407* | CT31802 | AE003727 | FBgn0038733 |
|  | 317 | 152839_at | 4.3987 | 0.002138 | *CG7388* | CT13674 | AE003557 | FBgn0035818 |
|  | 318 | 152855_at | 2.8502 | 0.03934 | *CG11546* | CT9385 | AE003838 | FBgn0033270 |
|  | 319 | 152958_at | 15.4078 | 3.22E-09 | *Cyp4d21* | CT20878 | AE003618 | FBgn0031925 |
|  | 320 | 152981_at | 3.3492 | 0.013018 | *CG7815* | CT23740 | AE003531 | FBgn0036497 |
|  | 321 | 152982_at | 4.8758 | 0.001013 | *CG18330* | CT1187 | AE003472 | FBgn0035231 |
|  | 322 | 153015_i_at | 2.5314 | 0.040249 | *CG1055* | CT1120 | AE003605 | FBgn0037269 |
|  | 323 | 153055_at | 4.1787 | 0.002939 | *Pdp1* | CT39809 | AE003557 | FBgn0016694 |
|  | 324 | 153059_at | 2.7035 | 0.030507 | *CG1136* | CT1836 | AE003480 | FBgn0035490 |
|  | 325 | 153061_at | 4.542 | 0.00168 | *CG3872* | CT12859 | AE003465 | FBgn0035055 |
|  | 326 | 153085_at | 2.5716 | 0.037726 | *Rh5* | CT16797 | AE003634 | FBgn0014019 |
|  | 327 | 153092_at | 14.7839 | 5.91E-09 | *Chd64* | CT34849 | AE003480 | FBgn0035499 |
|  | 328 | 153098_at | 2.4049 | 0.049338 | *CG16705* | CT37173 | AE003744 | FBgn0039102 |
|  | 329 | 153155_at | 3.4077 | 0.009871 | *TER94* | CT7768 | AE003831 | FBgn0024923 |
|  | 330 | 153179_at | 2.5073 | 0.041837 | *CG11866* | CT37014 | AE003830 | FBgn0033486 |
|  | 331 | 153230_at | 3.5226 | 0.008225 | *bbx* | CT3330 | AE003568 | FBgn0024251 |
|  | 332 | 153256_at | 2.6776 | 0.031804 | *CG3869* | CT41324 | AE003437 | FBgn0029870 |
|  | 333 | 153260_at | 4.1429 | 0.003107 | *Mipp1* | CT13676 | AE003527 | FBgn0026061 |
|  | 334 | 153282_at | 2.9492 | 0.020548 | *CG6983* | CT21627 | AE003555 | FBgn0035896 |
|  | 335 | 153296_at | 3.7324 | 0.005904 | *myo-inositol-1-phosphate-synthase* | CT31151 | AE003841 | FBgn0025885 |
|  | 336 | 153385_at | 4.4938 | 0.001809 | *CG4675* | CT14838 | AE003616 | FBgn0031899 |
|  | 337 | 153408_at | 8.6587 | 5.77E-06 | *CG14557* | CT34288 | AE003755 | FBgn0039414 |
|  | 338 | 153417_at | 2.5664 | 0.038042 | *CG5823* | CT18279 | AE003718 | FBgn0038515 |
|  | 339 | 153418_at | 2.7667 | 0.027554 | *CG7908* | CT23908 | AE003772 | FBgn0039734 |
|  | 340 | 153424_at | 5.6116 | 0.00034 | *5-HT2* | CT1149 | AE003605 | FBgn0013743 |
|  | 341 | 153429_at | 3.9162 | 0.004424 | *CG1561* | CT4048 | AE003486 | FBgn0030317 |
|  | 342 | 153433_at | 6.9206 | 5.34E-05 | *Pdh* | CT15746 | AE003527 | FBgn0011693 |
|  | 343 | 153554_at | 2.7273 | 0.03067 | *CG1621* | CT4338 | AE003841 | FBgn0033182 |
|  | 344 | 153624_at | 3.4828 | 0.008761 | *CG15162* | CT35063 | AE003659 | FBgn0032694 |
|  | 345 | 153696_at | 13.15 | 0.031209 | *CG10569* | CT29656 | AE003565 | FBgn0035634 |
|  | 346 | 153725_at | 4.5305 | 0.00171 | *CG3662* | CT12281 | AE003588 | FBgn0031285 |
|  | 347 | 153784_at | 3.0911 | 0.016363 | *a6* | CT12608 | AE003421 | FBgn0023130 |
|  | 348 | 153797_at | 4.1842 | 0.004103 | *CG7869* | CT23828 | AE003546 | FBgn0036120 |
|  | 349 | 153839_at | 2.5988 | 0.036105 | *nla* | CT19005 | AE003712 | FBgn0026629 |
|  | 350 | 153972_at | 2.7262 | 0.029409 | *CG18218* | CT41222 | AE003526 | FBgn0036645 |
|  | 351 | 153988_at | 3.408 | 0.01243 | *Prp18* | CT18573 | AE003724 | FBgn0027784 |
|  | 352 | 154066_at | 2.4584 | 0.045863 | *CG9089* | CT26088 | AE003504 | FBgn0030805 |
|  | 353 | 154150_at | 2.4188 | 0.04825 | *CG8430* | CT18945 | AE003808 | FBgn0034079 |
|  | 354 | 154158_at | 2.7041 | 0.032113 | *CG3496* | CT11785 | AE003460 | FBgn0034852 |
|  | 355 | 154176_at | 4.6351 | 0.001458 | *CG2827* | CT9666 | AE003462 | FBgn0034934 |
|  | 356 | 154188_at | 5.287 | 0.000548 | *CG4991* | CT15971 | AE003504 | FBgn0030817 |
|  | 357 | 154290_at | 2.6445 | 0.038455 | *CG11241* | CT31379 | AE003598 | FBgn0037186 |
|  | 358 | 154302_at | 4.5338 | 0.001701 | *CG2277* | CT7561 | AE003471 | FBgn0035204 |
|  | 359 | 154338_at | 2.9678 | 0.021014 | *CG10689* | CT29950 | AE003661 | FBgn0032759 |
|  | 360 | 154390_at | 2.7678 | 0.027506 | *CG8237* | CT8219 | AE003835 | FBgn0033350 |
|  | 361 | 154450_at | 2.9331 | 0.029118 | *abs* | CT34409 | AE003607 | FBgn0015331 |
|  | 362 | 154583_at | 3.7823 | 0.012686 | *CG12009* | CT1585 | AE003477 | FBgn0035430 |
|  | 363 | 154666_at | 2.7993 | 0.027381 | *CG3238* | CT10886 | AE003579 | FBgn0031540 |
|  | 364 | 154853_at | 2.5226 | 0.049998 | *CG5018* | CT16106 | AE003528 | FBgn0036578 |
|  | 365 | 154871_at | 2.7236 | 0.029535 | *CG11299* | CT31531 | AE003461 | FBgn0034897 |
|  | 366 | 154936_at | 2.7074 | 0.030315 | *CG9285* | CT26455 | AE003701 | FBgn0038184 |
|  | 367 | 154946_at | 3.044 | 0.017646 | *CG5546* | CT17568 | AE003522 | FBgn0036761 |
|  | 368 | 154963_at | 4.1551 | 0.003049 | *CG10237* | CT28775 | AE003662 | FBgn0032783 |
|  | 369 | 154967_at | 479.5116 | 0.034235 | *CG1905* | CT5898 | AE003487 | FBgn0030337 |
|  | 370 | 155049_at | 2.9482 | 0.020581 | *CG7129* | CT22037 | AE003721 | FBgn0038599 |
|  | 371 | 155103_at | 9.4482 | 0.013997 | *r-l* | CT11942 | AE003733 | FBgn0003257 |
|  | 372 | 155145_at | 2.93 | 0.021191 | *CG4094* | CT13458 | AE003438 | FBgn0029889 |
| **DD data (679)** | 1 | 141200_at | 3.3386 | 0.019086 | *CG9418* | CT26718 | AE003453 | FBgn0034594 |
|  | 2 | 141221_at | 3.1821 | 0.01275 | *CG8253* | CT20181 | AE003809 | FBgn0034046 |
|  | 3 | 141249_at | 4.7428 | 0.029964 | *CG8828* | CT25398 | AE003822 | FBgn0033740 |
|  | 4 | 141280_at | 4.2174 | 0.002306 | *CG10283* | CT28883 | AE003659 | FBgn0032681 |
|  | 5 | 141282_at | 4.123 | 0.005724 | *CG18543* | CT42340 | AE003555 | FBgn0035892 |
|  | 6 | 141293_at | 4.5593 | 0.011212 | *CG9362* | CT26603 | AE003682 | FBgn0037696 |
|  | 7 | 141313_at | 3.0248 | 0.017376 | *EG:34F3.7* | CT21087 | AE003418 | FBgn0025618 |
|  | 8 | 141318_at | 2.5751 | 0.035133 | *blow* | CT3102 | AE003840 | FBgn0004133 |
|  | 9 | 141319_at | 4.6695 | 0.003566 | *Cdlc2* | CT17290 | AE003586 | FBgn0026141 |
|  | 10 | 141324_at | 3.6572 | 0.019831 | *CG15818* | CT37153 | AE003617 | FBgn0031910 |
|  | 11 | 141377_at | 2.7589 | 0.029774 | *mas* | CT34855 | AE003480 | FBgn0011653 |
|  | 12 | 141408_at | 2.5184 | 0.041099 | *CG17930* | CT39946 | AE003712 | FBgn0038416 |
|  | 13 | 141416_at | 5.6591 | 0.000757 | *CG6839* | CT21185 | AE003519 | FBgn0036831 |
|  | 14 | 141433_at | 5.1272 | 0.005379 | *CG10757* | CT30148 | AE003665 | FBgn0032849 |
|  | 15 | 141513_at | 8.9981 | 0.004508 | *CG12109* | CT6479 | AE003444 | FBgn0030054 |
|  | 16 | 141521_at | 2.5845 | 0.034586 | *CG5202* | CT16128 | AE003634 | FBgn0032391 |
|  | 17 | 141565_at | 3.4926 | 0.019751 | *thr* | CT18154 | AE003801 | FBgn0003701 |
|  | 18 | 141573_at | 2.7562 | 0.025965 | *CG17647* | CT32528 | AE003585 | FBgn0031363 |
|  | 19 | 141593_at | 15.8891 | 4.73E-10 | *CG5798* | CT18196 | AE003733 | FBgn0038862 |
|  | 20 | 141673_at | 3.1185 | 0.014289 | *CG8291* | CT21678 | AE003809 | FBgn0034049 |
|  | 21 | 141719_at | 3.1817 | 0.012862 | *Rap2l* | CT10749 | AE003462 | FBgn0025806 |
|  | 22 | 141834_at | 2.9355 | 0.022357 | *CG18093* |  |  | FBgn0040661 |
|  | 23 | 141841_at | 2.8624 | 0.02564 | *CG9316* |  |  | FBgn0032879 |
|  | 24 | 141854_at | 2.9908 | 0.02375 | *bgcn* |  |  | FBgn0034916 |
|  | 25 | 141913_at | 2.8351 | 0.026442 | *CG4753* |  |  | FBgn0036623 |
|  | 26 | 141929_at | 2.6438 | 0.048498 | *RpL3* |  |  | FBgn0037880 |
|  | 27 | 141938_at | 2.4742 | 0.04814 | *CG11716* |  |  | FBgn0034802 |
|  | 28 | 141968_at | 2.5573 | 0.03676 | *dare* |  |  | FBgn0033610 |
|  | 29 | 142006_at | 3.1599 | 0.023254 | *CG6071* |  |  | FBgn0036187 |
|  | 30 | 142022_at | 2.9104 | 0.032343 | *CG5091* |  |  | FBgn0032235 |
|  | 31 | 142029_at | 2.6714 | 0.037431 | *CG2957* |  |  | FBgn0037530 |
|  | 32 | 142030_at | 3.7645 | 0.006109 | *CG14221* |  |  | FBgn0031043 |
|  | 33 | 142034_at | 3.6353 | 0.021816 | *CkIIalpha-i3* |  |  | FBgn0035158 |
|  | 34 | 142059_at | 2.781 | 0.028184 | *NUCB1* |  |  | FBgn0036754 |
|  | 35 | 142060_at | 3.4156 | 0.012293 | *CG12269* |  |  | FBgn0038666 |
|  | 36 | 142071_at | 80.2693 | 4.96E-09 | *CG8895* |  |  | FBgn0031667 |
|  | 37 | 142072_at | 2.5823 | 0.035672 | *CG17771* |  |  | FBgn0035322 |
|  | 38 | 142137_at | 5.5458 | 0.000332 | *ESTS:149B10S* | CT13989 | AE003558 | FBgn0017645 |
|  | 39 | 142154_at | 13.7788 | 4.77E-09 | *tim* | CT39082 | AE003579 | FBgn0014396 |
|  | 40 | 142156_at | 2.6709 | 0.030647 | *CG2056* | CT6615 | AE003444 | FBgn0030051 |
|  | 41 | 142190_at | 2.9497 | 0.018795 | *Cyp6a21* | CT28795 | AE003813 | FBgn0033981 |
|  | 42 | 142208_at | 7.8128 | 0.003122 | *CG5762* | CT18086 | AE003747 | FBgn0039190 |
|  | 43 | 142209_at | 7.5532 | 0.004524 | *CG11714* | CT36751 | AE003544 | FBgn0036170 |
|  | 44 | 142234_at | 3.0455 | 0.026279 | *bgcn* | CT38864 | AE003462 | FBgn0004581 |
|  | 45 | 142257_at | 3.6001 | 0.006363 | *BcDNA:GH10614* | CT30407 | AE003479 | FBgn0027552 |
|  | 46 | 142265_at | 5.1494 | 0.003749 | *CG16793* | CT33194 | AE003524 | FBgn0036712 |
|  | 47 | 142271_at | 8.4241 | 3.98E-06 | *Ugt35b* | CT20678 | AE003690 | FBgn0026314 |
|  | 48 | 142295_at | 2.5019 | 0.03969 | *tsr* | CT13858 | AE003462 | FBgn0011726 |
|  | 49 | 142317_at | 7.2473 | 0.004123 | *CG10298* | CT28915 | AE003675 | FBgn0037432 |
|  | 50 | 142321_at | 3.7201 | 0.021717 | *CG1394* | CT3204 | AE003485 | FBgn0030277 |
|  | 51 | 142327_at | 7.5998 | 0.015701 | *CG10859* | CT26435 | AE003640 | FBgn0032520 |
|  | 52 | 142371_at | 3.7245 | 0.031249 | *NaPi-T* | CT28711 | AE003813 | FBgn0016684 |
|  | 53 | 142406_at | 4.1543 | 0.005496 | *CG6931* | CT21474 | AE003543 | FBgn0036236 |
|  | 54 | 142432_at | 4.8499 | 0.008556 | *CG14937* | CT34765 | AE003633 | FBgn0032377 |
|  | 55 | 142459_at | 2.5666 | 0.036002 | *pan* | CT40032 | AE003845 | FBgn0019664 |
|  | 56 | 142476_at | 127.7957 | 6.19E-06 | *CG7783* | CT23343 | AE003594 | FBgn0037103 |
|  | 57 | 142477_at | 2.5239 | 0.049165 | *CG7369* | CT22651 | AE003598 | FBgn0037188 |
|  | 58 | 142495_at | 1.39E+16 | 0 | *Ant2* | CT4708 | AE003484 | FBgn0025111 |
|  | 59 | 142496_at | 2.6765 | 0.032372 | *CG15763* | CT36017 | AE003494 | FBgn0040869 |
|  | 60 | 142519_at | 2.9371 | 0.029651 | *CG17800* | CT39257 | AE003841 | FBgn0033159 |
|  | 61 | 142550_at | 2.6171 | 0.032758 | *l(3)mbt* | CT18705 | AE003760 | FBgn0002441 |
|  | 62 | 142580_at | 3.8076 | 0.006642 | *CG5272* | CT16839 | AE003533 | FBgn0036453 |
|  | 63 | 142591_at | 2.8029 | 0.027509 | *CG7540* | CT23079 | AE003594 | FBgn0037092 |
|  | 64 | 142608_at | 4.145 | 0.017157 | *CG11620* | CT33144 | AE003590 | FBgn0031226 |
|  | 65 | 142619_at | 2.4937 | 0.043661 | *CG18335* | CT41635 | AE003826 | FBgn0033610 |
|  | 66 | 142631_at | 2.9679 | 0.018233 | *aret* | CT19690 | AE003635 | FBgn0000114 |
|  | 67 | 142671_at | 68.1716 | 1.09E-07 | *CG13822* | CT33322 | AE003744 | FBgn0039098 |
|  | 68 | 142721_at | 3.6041 | 0.008571 | *CG13154* | CT32395 | AE003822 | FBgn0033736 |
|  | 69 | 142751_at | 10.9286 | 0.039118 | *CG15643* | CT35824 | AE003499 | FBgn0030654 |
|  | 70 | 142776_at | 2.51 | 0.041945 | *CG17680* | CT34212 | AE003800 | FBgn0034303 |
|  | 71 | 142780_at | 2.5618 | 0.038065 | *CG5704* | CT2731 | AE003474 | FBgn0035310 |
|  | 72 | 142809_at | 6.0767 | 0.004095 | *CG12258* | CT15916 | AE003708 | FBgn0038322 |
|  | 73 | 142816_at | 7.1571 | 0.000201 | *CG17801* | CT39442 | AE003719 | FBgn0038550 |
|  | 74 | 142819_at | 5.046 | 0.007245 | *E(spl)* | CT24645 | AE003754 | FBgn0000591 |
|  | 75 | 142828_at | 9.444 | 0.001146 | *CG15039* | CT34904 | AE003510 | FBgn0030957 |
|  | 76 | 142839_at | 3.6067 | 0.010335 | *BG:DS00276.8* | CT2039 | AE003674 | FBgn0026564 |
|  | 77 | 142865_at | 2.8819 | 0.025502 | *CG5589* | CT17674 | AE003522 | FBgn0036754 |
|  | 78 | 142883_at | 5.8803 | 0.00248 | *Orc4* | CT9892 | AE003465 | FBgn0023181 |
|  | 79 | 142938_at | 3.9483 | 0.008921 | *CG3108* | CT10418 | AE003435 | FBgn0029807 |
|  | 80 | 142996_at | 3.5065 | 0.015425 | *CG11033* | CT30887 | AE003681 | FBgn0037659 |
|  | 81 | 143002_at | 3.4743 | 0.007916 | *CG11428* | CT31897 | AE003493 | FBgn0030513 |
|  | 82 | 143052_at | 4.1927 | 0.014261 | *abd-A* | CT29034 | AE003715 | FBgn0000014 |
|  | 83 | 143087_at | 3.315 | 0.011175 | *br* | CT36317 | AE003421 | FBgn0000210 |
|  | 84 | 143099_i_at | 2.5614 | 0.049775 | *CecC* | CT2851 | AE003773 | FBgn0000279 |
|  | 85 | 143136_at | 3.0805 | 0.017611 | *bsh* | CT29614 | AE003664 | FBgn0000529 |
|  | 86 | 143144_at | 2.6874 | 0.033626 | *eg* | CT22725 | AE003595 | FBgn0000560 |
|  | 87 | 143149_at | 3.1627 | 0.022476 | *ems* | CT10075 | AE003702 | FBgn0000576 |
|  | 88 | 143166_at | 3.3933 | 0.01244 | *fs(1)K10* | CT10801 | AE003423 | FBgn0000810 |
|  | 89 | 143170_at | 6.0232 | 0.003546 | *fu* | CT20401 | AE003509 | FBgn0001079 |
|  | 90 | 143210_at | 4.9299 | 0.001958 | *kz* | CT10831 | AE003423 | FBgn0001330 |
|  | 91 | 143251_at | 17.5593 | 0.024782 | *m4* | CT19177 | AE003754 | FBgn0002629 |
|  | 92 | 143255_at | 92.964 | 0.002368 | *mei-9* | CT12377 | AE003430 | FBgn0002707 |
|  | 93 | 143271_at | 3.4021 | 0.008839 | *Mp20* | CT15161 | AE003819 | FBgn0002789 |
|  | 94 | 143272_at | 2.7724 | 0.029156 | *Acp26Aa* | CT25808 | AE003611 | FBgn0002855 |
|  | 95 | 143273_at | 3.021 | 0.02007 | *Acp26Ab* | CT25778 | AE003611 | FBgn0002856 |
|  | 96 | 143300_at | 4.7504 | 0.000991 | *per* | CT8963 | AE003425 | FBgn0003068 |
|  | 97 | 143315_at | 3.1639 | 0.013358 | *pum* | CT37227 | AE003681 | FBgn0003165 |
|  | 98 | 143331_at | 3.8093 | 0.023226 | *sala* | CT15718 | AE003632 | FBgn0003313 |
|  | 99 | 143333_at | 4.1013 | 0.004474 | *sca* | CT33383 | AE003821 | FBgn0003326 |
|  | 100 | 143354_at | 5.6582 | 0.000927 | *sli* | CT37068 | AE003809 | FBgn0003425 |
|  | 101 | 143362_at | 4.229 | 0.011104 | *spn-B* | CT11179 | AE003704 | FBgn0003480 |
|  | 102 | 143372_at | 3.0835 | 0.026509 | *svp* | CT12919 | AE003695 | FBgn0003651 |
|  | 103 | 143402_at | 2.9697 | 0.018438 | *vas* | CT11799 | AE003646 | FBgn0003970 |
|  | 104 | 143436_at | 2.4772 | 0.049995 | *Mst84Dd* | CT39964 | AE003672 | FBgn0004175 |
|  | 105 | 143438_at | 4.2443 | 0.014726 | *Ser* | CT18858 | AE003759 | FBgn0004197 |
|  | 106 | 143444_at | 3.7257 | 0.01288 | *T48* | CT17452 | AE003758 | FBgn0004359 |
|  | 107 | 143470_at | 3.8532 | 0.02581 | *LysX* | CT9967 | AE003470 | FBgn0004431 |
|  | 108 | 143487_at | 97.3866 | 1.17E-05 | *salm* | CT20082 | AE003632 | FBgn0004579 |
|  | 109 | 143492_at | 3.5135 | 0.048834 | *Eig71Ed* | CT22663 | AE003530 | FBgn0004591 |
|  | 110 | 143544_at | 2.9476 | 0.018861 | *sv* | CT30911 | AE003846 | FBgn0005561 |
|  | 111 | 143605_at | 2.5141 | 0.038893 | *Drs* | CT30304 | AE003478 | FBgn0010381 |
|  | 112 | 143606_at | 3.5416 | 0.007011 | *Cyp18a1* | CT20826 | AE003509 | FBgn0010383 |
|  | 113 | 143611_at | 2.5542 | 0.036947 | *Ket* | CT5932 | AE003473 | FBgn0010396 |
|  | 114 | 143648_at | 3.4717 | 0.019222 | *Pbprp4* | CT2057 | AE003672 | FBgn0011282 |
|  | 115 | 143666_at | 20.96 | 0.006572 | *fzo* | CT14814 | AE003742 | FBgn0011596 |
|  | 116 | 143667_at | 3.1209 | 0.014121 | *Iswi* | CT25027 | AE003821 | FBgn0011604 |
|  | 117 | 143704_s_at | 5.3561 | 0.001909 | *Mst35Ba* | CT14582 | AE003645 | FBgn0013300 |
|  | 118 | 143754_at | 3.2665 | 0.014719 | *gcm* | CT14310 | AE003625 | FBgn0014179 |
|  | 119 | 143773_at | 3.2167 | 0.017868 | *btn* | CT16825 | AE003739 | FBgn0014949 |
|  | 120 | 143803_at | 117.7638 | 1.61E-09 | *CG2034* | CT6534 | AE003474 | FBgn0015359 |
|  | 121 | 143825_at | 2.9921 | 0.021263 | *Acp53Ea* | CT25001 | AE003805 | FBgn0015584 |
|  | 122 | 143827_at | 3.5749 | 0.009537 | *Acp76A* | CT12717 | AE003518 | FBgn0015586 |
|  | 123 | 143860_at | 2.7969 | 0.04347 | *dpa* | CT4298 | AE003841 | FBgn0015929 |
|  | 124 | 143869_at | 25.9505 | 4.94E-14 | *vri* | CT33588 | AE003609 | FBgn0016076 |
|  | 125 | 143890_at | 2.576 | 0.043702 | *CG2187* | CT7152 | AE003779 | FBgn0017448 |
|  | 126 | 143891_at | 3.1058 | 0.031025 | *T3dh* | CT11499 | AE003457 | FBgn0017482 |
|  | 127 | 143921_at | 3.0456 | 0.022795 | *Tim17* | CT35201 | AE003647 | FBgn0020371 |
|  | 128 | 143926_at | 3.4953 | 0.00789 | *Glu-RIIB* | CT22307 | AE003610 | FBgn0020429 |
|  | 129 | 143936_at | 28.111 | 0.034547 | *Lcp65Af* | CT29553 | AE003563 | FBgn0020640 |
|  | 130 | 143943_at | 3.2541 | 0.019315 | *CG7179* | CT22169 | AE003618 | FBgn0020880 |
|  | 131 | 143944_at | 4.0228 | 0.00584 | *Ser4* | CT25434 | AE003608 | FBgn0020906 |
|  | 132 | 143946_at | 5.4825 | 0.004573 | *Ptx1* | CT3485 | AE003776 | FBgn0020912 |
|  | 133 | 143959_at | 2.7971 | 0.032048 | *D19B* | CT28859 | AE003563 | FBgn0022699 |
|  | 134 | 143970_at | 6.4256 | 7.18E-05 | *Clk* | CT13692 | AE003557 | FBgn0023076 |
|  | 135 | 143976_at | 3.7143 | 0.005327 | *Pp4-19C* | CT3537 | AE003571 | FBgn0023177 |
|  | 136 | 143984_at | 2.7889 | 0.034848 | *Acp32CD* | CT14870 | AE003630 | FBgn0023415 |
|  | 137 | 144028_at | 2.7611 | 0.025911 | *b6* | CT10412 | AE003421 | FBgn0024897 |
|  | 138 | 144033_at | 7.17E+15 | 0 | *RSG7* | CT26134 | AE003503 | FBgn0024941 |
|  | 139 | 144035_at | 4.197 | 0.003908 | *Oamb* | CT12841 | AE003731 | FBgn0024944 |
|  | 140 | 144053_at | 2.4515 | 0.049379 | *Optix* | CT42028 | AE003839 | FBgn0025360 |
|  | 141 | 144057_at | 20.8316 | 2.63E-07 | *EG:66A1.2* | CT42436 | AE003431 | FBgn0025388 |
|  | 142 | 144089_at | 2.6805 | 0.031903 | *pon* | CT11221 | AE003431 | FBgn0025739 |
|  | 143 | 144096_at | 3.7105 | 0.017495 | *Rad17* | CT23387 | AE003705 | FBgn0025808 |
|  | 144 | 144134_at | 3.069 | 0.023024 | *Or33B.1* | CT37631 | AE003634 | FBgn0026392 |
|  | 145 | 144208_at | 24.2265 | 1.24E-07 | *Rep2* | CT6211 | AE003834 | FBgn0028408 |
|  | 146 | 144224_at | 3.9699 | 0.004765 | *BG:DS07295.1* | CT13257 | AE003646 | FBgn0028516 |
|  | 147 | 144236_at | 3.6797 | 0.01937 | *A3-3* | CT31841 | AE003420 | FBgn0028550 |
|  | 148 | 144240_at | 17.3176 | 2.54E-06 | *beat-B* | CT23325 | AE003649 | FBgn0028645 |
|  | 149 | 144263_at | 2.6833 | 0.047267 | *BG:DS07851.5* | CT35208 | AE003647 | FBgn0028852 |
|  | 150 | 144270_at | 2.959 | 0.047028 | *BG:DS07473.1* | CT32536 | AE003650 | FBgn0028859 |
|  | 151 | 144299_at | 2.5213 | 0.041188 | *BG:DS02252.3* | CT40741 | AE003648 | FBgn0028901 |
|  | 152 | 144310_at | 3.1352 | 0.02479 | *BG:DS00941.12* | CT25774 | AE003641 | FBgn0028920 |
|  | 153 | 144327_at | 15.9427 | 0.023167 | *BG:BACR44L22.6* | CT23289 | AE003648 | FBgn0028945 |
|  | 154 | 144332_at | 3.173 | 0.015242 | *BG:BACR44L22.1* | CT35199 | AE003648 | FBgn0028950 |
|  | 155 | 144340_at | 3.8891 | 0.025044 | *CG13375* | CT32707 | AE003417 | FBgn0029519 |
|  | 156 | 144356_at | 7.9047 | 0.037481 | *CG11663* | CT34392 | AE003419 | FBgn0029539 |
|  | 157 | 144368_at | 3.2249 | 0.014253 | *CG11392* | CT31794 | AE003420 | FBgn0029560 |
|  | 158 | 144374_at | 5.0164 | 0.003867 | *CG11380* | CT31768 | AE003420 | FBgn0029566 |
|  | 159 | 144395_at | 2.715 | 0.032933 | *CG14053* | CT33614 | AE003423 | FBgn0029603 |
|  | 160 | 144409_at | 3.889 | 0.028246 | *CG13760* | CT33241 | AE003424 | FBgn0029625 |
|  | 161 | 144410_at | 3.0721 | 0.020712 | *CG17437* | CT33240 | AE003424 | FBgn0029626 |
|  | 162 | 144417_at | 3.0163 | 0.021738 | *CG14417* | CT34074 | AE003425 | FBgn0029637 |
|  | 163 | 144468_at | 809.2178 | 2.96E-09 | *CG15573* | CT35690 | AE003430 | FBgn0029698 |
|  | 164 | 144472_at | 28.6994 | 6.92E-07 | *CG12691* | CT35688 | AE003430 | FBgn0029701 |
|  | 165 | 144492_at | 70.3689 | 7.11E-10 | *CG15471* | CT35545 | AE003432 | FBgn0029726 |
|  | 166 | 144512_at | 2.6651 | 0.044966 | *CG3323* | CT11173 | AE003433 | FBgn0029750 |
|  | 167 | 144516_at | 3.7877 | 0.005789 | *CG3309* | CT11119 | AE003434 | FBgn0029756 |
|  | 168 | 144528_at | 4.0987 | 0.004034 | *CG15781* | CT36045 | AE003434 | FBgn0029774 |
|  | 169 | 144569_at | 15.6662 | 0.012817 | *CG5921* | CT18581 | AE003436 | FBgn0029835 |
|  | 170 | 144578_at | 7.8013 | 0.00608 | *CG4222* | CT13712 | AE003436 | FBgn0029845 |
|  | 171 | 144579_at | 21.2151 | 4.44E-06 | *CG15899* | CT34118 | AE003436 | FBgn0029846 |
|  | 172 | 144585_at | 2.8409 | 0.02254 | *CG3781* | CT12645 | AE003437 | FBgn0029853 |
|  | 173 | 144601_at | 3.3071 | 0.021438 | *CG14443* | CT34105 | AE003438 | FBgn0029880 |
|  | 174 | 144609_at | 3.6104 | 0.008668 | *CG4095* | CT13576 | AE003438 | FBgn0029890 |
|  | 175 | 144625_at | 3.4854 | 0.010232 | *CG14434* | CT34096 | AE003439 | FBgn0029915 |
|  | 176 | 144639_at | 2.8697 | 0.033299 | *CG14427* | CT34088 | AE003439 | FBgn0029931 |
|  | 177 | 144680_at | 1.32E+16 | 0 | *CG2120* | CT6904 | AE003443 | FBgn0030005 |
|  | 178 | 144721_at | 3.5582 | 0.007885 | *CG15364* | CT35383 | AE003446 | FBgn0030075 |
|  | 179 | 144734_at | 3.9454 | 0.004226 | *CG12115* | CT6912 | AE003446 | FBgn0030097 |
|  | 180 | 144741_at | 6.5635 | 0.000914 | *CG15371* | CT35392 | AE003446 | FBgn0030108 |
|  | 181 | 144755_at | 2.5064 | 0.039399 | *CG17251* | CT35403 | AE003447 | FBgn0030129 |
|  | 182 | 144760_at | 2.8795 | 0.026293 | *CG15317* | CT35303 | AE003447 | FBgn0030137 |
|  | 183 | 144765_at | 3.3512 | 0.020301 | *CG15321* | CT35308 | AE003448 | FBgn0030150 |
|  | 184 | 144797_at | 2.8988 | 0.037008 | *CG2881* | CT7274 | AE003450 | FBgn0030199 |
|  | 185 | 144873_at | 4.9388 | 0.00717 | *CG12622* | CT35117 | AE003486 | FBgn0030297 |
|  | 186 | 144909_at | 355.2602 | 0.039768 | *CG18130* | CT40826 | AE003487 | FBgn0030359 |
|  | 187 | 144932_at | 3.1984 | 0.018338 | *CG15732* | CT35969 | AE003489 | FBgn0030385 |
|  | 188 | 144954_at | 4.0022 | 0.010457 | *CG15727* | CT35960 | AE003490 | FBgn0030410 |
|  | 189 | 144961_at | 4.8376 | 0.031202 | *CG12717* | CT35954 | AE003490 | FBgn0030420 |
|  | 190 | 144964_at | 2.9672 | 0.01865 | *CG18453* | CT12689 | AE003490 | FBgn0030423 |
|  | 191 | 144972_at | 3.4828 | 0.008142 | *CG4400* | CT4159 | AE003490 | FBgn0030434 |
|  | 192 | 144994_at | 2.7645 | 0.027652 | *CG15742* | CT35990 | AE003492 | FBgn0030462 |
|  | 193 | 145042_at | 3.0547 | 0.022978 | *CG11068* | CT30965 | AE003494 | FBgn0030536 |
|  | 194 | 145065_i_at | 3.9925 | 0.003567 | *CG18313* | CT41559 | AE003495 | FBgn0030566 |
|  | 195 | 145068_at | 2.6075 | 0.034434 | *CG14415* | CT34072 | AE003495 | FBgn0030568 |
|  | 196 | 145084_at | 4.1233 | 0.00762 | *CG9521* | CT26940 | AE003497 | FBgn0030588 |
|  | 197 | 145088_at | 1.57E+16 | 0 | *CG14406* | CT34057 | AE003497 | FBgn0030595 |
|  | 198 | 145127_s_at | 5.2534 | 0.006097 | *CG18620* | CT41188 | AE003499 | FBgn0030652 |
|  | 199 | 145149_at | 7.6264 | 0.000445 | *CG8260* | CT24475 | AE003500 | FBgn0030684 |
|  | 200 | 145151_at | 3.0738 | 0.021457 | *CG8288* | CT24509 | AE003500 | FBgn0030686 |
|  | 201 | 145207_at | 200.4028 | 0.049888 | *CG13007* | CT32214 | AE003503 | FBgn0030783 |
|  | 202 | 145210_at | 4.6418 | 0.009268 | *CG4742* | CT15291 | AE003503 | FBgn0030786 |
|  | 203 | 145228_at | 3.2066 | 0.021201 | *CG8936* | CT25658 | AE003504 | FBgn0030818 |
|  | 204 | 145259_at | 2.3884 | 0.047946 | *CG5898* | CT18491 | AE003506 | FBgn0030865 |
|  | 205 | 145272_at | 2.7508 | 0.030478 | *CG12672* | CT35411 | AE003507 | FBgn0030886 |
|  | 206 | 145287_at | 4.6085 | 0.010755 | *CG15059* | CT34926 | AE003508 | FBgn0030905 |
|  | 207 | 145288_at | 5.6232 | 0.000662 | *CG15063* | CT34930 | AE003508 | FBgn0030906 |
|  | 208 | 145301_at | 2.455 | 0.043529 | *CG15050* | CT34917 | AE003508 | FBgn0030922 |
|  | 209 | 145312_at | 4.3452 | 0.012076 | *CG15042* | CT34908 | AE003509 | FBgn0030937 |
|  | 210 | 145313_at | 2.8666 | 0.028024 | *CG15047* | CT34913 | AE003509 | FBgn0030938 |
|  | 211 | 145325_at | 3.4402 | 0.012127 | *CG6894* | CT21348 | AE003510 | FBgn0030954 |
|  | 212 | 145326_s_at | 2.366 | 0.049971 | *CG18259* | CT41379 | AE003510 | FBgn0030956 |
|  | 213 | 145330_at | 3.7437 | 0.012607 | *CG7103* | CT21961 | AE003510 | FBgn0030964 |
|  | 214 | 145387_at | 2.9649 | 0.021611 | *CG14223* | CT33837 | AE003512 | FBgn0031053 |
|  | 215 | 145410_at | 2.6417 | 0.040224 | *CG9570* | CT17340 | AE002611 | FBgn0031085 |
|  | 216 | 145411_at | 6.1561 | 0.000183 | *CG9571* | CT17746 | AE002611 | FBgn0031086 |
|  | 217 | 145424_at | 5.0202 | 0.028458 | *CG11748* | CT35531 | AE003571 | FBgn0031109 |
|  | 218 | 145446_at | 3.5858 | 0.015242 | *CG11230* | CT31351 | AE003570 | FBgn0031137 |
|  | 219 | 145461_at | 3.4413 | 0.012391 | *CG11569* | CT36500 | AE003569 | FBgn0031158 |
|  | 220 | 145524_at | 3.5935 | 0.026711 | *CG11592* | CT33137 | AE003590 | FBgn0031246 |
|  | 221 | 145532_at | 6.441 | 0.001347 | *CG4133* | CT13710 | AE003589 | FBgn0031257 |
|  | 222 | 145542_at | 3.6429 | 0.014226 | *CG13686* | CT33124 | AE003588 | FBgn0031271 |
|  | 223 | 145570_at | 2.9921 | 0.017509 | *CG5156* | CT16503 | AE003587 | FBgn0031326 |
|  | 224 | 145572_at | 3.6779 | 0.048487 | *CG14344* | CT33977 | AE003586 | FBgn0031329 |
|  | 225 | 145581_at | 3.2109 | 0.02874 | *CG5574* | CT17328 | AE003586 | FBgn0031338 |
|  | 226 | 145585_at | 6.7122 | 0.002183 | *CG18132* | CT40836 | AE003586 | FBgn0031345 |
|  | 227 | 145605_at | 4.2936 | 0.002621 | *CG15360* | CT35369 | AE003585 | FBgn0031370 |
|  | 228 | 145652_at | 9.7212 | 5.45E-06 | *CG15394* | CT35443 | AE003582 | FBgn0031439 |
|  | 229 | 145661_at | 3.4143 | 0.012062 | *CG15397* | CT35447 | AE003582 | FBgn0031448 |
|  | 230 | 145697_at | 4.0797 | 0.00959 | *CG3347* | CT11239 | AE003580 | FBgn0031513 |
|  | 231 | 145724_at | 3.0598 | 0.023324 | *CG15416* | CT35475 | AE003579 | FBgn0031552 |
|  | 232 | 145728_at | 3.7867 | 0.004674 | *CG16704* | CT37177 | AE003579 | FBgn0031558 |
|  | 233 | 145740_at | 16.4242 | 0.002682 | *CG15420* | CT35479 | AE003578 | FBgn0031576 |
|  | 234 | 145749_at | 6.2672 | 0.006939 | *CG15425* | CT35487 | AE003577 | FBgn0031591 |
|  | 235 | 145751_at | 2.8379 | 0.023101 | *CG15426* | CT35488 | AE003577 | FBgn0031593 |
|  | 236 | 145767_at | 2.5034 | 0.047001 | *CG11933* | CT35796 | AE003575 | FBgn0031615 |
|  | 237 | 145770_at | 18.209 | 6.79E-06 | *CG11931* | CT35793 | AE003575 | FBgn0031618 |
|  | 238 | 145777_at | 2.3996 | 0.048648 | *CG15631* | CT35786 | AE003575 | FBgn0031626 |
|  | 239 | 145819_at | 3.0143 | 0.016872 | *CG14022* | CT33581 | AE003609 | FBgn0031700 |
|  | 240 | 145835_at | 2.8988 | 0.03053 | *CG14010* | CT33567 | AE003610 | FBgn0031725 |
|  | 241 | 145880_at | 4.0817 | 0.008985 | *CG9481* | CT26858 | AE003613 | FBgn0031790 |
|  | 242 | 145914_at | 15.502 | 7.19E-05 | *CG11053* | CT30923 | AE003614 | FBgn0031838 |
|  | 243 | 145928_at | 6.8127 | 0.000853 | *CG17373* | CT33257 | AE003615 | FBgn0031862 |
|  | 244 | 145941_at | 2.8419 | 0.025605 | *CG13780* | CT33268 | AE003616 | FBgn0031888 |
|  | 245 | 145957_at | 4.5059 | 0.011537 | *CG13787* | CT33276 | AE003617 | FBgn0031916 |
|  | 246 | 145969_at | 3.9188 | 0.006515 | *CG7025* | CT21646 | AE003618 | FBgn0031930 |
|  | 247 | 145973_at | 4.1308 | 0.013369 | *CG13793* | CT33282 | AE003618 | FBgn0031935 |
|  | 248 | 146022_at | 6.9347 | 3.36E-05 | *CG14275* | CT33900 | AE003620 | FBgn0032022 |
|  | 249 | 146047_at | 2.976 | 0.036228 | *CG12437* | CT32326 | AE003622 | FBgn0032064 |
|  | 250 | 146055_at | 3.5545 | 0.015831 | *CG17789* | CT39404 | AE003623 | FBgn0032081 |
|  | 251 | 146081_at | 4.0008 | 0.007283 | *CG13110* | CT32347 | AE003624 | FBgn0032111 |
|  | 252 | 146116_at | 3.1197 | 0.031934 | *CG13131* | CT32369 | AE003627 | FBgn0032175 |
|  | 253 | 146155_at | 5.5618 | 0.003719 | *CG5356* | CT17024 | AE003628 | FBgn0032241 |
|  | 254 | 146167_at | 6.5412 | 6.04E-05 | *CG18302* | CT41543 | AE003629 | FBgn0032266 |
|  | 255 | 146182_at | 2.4043 | 0.046695 | *CG7296* | CT22519 | AE003629 | FBgn0032283 |
|  | 256 | 146200_at | 2.8905 | 0.040166 | *CG17139* | CT38068 | AE003630 | FBgn0032307 |
|  | 257 | 146227_at | 2.5296 | 0.038478 | *CG16854* | CT34742 | AE003631 | FBgn0032338 |
|  | 258 | 146233_at | 2.5308 | 0.038012 | *CG4779* | CT15337 | AE003631 | FBgn0032349 |
|  | 259 | 146239_at | 8.3203 | 0.001019 | *CG16917* | CT37530 | AE003631 | FBgn0032357 |
|  | 260 | 146254_at | 13.4001 | 6.13E-06 | *CG14940* | CT34768 | AE003633 | FBgn0032379 |
|  | 261 | 146266_at | 3.688 | 0.038577 | *CG6792* | CT21067 | AE003634 | FBgn0032401 |
|  | 262 | 146275_at | 2.5313 | 0.038578 | *CG17212* | CT34787 | AE003635 | FBgn0032415 |
|  | 263 | 146302_at | 4.4001 | 0.010355 | *CG17176* | CT38108 | AE003638 | FBgn0032460 |
|  | 264 | 146324_at | 2.459 | 0.045517 | *CG16821* | CT35572 | AE003639 | FBgn0032496 |
|  | 265 | 146327_at | 7.6644 | 0.023189 | *CG16823* | CT35816 | AE003639 | FBgn0032500 |
|  | 266 | 146338_at | 3.4371 | 0.034882 | *CG16956* | CT35807 | AE003640 | FBgn0032523 |
|  | 267 | 146354_at | 2.7423 | 0.037207 | *CG4650* | CT14964 | AE003645 | FBgn0032549 |
|  | 268 | 146361_at | 3.0638 | 0.015772 | *CG13244* | CT32494 | AE003649 | FBgn0032577 |
|  | 269 | 146412_at | 2.7165 | 0.042967 | *CG7094* | CT21551 | AE003655 | FBgn0032650 |
|  | 270 | 146442_at | 4.3685 | 0.008856 | *CG15161* | CT35062 | AE003659 | FBgn0032692 |
|  | 271 | 146513_at | 2.6156 | 0.036261 | *CG10466* | CT29390 | AE003664 | FBgn0032822 |
|  | 272 | 146516_at | 4.0233 | 0.024452 | *CG13958* | CT33510 | AE003664 | FBgn0032825 |
|  | 273 | 146527_at | 7.3714 | 0.000644 | *CG13966* | CT33519 | AE003664 | FBgn0032838 |
|  | 274 | 146531_at | 2.68 | 0.04511 | *CG10730* | CT30075 | AE003665 | FBgn0032843 |
|  | 275 | 146538_at | 1009.2582 | 0.023603 | *CG13971* | CT33524 | AE003665 | FBgn0032852 |
|  | 276 | 146545_at | 2.8309 | 0.027924 | *CG17485* | CT38645 | AE003667 | FBgn0032866 |
|  | 277 | 146547_at | 3.4433 | 0.012073 | *CG17472* | CT38629 | AE003667 | FBgn0032868 |
|  | 278 | 146552_at | 7.0251 | 0.007519 | *CG9329* | CT3799 | AE003668 | FBgn0032887 |
|  | 279 | 146553_at | 3.9769 | 0.005492 | *CG14405* | CT34054 | AE003668 | FBgn0032888 |
|  | 280 | 146581_at | 11.778 | 0.006315 | *CG8672* | CT7956 | AE003669 | FBgn0032939 |
|  | 281 | 146590_s_at | 2.9699 | 0.036495 | *CG14465* | CT34156 | AE003781 | FBgn0032950 |
|  | 282 | 146614_at | 6.6485 | 0.000414 | *CG16948* | CT37594 | AE003783 | FBgn0032980 |
|  | 283 | 146626_at | 2.9496 | 0.029893 | *CG17479* | CT38649 | AE002751 | FBgn0032996 |
|  | 284 | 146640_at | 4.3213 | 0.011834 | *CG10453* | CT29252 | AE003787 | FBgn0033018 |
|  | 285 | 146670_at | 5.0852 | 0.000783 | *CG7849* | CT5216 | AE003784 | FBgn0033060 |
|  | 286 | 146701_at | 10.0154 | 0.023228 | *CG9455* | CT11129 | AE003790 | FBgn0033113 |
|  | 287 | 146718_at | 2.7575 | 0.025911 | *CG12837* | CT31969 | AE003842 | FBgn0033139 |
|  | 288 | 146722_at | 15.2081 | 3.18E-06 | *CG12833* | CT31965 | AE003842 | FBgn0033143 |
|  | 289 | 146730_at | 3.7869 | 0.024554 | *CG11101* | CT31067 | AE003841 | FBgn0033157 |
|  | 290 | 146737_at | 3.1896 | 0.018167 | *CG1701* | CT4794 | AE003841 | FBgn0033167 |
|  | 291 | 146779_at | 3.3838 | 0.009569 | *CG2906* | CT9908 | AE003839 | FBgn0033240 |
|  | 292 | 146836_at | 3.4025 | 0.01829 | *CG13754* | CT33231 | AE003835 | FBgn0033332 |
|  | 293 | 146862_at | 2.9432 | 0.02184 | *CG13742* | CT33217 | AE003834 | FBgn0033372 |
|  | 294 | 146875_at | 4.904 | 0.004754 | *CG13739* | CT33214 | AE003833 | FBgn0033403 |
|  | 295 | 146887_at | 3.5635 | 0.019282 | *CG1931* | CT5969 | AE003833 | FBgn0033420 |
|  | 296 | 146896_at | 2.9372 | 0.033054 | *CG1863* | CT5742 | AE003832 | FBgn0033435 |
|  | 297 | 146920_at | 2.4411 | 0.04684 | *CG12920* | CT32066 | AE003831 | FBgn0033481 |
|  | 298 | 146930_at | 4.0374 | 0.016039 | *CG12914* | CT32059 | AE003830 | FBgn0033499 |
|  | 299 | 146935_at | 2.6972 | 0.031061 | *CG3298* | CT3817 | AE003830 | FBgn0033506 |
|  | 300 | 146938_at | 3.2553 | 0.032345 | *CG12904* | CT32049 | AE003830 | FBgn0033510 |
|  | 301 | 146945_at | 19.54 | 7.27E-06 | *CG12897* | CT32042 | AE003829 | FBgn0033517 |
|  | 302 | 146975_at | 10.4687 | 0.020506 | *CG12943* | CT32091 | AE003827 | FBgn0033572 |
|  | 303 | 146976_at | 2.5973 | 0.039879 | *CG12944* | CT32092 | AE003827 | FBgn0033573 |
|  | 304 | 146996_at | 2.4447 | 0.045716 | *CG13224* | CT32468 | AE003826 | FBgn0033598 |
|  | 305 | 147013_at | 2.7459 | 0.032598 | *CG13209* | CT32453 | AE003826 | FBgn0033618 |
|  | 306 | 147026_at | 2.7183 | 0.027663 | *CG8998* | CT25866 | AE003825 | FBgn0033641 |
|  | 307 | 147051_at | 4213.9769 | 6.20E-06 | *CG13187* | CT32428 | AE003823 | FBgn0033678 |
|  | 308 | 147076_at | 2.4059 | 0.04988 | *CG17740* | CT39323 | AE003822 | FBgn0033711 |
|  | 309 | 147085_at | 3.9921 | 0.010576 | *CG8496* | CT24851 | AE003822 | FBgn0033722 |
|  | 310 | 147089_at | 4.5495 | 0.002895 | *CG8510* | CT24877 | AE003822 | FBgn0033729 |
|  | 311 | 147091_at | 2.8127 | 0.027088 | *CG8515* | CT24893 | AE003822 | FBgn0033731 |
|  | 312 | 147118_at | 2.7446 | 0.035162 | *CG12374* | CT25084 | AE003821 | FBgn0033774 |
|  | 313 | 147126_i_at | 6.1776 | 0.001287 | *CG13323* | CT32639 | AE003820 | FBgn0033788 |
|  | 314 | 147146_at | 2.39E+16 | 0 | *CG17048* | CT30288 | AE003818 | FBgn0033828 |
|  | 315 | 147149_at | 150.2172 | 0.000131 | *CG17049* | CT30302 | AE003818 | FBgn0033832 |
|  | 316 | 147160_at | 2.4065 | 0.046522 | *CG13335* | CT32654 | AE003818 | FBgn0033858 |
|  | 317 | 147173_at | 5.3038 | 0.008189 | *CG13342* | CT32662 | AE003817 | FBgn0033877 |
|  | 318 | 147174_at | 2.8413 | 0.023297 | *CG6553* | CT20424 | AE003817 | FBgn0033880 |
|  | 319 | 147181_at | 3.5833 | 0.018861 | *CG18371* | CT41759 | AE003816 | FBgn0033893 |
|  | 320 | 147202_at | 7.6997 | 1.20E-05 | *CG17386* | CT33484 | AE003814 | FBgn0033936 |
|  | 321 | 147205_at | 2.9406 | 0.023639 | *CG17390* | CT33481 | AE003814 | FBgn0033939 |
|  | 322 | 147212_at | 4.7015 | 0.00289 | *CG12862* | CT32002 | AE003813 | FBgn0033950 |
|  | 323 | 147214_at | 6.6097 | 0.000271 | *CG12861* | CT32001 | AE003813 | FBgn0033953 |
|  | 324 | 147215_at | 4.7609 | 0.01381 | *CG12860* | CT32000 | AE003813 | FBgn0033954 |
|  | 325 | 147223_at | 1.28E+16 | 0 | *CG12856* | CT31996 | AE003813 | FBgn0033966 |
|  | 326 | 147271_at | 2.7167 | 0.042953 | *CG8320* | CT18517 | AE003808 | FBgn0034059 |
|  | 327 | 147279_at | 2.4064 | 0.046534 | *CG18658* | CT18815 | AE003808 | FBgn0034075 |
|  | 328 | 147280_at | 2.6832 | 0.029671 | *CG8424* | CT18838 | AE003808 | FBgn0034076 |
|  | 329 | 147293_at | 8.1717 | 0.007751 | *CG15702* | CT35923 | AE003807 | FBgn0034101 |
|  | 330 | 147299_at | 4.7059 | 0.042028 | *CG7750* | CT23586 | AE003807 | FBgn0034107 |
|  | 331 | 147318_at | 3.8731 | 0.006363 | *CG4927* | CT15818 | AE003806 | FBgn0034139 |
|  | 332 | 147345_at | 4.1896 | 0.008492 | *CG9642* | CT27254 | AE003804 | FBgn0034183 |
|  | 333 | 147349_at | 2.7842 | 0.047421 | *CG15613* | CT35749 | AE003804 | FBgn0034192 |
|  | 334 | 147380_at | 2.4643 | 0.045129 | *CG6477* | CT20179 | AE003802 | FBgn0034249 |
|  | 335 | 147392_at | 2.4254 | 0.046644 | *CG10933* | CT30623 | AE003802 | FBgn0034264 |
|  | 336 | 147417_at | 2.9626 | 0.034165 | *CG10915* | CT30561 | AE003800 | FBgn0034308 |
|  | 337 | 147466_at | 3.9495 | 0.011095 | *CG15080* | CT34955 | AE003798 | FBgn0034391 |
|  | 338 | 147559_at | 4.7079 | 0.001788 | *CG13436* | CT32794 | AE003791 | FBgn0034532 |
|  | 339 | 147567_at | 19.4472 | 0.047767 | *CG13437* | CT32795 | AE003791 | FBgn0034541 |
|  | 340 | 147583_at | 3.3145 | 0.026942 | *CG10036* | CT28181 | AE003452 | FBgn0034559 |
|  | 341 | 147617_at | 2.9144 | 0.02007 | *CG10500* | CT29476 | AE003453 | FBgn0034619 |
|  | 342 | 147634_at | 2.5397 | 0.039989 | *CG7975* | CT24000 | AE003455 | FBgn0034658 |
|  | 343 | 147635_at | 11.1388 | 0.009553 | *CG4021* | CT13142 | AE003455 | FBgn0034659 |
|  | 344 | 147636_at | 4.2438 | 0.00289 | *CG13492* | CT32860 | AE003455 | FBgn0034662 |
|  | 345 | 147639_at | 3.3959 | 0.010619 | *CG13493* | CT32861 | AE003455 | FBgn0034667 |
|  | 346 | 147660_at | 4.4334 | 0.005309 | *CG11269* | CT31459 | AE003456 | FBgn0034700 |
|  | 347 | 147668_at | 3.8539 | 0.01236 | *CG3387* | CT11303 | AE003456 | FBgn0034714 |
|  | 348 | 147703_at | 7.3373 | 0.039708 | *CG13517* | CT32888 | AE003458 | FBgn0034766 |
|  | 349 | 147714_at | 2.8465 | 0.032824 | *CG13528* | CT32902 | AE003458 | FBgn0034779 |
|  | 350 | 147753_at | 3.4463 | 0.024779 | *CG13548* | CT32923 | AE003459 | FBgn0034832 |
|  | 351 | 147770_at | 3.6133 | 0.009231 | *CG3493* | CT11783 | AE003460 | FBgn0034854 |
|  | 352 | 147778_at | 17.781 | 2.09E-05 | *CG12227* | CT12747 | AE003460 | FBgn0034863 |
|  | 353 | 147861_at | 3.0921 | 0.019753 | *CG13589* | CT32973 | AE003464 | FBgn0035011 |
|  | 354 | 147878_at | 6.6665 | 0.000505 | *CG3640* | CT12219 | AE003464 | FBgn0035042 |
|  | 355 | 147908_at | 9.0009 | 2.61E-05 | *CG13405* | CT32761 | AE003467 | FBgn0035097 |
|  | 356 | 147913_at | 7.1329 | 0.043987 | *CG17142* | CT33412 | AE003467 | FBgn0035113 |
|  | 357 | 147942_at | 3.2652 | 0.011506 | *CG13901* | CT33436 | AE003469 | FBgn0035164 |
|  | 358 | 147961_at | 5.6263 | 0.015001 | *CG1211* | CT2232 | AE003471 | FBgn0035191 |
|  | 359 | 148031_at | 2.4349 | 0.045926 | *CG8970* | CT25794 | AE003474 | FBgn0035320 |
|  | 360 | 148045_at | 5.4289 | 0.004156 | *CG13798* | CT33293 | AE003474 | FBgn0035339 |
|  | 361 | 148053_at | 4.1991 | 0.017163 | *CG16984* | CT34790 | AE003475 | FBgn0035354 |
|  | 362 | 148059_at | 20.928 | 0.015754 | *CG12186* | CT9455 | AE003475 | FBgn0035361 |
|  | 363 | 148072_at | 3.89 | 0.006048 | *CG9965* | CT28101 | AE003476 | FBgn0035381 |
|  | 364 | 148086_at | 13.5576 | 0.002658 | *CG12734* | CT2162 | AE003477 | FBgn0035411 |
|  | 365 | 148106_s_at | 2.978 | 0.028771 | *CG11520* | CT36395 | AE003477 | FBgn0035441 |
|  | 366 | 148123_at | 2.6192 | 0.032641 | *CG14978* | CT34826 | AE003479 | FBgn0035460 |
|  | 367 | 148171_at | 3.7693 | 0.005181 | *CG15017* | CT34870 | AE003481 | FBgn0035535 |
|  | 368 | 148179_at | 3.3568 | 0.019367 | *CG15023* | CT34878 | AE003481 | FBgn0035548 |
|  | 369 | 148212_at | 2.5322 | 0.044515 | *CG4597* | CT14884 | AE003567 | FBgn0035594 |
|  | 370 | 148221_at | 2.9507 | 0.03353 | *CG15214* | CT35147 | AE003566 | FBgn0035606 |
|  | 371 | 148239_at | 61.8641 | 1.33E-08 | *CG13287* | CT32574 | AE003564 | FBgn0035643 |
|  | 372 | 148282_at | 2.6187 | 0.047881 | *CG8270* | CT24471 | AE003562 | FBgn0035703 |
|  | 373 | 148293_at | 2.7022 | 0.028924 | *CG10064* | CT28331 | AE003560 | FBgn0035724 |
|  | 374 | 148317_at | 2.4829 | 0.047983 | *CG14829* | CT34645 | AE003559 | FBgn0035751 |
|  | 375 | 148326_at | 9.4973 | 0.003941 | *CG14834* | CT34650 | AE003559 | FBgn0035768 |
|  | 376 | 148329_at | 5.638 | 0.000246 | *CG8572* | CT14406 | AE003559 | FBgn0035775 |
|  | 377 | 148344_at | 2.8214 | 0.02462 | *CG16998* | CT37725 | AE003558 | FBgn0035795 |
|  | 378 | 148348_at | 14.5226 | 1.84E-05 | *CG7716* | CT23493 | AE003558 | FBgn0035800 |
|  | 379 | 148350_at | 3.3241 | 0.014342 | *CG7515* | CT15934 | AE003558 | FBgn0035802 |
|  | 380 | 148366_at | 3.4427 | 0.011817 | *CG8111* | CT24252 | AE003557 | FBgn0035825 |
|  | 381 | 148379_at | 2.9547 | 0.026484 | *CG8005* | CT24054 | AE003556 | FBgn0035854 |
|  | 382 | 148397_at | 2.9982 | 0.021358 | *CG13669* | CT33104 | AE003555 | FBgn0035883 |
|  | 383 | 148418_at | 4.2302 | 0.002361 | *CG13305* | CT32600 | AE003554 | FBgn0035921 |
|  | 384 | 148424_at | 10.9352 | 0.000568 | *CG13311* | CT32606 | AE003554 | FBgn0035929 |
|  | 385 | 148444_at | 2.591 | 0.037107 | *CG4911* | CT15774 | AE003553 | FBgn0035959 |
|  | 386 | 148452_at | 3.2178 | 0.012676 | *CG4483* | CT14598 | AE003552 | FBgn0035970 |
|  | 387 | 148461_at | 3.1331 | 0.021051 | *CG3982* | CT13237 | AE003552 | FBgn0035988 |
|  | 388 | 148520_at | 2.7328 | 0.031681 | *CG12362* | CT24168 | AE003548 | FBgn0036082 |
|  | 389 | 148526_at | 3.0231 | 0.021518 | *CG18628* | CT41623 | AE003547 | FBgn0036091 |
|  | 390 | 148529_at | 4.1182 | 0.002711 | *CG14153* | CT33756 | AE003547 | FBgn0036094 |
|  | 391 | 148530_at | 2.5991 | 0.037866 | *CG6487* | CT20205 | AE003547 | FBgn0036095 |
|  | 392 | 148581_at | 4.3312 | 0.003716 | *CG7512* | CT22779 | AE003544 | FBgn0036168 |
|  | 393 | 148625_at | 177.3534 | 4.27E-05 | *CG6947* | CT21507 | AE003543 | FBgn0036233 |
|  | 394 | 148627_at | 123.4081 | 4.16E-05 | *CG6793* | CT21079 | AE003542 | FBgn0036242 |
|  | 395 | 148631_at | 3.6167 | 0.009187 | *CG17154* | CT29096 | AE003542 | FBgn0036246 |
|  | 396 | 148636_at | 2.9867 | 0.040957 | *CG9760* | CT27581 | AE003542 | FBgn0036259 |
|  | 397 | 148674_at | 3.2612 | 0.015713 | *CG17666* | CT39001 | AE003540 | FBgn0036311 |
|  | 398 | 148724_at | 2.7093 | 0.048487 | *CG8757* | CT25268 | AE003536 | FBgn0036380 |
|  | 399 | 148726_at | 2.4854 | 0.04883 | *CG13737* | CT33204 | AE003536 | FBgn0036382 |
|  | 400 | 148740_at | 2.8829 | 0.025801 | *CG13484* | CT32851 | AE003536 | FBgn0036406 |
|  | 401 | 148749_at | 956.793 | 0.001044 | *CG9598* | CT27142 | AE003534 | FBgn0036424 |
|  | 402 | 148762_at | 3.1686 | 0.015151 | *CG17177* | CT38112 | AE003533 | FBgn0036440 |
|  | 403 | 148787_at | 3.2564 | 0.047764 | *CG18648* | CT42479 | AE003532 | FBgn0036473 |
|  | 404 | 148805_at | 14.3767 | 0.011522 | *CG7841* | CT23788 | AE003530 | FBgn0036502 |
|  | 405 | 148837_at | 3.1342 | 0.014398 | *CG17029* | CT37791 | AE003529 | FBgn0036551 |
|  | 406 | 148844_at | 2.6684 | 0.036634 | *CG5235* | CT16721 | AE003528 | FBgn0036565 |
|  | 407 | 148845_at | 3.2328 | 0.026704 | *CG13074* | CT32293 | AE003528 | FBgn0036567 |
|  | 408 | 148870_at | 2.835 | 0.025374 | *CG4962* | CT15908 | AE003528 | FBgn0036597 |
|  | 409 | 148893_at | 4.9741 | 0.006149 | *CG13036* | CT32255 | AE003527 | FBgn0036626 |
|  | 410 | 148910_at | 16.5049 | 0.021606 | *CG9692* | CT27398 | AE003526 | FBgn0036654 |
|  | 411 | 148924_at | 6.1638 | 0.000524 | *CG13028* | CT32246 | AE003525 | FBgn0036676 |
|  | 412 | 148936_at | 14.9344 | 0.002477 | *CG6497* | CT20241 | AE003524 | FBgn0036704 |
|  | 413 | 148960_at | 59.2949 | 0.000211 | *CG6333* | CT19808 | AE003524 | FBgn0036731 |
|  | 414 | 148977_at | 6.5993 | 0.004507 | *CG14585* | CT34326 | AE003522 | FBgn0036757 |
|  | 415 | 149018_at | 49.5488 | 0.019783 | *CG18168* | CT40998 | AE003519 | FBgn0036823 |
|  | 416 | 149067_at | 3.2166 | 0.02031 | *CG8786* | CT25334 | AE003516 | FBgn0036897 |
|  | 417 | 149103_at | 5.90E+15 | 0 | *CG17147* | CT33309 | AE003514 | FBgn0036954 |
|  | 418 | 149111_at | 6.1294 | 0.002018 | *CG13817* | CT33316 | AE003514 | FBgn0036963 |
|  | 419 | 149146_at | 2.8587 | 0.024682 | *CG11396* | CT31819 | AE003592 | FBgn0037022 |
|  | 420 | 149152_at | 11.881 | 0.000113 | *CG13256* | CT32513 | AE003592 | FBgn0037033 |
|  | 421 | 149155_at | 3.2078 | 0.013897 | *CG10588* | CT29688 | AE003593 | FBgn0037037 |
|  | 422 | 149176_at | 3.414 | 0.021622 | *CG6968* | CT21581 | AE003594 | FBgn0037077 |
|  | 423 | 149205_at | 3.1315 | 0.014873 | *CG14573* | CT34304 | AE003595 | FBgn0037125 |
|  | 424 | 149249_at | 4.3287 | 0.002285 | *CG14451* | CT34131 | AE003598 | FBgn0037183 |
|  | 425 | 149265_at | 5.2446 | 0.01118 | *CG17439* | CT38533 | AE002665 | FBgn0037210 |
|  | 426 | 149268_at | 2.6156 | 0.04376 | *CG14638* | CT34410 | AE003607 | FBgn0037223 |
|  | 427 | 149297_at | 2.4659 | 0.04975 | *CG1277* | CT2642 | AE003605 | FBgn0037281 |
|  | 428 | 149330_at | 3.8766 | 0.022677 | *CG10609* | CT29730 | AE003603 | FBgn0037324 |
|  | 429 | 149405_at | 6.7278 | 0.001253 | *CG15182* | CT35097 | AE003674 | FBgn0037451 |
|  | 430 | 149409_at | 4.9527 | 0.009314 | *CG2336* | CT7778 | AE003674 | FBgn0037455 |
|  | 431 | 149418_at | 3.9196 | 0.014023 | *CG1988* | CT6344 | AE003673 | FBgn0037464 |
|  | 432 | 149429_at | 4.1062 | 0.011555 | *CG14609* | CT34357 | AE003672 | FBgn0037483 |
|  | 433 | 149435_at | 4.6145 | 0.001408 | *CG10050* | CT28283 | AE003672 | FBgn0037492 |
|  | 434 | 149472_at | 4.7663 | 0.003398 | *CG7918* | CT23924 | AE003677 | FBgn0037546 |
|  | 435 | 149478_at | 2.9636 | 0.02516 | *CG11672* | CT32180 | AE003678 | FBgn0037563 |
|  | 436 | 149482_at | 34.4612 | 0.028439 | *CG9617* | CT27208 | AE003678 | FBgn0037568 |
|  | 437 | 149486_at | 47724.3252 | 1.23E-11 | *CG11698* | CT36735 | AE003678 | FBgn0037572 |
|  | 438 | 149491_at | 5.9762 | 0.00439 | *CG7443* | CT22913 | AE003678 | FBgn0037577 |
|  | 439 | 149507_at | 70.6505 | 6.91E-10 | *CG11744* | CT32623 | AE003679 | FBgn0037596 |
|  | 440 | 149524_at | 2.6866 | 0.038249 | *CG13318* | CT32620 | AE003680 | FBgn0037627 |
|  | 441 | 149535_at | 3.619 | 0.009157 | *CG11977* | CT32138 | AE003681 | FBgn0037650 |
|  | 442 | 149542_at | 2.9569 | 0.042346 | *CG16734* | CT32114 | AE003682 | FBgn0037667 |
|  | 443 | 149574_at | 3.3794 | 0.025071 | *CG8362* | CT24641 | AE003683 | FBgn0037729 |
|  | 444 | 149602_at | 12.2562 | 2.81E-05 | *CG12807* | CT31933 | AE003685 | FBgn0037772 |
|  | 445 | 149613_at | 3.6655 | 0.012638 | *CG12809* | CT31935 | AE003685 | FBgn0037785 |
|  | 446 | 149640_at | 3.8031 | 0.005987 | *CG14690* | CT34476 | AE003688 | FBgn0037828 |
|  | 447 | 149659_at | 3.3205 | 0.030197 | *CG4683* | CT15121 | AE003689 | FBgn0037857 |
|  | 448 | 149691_at | 6.4282 | 0.04149 | *CG14708* | CT34499 | AE003692 | FBgn0037910 |
|  | 449 | 149700_at | 2.4687 | 0.04195 | *CG6834* | CT21175 | AE003692 | FBgn0037935 |
|  | 450 | 149707_at | 2.9206 | 0.02588 | *CG14725* | CT34517 | AE003693 | FBgn0037948 |
|  | 451 | 149721_at | 3.0856 | 0.01817 | *CG10005* | CT28191 | AE003693 | FBgn0037972 |
|  | 452 | 149740_at | 3.9298 | 0.005295 | *CG17404* | CT38423 | AE003695 | FBgn0038001 |
|  | 453 | 149753_at | 4.9097 | 0.014064 | *CG10090* | CT28285 | AE003695 | FBgn0038018 |
|  | 454 | 149754_at | 3.8174 | 0.005744 | *CG17392* | CT38399 | AE003695 | FBgn0038019 |
|  | 455 | 149778_at | 3.5424 | 0.042055 | *CG17207* | CT34543 | AE003696 | FBgn0038051 |
|  | 456 | 149780_at | 4.2459 | 0.004681 | *CG5509* | CT17232 | AE003696 | FBgn0038054 |
|  | 457 | 149804_at | 19.2874 | 0.00306 | *CG14387* | CT34036 | AE003697 | FBgn0038089 |
|  | 458 | 149824_at | 3.2953 | 0.011058 | *CG14380* | CT34027 | AE003699 | FBgn0038124 |
|  | 459 | 149834_at | 443.7084 | 0.00225 | *CG8795* | CT25350 | AE003699 | FBgn0038139 |
|  | 460 | 149851_at | 8.0425 | 0.000166 | *CG14368* | CT34005 | AE003701 | FBgn0038162 |
|  | 461 | 149871_at | 3.2247 | 0.011876 | *CG3153* | CT10556 | AE003703 | FBgn0038198 |
|  | 462 | 149873_at | 4.7374 | 0.002923 | *CG9920* | CT27932 | AE003703 | FBgn0038200 |
|  | 463 | 149905_at | 2.5065 | 0.043103 | *CG14852* | CT34668 | AE003705 | FBgn0038242 |
|  | 464 | 149925_at | 45.6965 | 5.56E-06 | *CG14860* | CT34677 | AE003706 | FBgn0038273 |
|  | 465 | 149928_at | 2.7007 | 0.030644 | *CG3843* | CT12845 | AE003707 | FBgn0038281 |
|  | 466 | 149938_at | 3.1238 | 0.017382 | *CG3984* | CT12985 | AE003707 | FBgn0038291 |
|  | 467 | 149939_at | 3.8788 | 0.028476 | *CG4154* | CT13386 | AE003707 | FBgn0038295 |
|  | 468 | 149954_at | 2.73 | 0.030835 | *CG14867* | CT34686 | AE003708 | FBgn0038333 |
|  | 469 | 149963_at | 2.934 | 0.020327 | *CG5213* | CT16671 | AE003709 | FBgn0038345 |
|  | 470 | 149975_at | 2.6441 | 0.033324 | *CG9593* | CT27114 | AE003710 | FBgn0038365 |
|  | 471 | 150011_at | 2.591 | 0.045325 | *CG10317* | CT28975 | AE003713 | FBgn0038423 |
|  | 472 | 150044_at | 3.3321 | 0.013109 | *CG18213* | CT41204 | AE003716 | FBgn0038470 |
|  | 473 | 150052_at | 8.57E+15 | 1.11E-16 | *CG5240* | CT16729 | AE003716 | FBgn0038483 |
|  | 474 | 150057_at | 3.7758 | 0.004994 | *CG12265* | CT16845 | AE003716 | FBgn0038489 |
|  | 475 | 150078_at | 3.1975 | 0.030706 | *CG12536* | CT33960 | AE003718 | FBgn0038513 |
|  | 476 | 150098_at | 4.4886 | 0.008888 | *CG7397* | CT22754 | AE003719 | FBgn0038543 |
|  | 477 | 150100_at | 14.0131 | 1.60E-05 | *CG7713* | CT23489 | AE003719 | FBgn0038545 |
|  | 478 | 150102_at | 27.5579 | 0.00133 | *CG17802* | CT39444 | AE003719 | FBgn0038549 |
|  | 479 | 150168_at | 6.1474 | 0.00058 | *CG14292* | CT33921 | AE003724 | FBgn0038658 |
|  | 480 | 150199_at | 3.9265 | 0.021705 | *CG15025* | CT34882 | AE003726 | FBgn0038709 |
|  | 481 | 150204_at | 3.4405 | 0.033652 | *CG7333* | CT22619 | AE003727 | FBgn0038715 |
|  | 482 | 150207_at | 2.6305 | 0.035414 | *CG16727* | CT37205 | AE003727 | FBgn0038719 |
|  | 483 | 150234_at | 3.327 | 0.012356 | *CG4794* | CT15415 | AE003728 | FBgn0038757 |
|  | 484 | 150257_at | 2.8174 | 0.037582 | *CG5097* | CT16353 | AE003730 | FBgn0038790 |
|  | 485 | 150279_at | 9.55E+15 | 0 | *CG15695* | CT35896 | AE003732 | FBgn0038832 |
|  | 486 | 150282_at | 5.6185 | 0.002415 | *CG17275* | CT35908 | AE003732 | FBgn0038836 |
|  | 487 | 150302_at | 4.9416 | 0.038624 | *CG5810* | CT18214 | AE003733 | FBgn0038866 |
|  | 488 | 150336_at | 3.237 | 0.01538 | *CG17819* | CT39518 | AE003736 | FBgn0038915 |
|  | 489 | 150339_at | 2.6992 | 0.049102 | *CG6690* | CT20779 | AE003736 | FBgn0038918 |
|  | 490 | 150390_at | 119.6698 | 0.001377 | *CG17625* | CT33351 | AE003740 | FBgn0039002 |
|  | 491 | 150458_at | 2.7744 | 0.031664 | *CG10183* | CT28645 | AE003744 | FBgn0039093 |
|  | 492 | 150461_at | 3.6965 | 0.019035 | *CG13820* | CT33320 | AE003744 | FBgn0039097 |
|  | 493 | 150473_at | 3.8979 | 0.009488 | *CG5338* | CT16966 | AE003745 | FBgn0039129 |
|  | 494 | 150480_at | 3.9375 | 0.007896 | *CG10694* | CT29977 | AE003746 | FBgn0039147 |
|  | 495 | 150489_at | 2.5379 | 0.037381 | *CG13606* | CT32991 | AE003746 | FBgn0039161 |
|  | 496 | 150500_at | 62.2326 | 2.06E-05 | *CG5715* | CT17981 | AE003747 | FBgn0039180 |
|  | 497 | 150504_at | 5.412 | 0.004841 | *CG17784* | CT33005 | AE003747 | FBgn0039192 |
|  | 498 | 150543_at | 3.5231 | 0.012284 | *CG13648* | CT33044 | AE003750 | FBgn0039257 |
|  | 499 | 150564_at | 3.4614 | 0.010605 | *CG13664* | CT33093 | AE003751 | FBgn0039295 |
|  | 500 | 150574_at | 7.2145 | 2.23E-05 | *CG11891* | CT37044 | AE003751 | FBgn0039309 |
|  | 501 | 150586_at | 4.4876 | 0.001487 | *CG10553* | CT29612 | AE003751 | FBgn0039324 |
|  | 502 | 150616_at | 3.0352 | 0.0208 | *CG4964* | CT15930 | AE003753 | FBgn0039372 |
|  | 503 | 150645_at | 4.7585 | 0.013836 | *CG14551* | CT34281 | AE003754 | FBgn0039408 |
|  | 504 | 150657_at | 2.6141 | 0.033273 | *CG14239* | CT33859 | AE003756 | FBgn0039424 |
|  | 505 | 150675_at | 237.9566 | 0.000439 | *CG14248* | CT33868 | AE003757 | FBgn0039445 |
|  | 506 | 150699_at | 12.4343 | 0.007567 | *CG6295* | CT19704 | AE003758 | FBgn0039471 |
|  | 507 | 150709_at | 4.0514 | 0.003025 | *CG14259* | CT33879 | AE003759 | FBgn0039483 |
|  | 508 | 150711_at | 3.4948 | 0.007652 | *CG17189* | CT33881 | AE003759 | FBgn0039485 |
|  | 509 | 150738_at | 4.851 | 0.004566 | *CG5639* | CT17816 | AE003761 | FBgn0039527 |
|  | 510 | 150768_at | 2.7681 | 0.042362 | *CG12852* | CT31990 | AE003764 | FBgn0039573 |
|  | 511 | 150793_at | 6.573 | 0.001669 | *CG11828* | CT4648 | AE003767 | FBgn0039616 |
|  | 512 | 150795_at | 3.7488 | 0.030623 | *CG14519* | CT34246 | AE003767 | FBgn0039619 |
|  | 513 | 150857_at | 2.8607 | 0.033708 | *CG15516* | CT35629 | AE003771 | FBgn0039720 |
|  | 514 | 150861_at | 2.4649 | 0.043498 | *CG15523* | CT35636 | AE003771 | FBgn0039727 |
|  | 515 | 150867_at | 15.7461 | 0.010272 | *CG7912* | CT6603 | AE003772 | FBgn0039736 |
|  | 516 | 150887_at | 13.6304 | 0.006729 | *CG15533* | CT35648 | AE003773 | FBgn0039768 |
|  | 517 | 150935_at | 34.6094 | 4.54E-07 | *CG3669* | CT6812 | AE003777 | FBgn0039838 |
|  | 518 | 150947_at | 14.4859 | 0.011362 | *CG11518* | CT35373 | AE003778 | FBgn0039853 |
|  | 519 | 150951_at | 4.4056 | 0.005842 | *CG1792* | CT5380 | AE003779 | FBgn0039860 |
|  | 520 | 151004_at | 4.0829 | 0.007579 | *CG13236* | CT32480 | AE003781 | FBgn0040517 |
|  | 521 | 151039_at | 11.4172 | 0.000219 | *CG14378* | CT34017 | AE003700 | FBgn0040552 |
|  | 522 | 151062_at | 3.3989 | 0.009542 | *CG5115* | CT16431 | AE003730 | FBgn0040574 |
|  | 523 | 151072_i_at | 3.1921 | 0.034592 | *CG13858* | CT33372 | AE003738 | FBgn0040585 |
|  | 524 | 151082_at | 3.461 | 0.014667 | *CG11945* | CT37107 | AE003745 | FBgn0040595 |
|  | 525 | 151118_at | 4.8973 | 0.003389 | *CG12451* | CT32505 | AE003591 | FBgn0040633 |
|  | 526 | 151119_at | 3.1965 | 0.01255 | *CG4186* | CT13780 | AE003592 | FBgn0040634 |
|  | 527 | 151125_at | 5.4346 | 0.002317 | *CG18618* | CT40619 | AE003598 | FBgn0040641 |
|  | 528 | 151130_at | 4.2056 | 0.025551 | *CG14580* | CT34315 | AE003568 | FBgn0040646 |
|  | 529 | 151132_at | 10.6441 | 0.00094 | *CG11666* | CT35515 | AE003569 | FBgn0040648 |
|  | 530 | 151149_at | 13.1379 | 0.004788 | *CG14602* | CT34350 | AE003672 | FBgn0040669 |
|  | 531 | 151150_at | 7.6834 | 0.025523 | *CG14612* | CT34360 | AE003672 | FBgn0040670 |
|  | 532 | 151170_at | 64.7616 | 3.87E-05 | *CG13799* | CT33294 | AE003474 | FBgn0040693 |
|  | 533 | 151174_at | 148.0696 | 1.74E-09 | *CG18676* | CT42631 | AE003480 | FBgn0040697 |
|  | 534 | 151181_at | 3.4464 | 0.017316 | *CG11930* | CT35792 | AE003575 | FBgn0040704 |
|  | 535 | 151245_at | 3.5225 | 0.038085 | *CG12939* | CT32087 | AE003828 | FBgn0040770 |
|  | 536 | 151259_at | 3.357 | 0.017986 | *CG13378* | CT32718 | AE003519 | FBgn0040788 |
|  | 537 | 151293_f_at | 2.767 | 0.035938 | *CG14139* | CT33740 | AE003544 | FBgn0040819 |
|  | 538 | 151299_at | 2.9879 | 0.020362 | *CG14178* | CT33783 | AE003551 | FBgn0040825 |
|  | 539 | 151332_r_at | 3.0486 | 0.024243 | *CG11366* | CT31712 | AE003488 | FBgn0040857 |
|  | 540 | 151347_i_at | 2.6058 | 0.044375 | *CG15600* | CT35723 | AE003499 | FBgn0040874 |
|  | 541 | 151357_at | 2.4759 | 0.044011 | *CG15037* | CT34900 | AE003510 | FBgn0040884 |
|  | 542 | 151384_at | 2.7104 | 0.033166 | *CG15775* | CT36034 | AE003435 | FBgn0040915 |
|  | 543 | 151386_at | 2.5383 | 0.040083 | *CG14447* | CT34120 | AE003436 | FBgn0040917 |
|  | 544 | 151402_at | 3.2688 | 0.014944 | *CG18535* | CT35304 | AE003447 | FBgn0040935 |
|  | 545 | 151412_r_at | 6.5736 | 0.000223 | *CG12639* | CT35268 | AE003450 | FBgn0040945 |
|  | 546 | 151423_at | 3.2199 | 0.045272 | *CG15867* | CT32330 | AE003622 | FBgn0040961 |
|  | 547 | 151426_at | 3.6298 | 0.019077 | *CG13130* | CT32368 | AE003627 | FBgn0040965 |
|  | 548 | 151432_at | 9.2306 | 0.001204 | *CG15494* | CT35596 | AE003636 | FBgn0040971 |
|  | 549 | 151433_at | 4.7388 | 0.000992 | *CG16978* | CT35582 | AE003639 | FBgn0040972 |
|  | 550 | 151492_at | 4.7968 | 0.001141 | *CG11773* | CT36855 |  | FBgn0037628 |
|  | 551 | 151493_at | 4.6614 | 0.002744 | *pnt* | CT37466 |  | FBgn0003118 |
|  | 552 | 151524_r_at | 3.8195 | 0.005414 | *CG6670* | CT2716 |  | FBgn0038913 |
|  | 553 | 151547_r_at | 2.5788 | 0.038759 | *CG8677* | CT5294 |  | FBgn0032936 |
|  | 554 | 151550_s_at | 6.4567 | 0.000456 | *CG9708* | CT2745 |  | FBgn0039791 |
|  | 555 | 151551_i_at | 1.16E+16 | 0 | *CG8215* | CT2412 |  | FBgn0034038 |
|  | 556 | 151571_at | 2.4131 | 0.049979 | *NK7.1* | CT24869 |  | FBgn0024321 |
|  | 557 | 151574_r_at | 2.5639 | 0.038198 | *CG6040* | CT18854 |  | FBgn0038679 |
|  | 558 | 151637_at | 4.7876 | 0.000917 | *CG7540* | CT2379 |  | FBgn0037092 |
|  | 559 | 151678_at | 3.2408 | 0.013531 | *CG11905* | CT27328 |  | FBgn0036678 |
|  | 560 | 151818_at | 3.1045 | 0.020129 | *CG7051* | CT21815 | AE003467 | FBgn0035100 |
|  | 561 | 151838_at | 2.8208 | 0.023312 | *CG2789* | CT9517 | AE003589 | FBgn0031263 |
|  | 562 | 151842_at | 2.6601 | 0.039157 | *BcDNA:GH02250* | CT6423 | AE003772 | FBgn0027614 |
|  | 563 | 151871_at | 2.6354 | 0.031771 | *BcDNA:GH02712* | CT6764 | AE003840 | FBgn0027604 |
|  | 564 | 151885_at | 3.5335 | 0.007106 | *BcDNA:GH02901* | CT25792 | AE003497 | FBgn0027601 |
|  | 565 | 151897_at | 3.0094 | 0.01701 | *CG4919* | CT15810 | AE003740 | FBgn0039001 |
|  | 566 | 151899_at | 2.556 | 0.036268 | *CG11796* | CT36593 | AE003591 | FBgn0036992 |
|  | 567 | 151926_at | 3.3223 | 0.010095 | *CG4673* | CT15047 | AE003752 | FBgn0039348 |
|  | 568 | 151927_at | 6.373 | 7.78E-05 | *CG4784* | CT15377 | AE003527 | FBgn0036619 |
|  | 569 | 151950_at | 6.8731 | 0.008978 | *Sptr* | CT7144 | AE003444 | FBgn0014032 |
|  | 570 | 151980_at | 5.8037 | 0.00204 | *CG5150* | CT16497 | AE003565 | FBgn0035620 |
|  | 571 | 152060_at | 3.9781 | 0.003455 | *angel* | CT17360 | AE003461 | FBgn0016762 |
|  | 572 | 152108_at | 2.5848 | 0.045727 | *BcDNA:GH07346* | CT18234 | AE003759 | FBgn0027574 |
|  | 573 | 152113_at | 5.4363 | 0.000328 | *ana* | CT4622 | AE003834 | FBgn0011746 |
|  | 574 | 152123_at | 2.8247 | 0.023161 | *CG6218* | CT19478 | AE003708 | FBgn0038321 |
|  | 575 | 152166_at | 4.1165 | 0.002719 | *CG9377* | CT26601 | AE003639 | FBgn0032507 |
|  | 576 | 152180_at | 76.1349 | 0.012964 | *CG7669* | CT23139 | AE003721 | FBgn0038607 |
|  | 577 | 152202_at | 1068.5308 | 5.25E-05 | *BG:DS07295.3* | CT13219 | AE003646 | FBgn0027559 |
|  | 578 | 152224_at | 3.5835 | 0.014003 | *CG8508* | CT24847 | AE003699 | FBgn0038123 |
|  | 579 | 152238_at | 4.4084 | 0.004158 | *CG6004* | CT18872 | AE003543 | FBgn0036203 |
|  | 580 | 152261_at | 5.1789 | 0.000492 | *CAH1* | CT23642 | AE003641 | FBgn0027844 |
|  | 581 | 152270_at | 4.9431 | 0.001393 | *CG4329* | CT14129 | AE003458 | FBgn0034745 |
|  | 582 | 152308_at | 5.3624 | 0.000369 | *CG10433* | CT29298 | AE003454 | FBgn0034638 |
|  | 583 | 152353_at | 3.0294 | 0.027615 | *CG4525* | CT14672 | AE003710 | FBgn0038358 |
|  | 584 | 152389_at | 2.8584 | 0.030383 | *CG14355* | CT33990 | AE003703 | FBgn0038208 |
|  | 585 | 152425_at | 2.9673 | 0.018251 | *CG3174* | CT10647 | AE003789 | FBgn0033079 |
|  | 586 | 152441_at | 6.5008 | 9.97E-05 | *CG4744* | CT15313 | AE003818 | FBgn0033834 |
|  | 587 | 152457_at | 4.0427 | 0.003068 | *CG3672* | CT12317 | AE003552 | FBgn0035985 |
|  | 588 | 152459_at | 2.8379 | 0.0228 | *CG10854* | CT30389 | AE003479 | FBgn0035463 |
|  | 589 | 152477_at | 3.3083 | 0.024444 | *CG6306* | CT19682 | AE003508 | FBgn0030920 |
|  | 590 | 152513_at | 4.659 | 0.001127 | *CG10513* | CT29500 | AE003751 | FBgn0039311 |
|  | 591 | 152552_at | 4.4368 | 0.001614 | *CG6166* | CT19364 | AE003756 | FBgn0039423 |
|  | 592 | 152554_at | 2.4906 | 0.042443 | *CG5845* | CT18323 | AE003736 | FBgn0038899 |
|  | 593 | 152622_at | 3.7274 | 0.005274 | *CG2641* | CT8941 | AE003676 | FBgn0037518 |
|  | 594 | 152673_at | 3.1963 | 0.012452 | *cry* | CT12574 | AE003725 | FBgn0025680 |
|  | 595 | 152682_at | 13.7297 | 5.05E-09 | *Slob* | CT20985 | AE003618 | FBgn0024290 |
|  | 596 | 152683_at | 2.5368 | 0.038661 | *CG17664* | CT38999 | AE003461 | FBgn0034883 |
|  | 597 | 152693_at | 3.0851 | 0.017939 | *CG4291* | CT13138 | AE003588 | FBgn0031287 |
|  | 598 | 152712_at | 2.9201 | 0.031927 | *CG4983* | CT15981 | AE003633 | FBgn0032371 |
|  | 599 | 152713_at | 4.0766 | 0.002983 | *CG9363* | CT26609 | AE003682 | FBgn0037697 |
|  | 600 | 152718_at | 10.9076 | 0.001142 | *CG6614* | CT20550 | AE003633 | FBgn0032369 |
|  | 601 | 152726_at | 3.5367 | 0.018778 | *CG8708* | CT9353 | AE003838 | FBgn0033271 |
|  | 602 | 152732_at | 3.0539 | 0.015791 | *CG5955* | CT18717 | AE003591 | FBgn0036997 |
|  | 603 | 152739_at | 2.5222 | 0.039601 | *CG15892* | CT34109 | AE003437 | FBgn0029859 |
|  | 604 | 152742_at | 2.9945 | 0.048181 | *CG10999* | CT30805 | AE003601 | FBgn0037381 |
|  | 605 | 152755_at | 2.6162 | 0.032804 | *CG8709* | CT9407 | AE003838 | FBgn0033269 |
|  | 606 | 152763_at | 3.8285 | 0.006967 | *CG2616* | CT8885 | AE003676 | FBgn0037512 |
|  | 607 | 152768_at | 4.1717 | 0.003154 | *CG4945* | CT15864 | AE003806 | FBgn0034137 |
|  | 608 | 152789_at | 5.1852 | 0.000587 | *CG13458* | CT32821 | AE003532 | FBgn0036479 |
|  | 609 | 152805_at | 6.6687 | 0.002756 | *BG:DS06874.1* | CT15105 | AE003645 | FBgn0028870 |
|  | 610 | 152819_at | 3.912 | 0.003803 | *CG11407* | CT31802 | AE003727 | FBgn0038733 |
|  | 611 | 152821_at | 3.8242 | 0.004394 | *CG6305* | CT19746 | AE003817 | FBgn0033869 |
|  | 612 | 152822_at | 2.7948 | 0.024347 | *CG4523* | CT14670 | AE003438 | FBgn0029891 |
|  | 613 | 152855_at | 2.7986 | 0.046611 | *CG11546* | CT9385 | AE003838 | FBgn0033270 |
|  | 614 | 152883_at | 3.1975 | 0.018361 | *CG3330* | CT11167 | AE003760 | FBgn0039511 |
|  | 615 | 152912_at | 2.7418 | 0.030273 | *CG13773* | CT33261 | AE003615 | FBgn0031867 |
|  | 616 | 152943_at | 4.2205 | 0.003061 | *CG3328* | CT11181 | AE003463 | FBgn0034985 |
|  | 617 | 152958_at | 3.0725 | 0.015309 | *Cyp4d21* | CT20878 | AE003618 | FBgn0031925 |
|  | 618 | 153006_at | 2.4422 | 0.045139 | *CG17012* | CT37749 | AE003583 | FBgn0031406 |
|  | 619 | 153029_at | 2.5416 | 0.044877 | *fln* | CT22901 | AE003515 | FBgn0005633 |
|  | 620 | 153054_at | 2.5344 | 0.037601 | *CG15132* | CT35027 | AE003653 | FBgn0032617 |
|  | 621 | 153132_at | 2.6114 | 0.048354 | *CG4043* | CT13404 | AE003687 | FBgn0037815 |
|  | 622 | 153174_at | 2.5776 | 0.042511 | *CG9925* | CT27942 | AE003702 | FBgn0038191 |
|  | 623 | 153229_at | 2.5758 | 0.044307 | *CG15746* | CT35995 | AE003492 | FBgn0040863 |
|  | 624 | 153272_at | 5.8485 | 0.001738 | *CG10519* | CT29521 | AE003593 | FBgn0037055 |
|  | 625 | 153306_at | 2.8438 | 0.022431 | *CG8863* | CT25440 | AE003700 | FBgn0038145 |
|  | 626 | 153322_at | 2.7603 | 0.025945 | *CG3980* | CT13231 | AE003578 | FBgn0031575 |
|  | 627 | 153334_at | 2.505 | 0.040074 | *CG2611* | CT8871 | AE003667 | FBgn0032871 |
|  | 628 | 153356_at | 2.5466 | 0.039012 | *CG6153* | CT19326 | AE003637 | FBgn0032445 |
|  | 629 | 153379_at | 4.6653 | 0.0012 | *CG7245* | CT22349 | AE003583 | FBgn0031416 |
|  | 630 | 153432_at | 3.2128 | 0.012114 | *Phas1* | CT9265 | AE003579 | FBgn0022073 |
|  | 631 | 153445_at | 2.8874 | 0.025626 | *CG12114* | CT6766 | AE003777 | FBgn0039837 |
|  | 632 | 153452_at | 2.5329 | 0.046011 | *CG11578* | CT36508 | AE003844 | FBgn0039891 |
|  | 633 | 153464_at | 2.4949 | 0.046191 | *CG4913* | CT15784 | AE003708 | FBgn0038317 |
|  | 634 | 153537_at | 3.0469 | 0.024894 | *CG15011* | CT34864 | AE003480 | FBgn0035518 |
|  | 635 | 153575_at | 2.6291 | 0.032106 | *CG3402* | CT11431 | AE003469 | FBgn0035148 |
|  | 636 | 153595_at | 4.5376 | 0.003538 | *CG6927* | CT21430 | AE003432 | FBgn0029733 |
|  | 637 | 153610_at | 2.9298 | 0.019429 | *CG3488* | CT11757 | AE003580 | FBgn0014906 |
|  | 638 | 153731_at | 3.0517 | 0.017256 | *Hsp26* | CT13810 | AE003552 | FBgn0001225 |
|  | 639 | 153732_at | 3.075 | 0.023373 | *CG1368* | CT3032 | AE003495 | FBgn0030539 |
|  | 640 | 153742_at | 5.6891 | 0.000314 | *polo* | CT20542 | AE003514 | FBgn0003124 |
|  | 641 | 153746_at | 3.6422 | 0.013594 | *pie* | CT17020 | AE003628 | FBgn0005683 |
|  | 642 | 153770_at | 2.5032 | 0.039604 | *CG10855* | CT30391 | AE003479 | FBgn0035461 |
|  | 643 | 153778_at | 3.2884 | 0.01068 | *Ptpmeg* | CT2090 | AE003468 | FBgn0035133 |
|  | 644 | 153787_at | 4.7692 | 0.002984 | *CG10722* | CT30053 | AE003665 | FBgn0032848 |
|  | 645 | 153804_at | 2.6682 | 0.038135 | *CG11881* | CT34233 | AE003768 | FBgn0039638 |
|  | 646 | 153832_at | 4.4422 | 0.00168 | *Rhk* | CT27613 | AE003502 | FBgn0028698 |
|  | 647 | 153930_at | 66.4205 | 0.003863 | *CG5224* | CT16701 | AE003799 | FBgn0034354 |
|  | 648 | 153951_at | 3.0525 | 0.038059 | *ImpE2* | CT6017 | AE003478 | FBgn0001254 |
|  | 649 | 153975_at | 3.2492 | 0.011812 | *CG4004* | CT13285 | AE003490 | FBgn0030418 |
|  | 650 | 153978_at | 3.7585 | 0.006055 | *CG1248* | CT2456 | AE003475 | FBgn0035363 |
|  | 651 | 154146_at | 3.9025 | 0.005268 | *knrl* | CT15285 | AE003591 | FBgn0001323 |
|  | 652 | 154151_at | 2.5594 | 0.040297 | *CG12075* | CT5152 | AE003445 | FBgn0030065 |
|  | 653 | 154188_at | 3.4438 | 0.008247 | *CG4991* | CT15971 | AE003504 | FBgn0030817 |
|  | 654 | 154215_at | 4.9778 | 0.000846 | *CG9640* | CT27246 | AE003804 | FBgn0034182 |
|  | 655 | 154292_at | 5.8381 | 0.001407 | *CG4735* | CT15271 | AE003462 | FBgn0034912 |
|  | 656 | 154375_at | 4.5462 | 0.049713 | *fs(1)Ya* | CT9213 | AE003425 | FBgn0000927 |
|  | 657 | 154417_at | 2.9513 | 0.033504 | *CG3101* | CT7928 | AE003447 | FBgn0030146 |
|  | 658 | 154460_at | 4.6463 | 0.001625 | *CG12047* | CT3445 | AE003495 | FBgn0030557 |
|  | 659 | 154476_at | 2.6313 | 0.031991 | *CG14215* | CT33828 | AE003512 | FBgn0031052 |
|  | 660 | 154496_at | 5.896 | 0.004673 | *CG11261* | CT31435 | AE003539 | FBgn0036332 |
|  | 661 | 154528_at | 3.1259 | 0.017327 | *opa* | CT1819 | AE003604 | FBgn0003002 |
|  | 662 | 154556_at | 2.5906 | 0.039086 | *tos* | CT29158 | AE003660 | FBgn0015553 |
|  | 663 | 154588_at | 2.7987 | 0.025955 | *LamC* | CT28479 | AE003814 | FBgn0010397 |
|  | 664 | 154595_at | 3.5432 | 0.007557 | *CG7526* | CT16044 | AE003558 | FBgn0035798 |
|  | 665 | 154655_at | 3.2573 | 0.039975 | *l(2)35Fe* | CT18240 | AE003650 | FBgn0001995 |
|  | 666 | 154691_at | 2.6414 | 0.037279 | *CG3502* | CT11809 | AE003460 | FBgn0034847 |
|  | 667 | 154813_at | 2.8565 | 0.022248 | *CG12878* | CT32022 | AE003762 | FBgn0039542 |
|  | 668 | 154867_at | 6.5963 | 0.000888 | *CG1070* | CT1279 | AE003672 | FBgn0037471 |
|  | 669 | 154916_at | 2.4026 | 0.046824 | *CG7417* | CT22745 | AE003796 | FBgn0034431 |
|  | 670 | 154936_at | 6.8126 | 4.03E-05 | *CG9285* | CT26455 | AE003701 | FBgn0038184 |
|  | 671 | 154963_at | 2.5878 | 0.034396 | *CG10237* | CT28775 | AE003662 | FBgn0032783 |
|  | 672 | 154971_at | 3.0158 | 0.01905 | *CG5126* | CT16441 | AE003587 | FBgn0031320 |
|  | 673 | 154992_at | 2.8068 | 0.039156 | *CG16892* | CT35395 | AE003446 | FBgn0030122 |
|  | 674 | 155083_at | 2.924 | 0.020337 | *sas* | CT8243 | AE003672 | FBgn0002306 |
|  | 675 | 155103_at | 4.9294 | 0.013873 | *r-l* | CT11942 | AE003733 | FBgn0003257 |
|  | 676 | 155129_at | 509.5599 | 0.032551 | *CG4925* | CT15754 | AE003527 | FBgn0036614 |
|  | 677 | 155130_at | 2.749 | 0.049617 | *CG3060* | CT10276 | AE003462 | FBgn0034947 |
|  | 678 | 155155_at | 5.6933 | 0.00147 | *CG12942* | CT32090 | AE003828 | FBgn0033569 |

All genes identified by our ANOVA screen (LD and DD datasets) possessing significant changes in expression over time. Listed are all probe sets that passed our initial ANOVA screen (sets exhibit a nonadjusted ANOVA *p*-value of 0.05 or less). ANOVA values were calculated using a Matlab script; see Methods for details.

doi:10.1371/journal.pcbi.0030208.t001
